# LncPTEC mediated homocysteine accumulation elevates oxidative stress via UBQLN1-dependent MTHFD1 ubiquitination in DKD

**DOI:** 10.1038/s41419-026-09093-2

**Published:** 2026-07-07

**Authors:** Qijia Wang, Tianhui Wu, Peiling Li, He Zha, Keqian Wu, Handeng Liu, Rui Peng, Xuemei Peng, Ziyue Lin, Dan Lv, Xiaohui Liao, Yan Sun, Zheng Zhang

**Affiliations:** 1 Department of Nephrology, The Second Affiliated Hospital, Chongqing Medical University, Chongqing, China; 2 Department of Cell Biology and Genetics, Basic Medical College, Chongqing Medical University, Chongqing, China; 3 School of Public Health and Laboratory, Qilu Medical University, Zibo, Shandong China; 4 Department of Laboratory Medicine, The First People’s Hospital of Zunyi (The Third Affiliated Hospital of Zunyi Medical University), Zunyi, Guizhou China; 5 Laboratory of Tissue and Cell Biology, Experimental Teaching and Management Center, Chongqing Medical University, Chongqing, China; 6 Department of Bioinformatics, Chongqing Medical University, Chongqing, China; 7 Department of Neurology, Chongqing General Hospital (The Chongqing General Hospital of Chongqing University), Chongqing, China

**Keywords:** Molecular biology, Non-coding RNAs

## Abstract

Diabetic kidney disease (DKD) is a leading cause of end-stage kidney disease and chronic kidney disease. Oxidative stress, a key driver of renal fibrosis and a hallmark of DKD pathological changes, has been extensively studied for its role in DKD progression. However, its specific mechanisms remain unclear. Here, we show that homocysteine (Hcy) accumulation in proximal tubular epithelial cells (PTECs) is a significant contributor to mitochondrial oxidative stress in DKD. Through single-cell RNA sequencing (scRNA-seq) screening, we identify lncPTEC, a DKD-associated long non-coding RNA (lncRNA) from the PTEC cluster. Notably, we find that upregulated lncPTEC correlates with elevated albuminuria in DKD patients and exacerbates mitochondrial oxidative stress, epithelial-mesenchymal transition (EMT) and renal tubular fibrosis both in vitro and in vivo. Mechanistically, lncPTEC is transcriptionally upregulated by the transcription factor specificity protein 1 (SP1) under hyperglycemic conditions. Furthermore, lncPTEC directly interacts with the established key factor of Hcy metabolism, methylenetetrahydrofolate dehydrogenase 1 (MTHFD1), promoting its ubiquitination and degradation via the ubiquitination-related protein UBQLN1. This process leads to Hcy accumulation, mitochondrial oxidative stress, and subsequent DKD progression. Hence, our findings elucidate the role of the lncPTEC/MTHFD1 axis in Hcy-mediated mitochondrial oxidative stress, offering potential diagnostic biomarkers and therapeutic targets for DKD.

**Figure:**
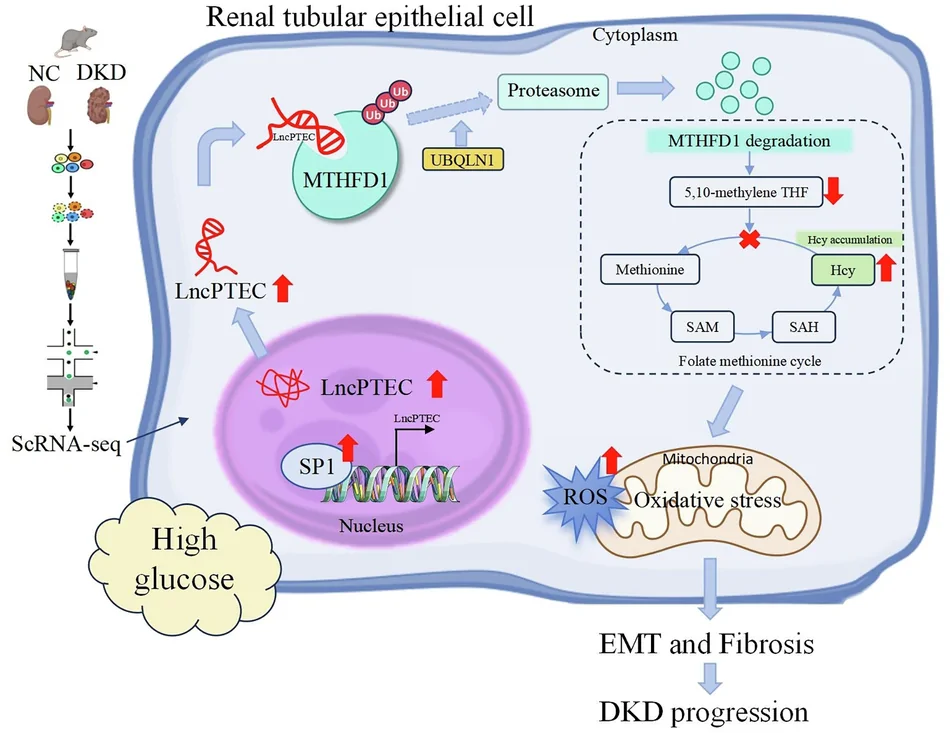

## Introduction

Diabetic kidney disease (DKD), a prevalent complication of diabetes mellitus (DM), is the primary cause of end-stage kidney disease (ESKD), heavily contributing to morbidity, mortality, and economic burden worldwide. Numerous studies have indicated that epithelial-mesenchymal transition (EMT) of renal tubular epithelial cells occurs in DKD, leading to increased extracellular matrix (ECM) synthesis, which progresses to renal tubulointerstitial fibrosis and eventually results in renal failure [1–3]. Recent experimental evidence indicates that proximal tubular epithelial cells, known for their high mitochondrial content, are susceptible to mitochondrial dysfunction and oxidative stress, which can result in DKD [4–7]. However, the details of how oxidative stress promotes the pathophysiological processes underlying the hyperglycemic response in tubular epithelial cells are not fully understood. Recent research indicates that metabolite buildup can enhance oxidative stress in DKD [8].

It has been established that hyperhomocysteinemia is frequently observed in patients with DKD [9, 10]. Homocysteine (Hcy), the sulfur-containing amino acid formed during methionine metabolism, plays a major part in inducing cellular and molecular oxidative damage. Oxidative stress induced by Hcy may stem from multiple mechanisms, including direct ROS generation through autooxidation in the presence of transition metals, activation of pro-oxidant pathways, or impairment of antioxidant defense systems [11]. Also, Chou et al. reported that excessively high Hcy concentrations can elevate mitochondrial NO and ROS production by modifying NADPH oxidase and nitric oxide synthase activities [12]. Although studies indicate that elevated Hcy significantly contributes to oxidative stress, the specific mechanisms by which Hcy contributes to the oxidative stress associated with DKD remain unclear.

Long non-coding RNAs (lncRNAs) represent a class of RNA molecules longer than 200 nucleotides that lack protein-coding capacity. Evidence indicates that lncRNAs are involved in human diseases, including DKD [13–15]. Recent findings suggest that lncRNA 1700020I14Rik mitigates fibrosis in DKD by modulating the miR-34a-5p/Sirt1/HIF-1α signaling axis [16], while lncRNA Rpph1 modulates the inflammatory microenvironment in DKD by influencing the Gal-3/Mek/Erk signaling pathway [17]. Intriguingly, Ye et al. observed that lncRNA SOX2OT mitigates oxidative stress and apoptosis in renal tubular epithelial cells by enhancing SIRT1 expression in DKD [18]; Huang et al. indicated that lncRNA SNHG14 exacerbates renal tubular injury in DKD patients by modulating cell apoptosis, oxidative stress, inflammation, and fibrosis through the miR-483-5p/HDAC4 axis [19], highlighting the significant role of lncRNAs in DKD-related oxidative stress. Importantly, Li et al. reported that the downregulation of lncRNA MEG3 has been shown to mitigate Hcy-induced fibrosis in cardiac fibroblasts [20], indicating a potential role for lncRNAs in Hcy accumulation in DKD.

Our study identified that Hcy accumulation in proximal tubular epithelial cells significantly contributes to mitochondrial oxidative stress in DKD. In addition, single-cell RNA sequencing (scRNA-seq) uncovered an elevated long non-coding RNA, lncPTEC (1700112D23Rik), specific to the PTEC cluster in DKD. lncPTEC was transcriptionally upregulated by specificity protein 1 (SP1), leading to Hcy-triggered oxidative stress and EMT in tubular epithelial cells under DKD conditions both in vivo and in vitro. Mechanistically, lncPTEC could form an RNA-protein complex with Hcy metabolism-related protein methylenetetrahydrofolate dehydrogenase-cyclohydrolase 1 (MTHFD1) and ubiquitination-related protein Ubiquilin 1 (UBQLN1), inducing MTHFD1 ubiquitination and degradation, aggravating the accumulation of Hcy, oxidative stress and DKD progression. Thus, these findings suggest that lncPTEC could function as an alternative diagnostic biomarker and a novel therapeutic target for DKD.

## Materials and Methods

### Cell culture

Mouse proximal tubular epithelial cells, mouse mesangial cells, and mouse podocytes were purchased from WHELAB (China) and cultured in a moist incubator (37 °C, 5% CO_2_) in our laboratory. All cell lines used were authenticated and tested negative for mycoplasma contamination. Proximal tubular epithelial and mesangial cells were grown in DMEM supplemented with 10% FBS (Gibco, USA). Podocytes were grown in RPMI 1640 medium (Gibco, USA) containing 10% FBS. Cells were subjected to either 25 mmol/L glucose (high glucose, HG) or a combination of 5.5 mmol/L glucose with 19.5 mmol/L mannitol (low glucose, LG). Biological replicates (n) refer to independently cultured and treated cells, harvested and assayed on different days. Data are presented as mean ± standard deviation (SD) from independent experiments with statistical significance.

### Animal experiments

All animal experiments were approved by the Institutional Animal Care and Use Committee of Chongqing Medical University (Approval no. 2022135), and all methods were performed in accordance with the relevant guidelines and regulations. Naturally developed DKD mice with genetic defects in the leptin receptor (db/db) are a well-established model for type 2 diabetes, obesity, and insulin resistance. When db/db mice were over 8 weeks old, their urine microalbumin and blood glucose were significantly higher than those of controls, simulating the onset process of human DKD [21]. Involved 8-week-old male db/db mice with a C57BL/BKS background (Leprdb/+Leprdb) (*n* = 35) and normal C57BL mice (*n* = 15), sourced from Chongqing Enswell Biotechnology Co, Ltd. and the Animal Center of Chongqing Medical University, China. Block randomization was used to single-blindly randomize mice into different groups of similar sample size. DKD and NC mice were utilized for scRNA-seq and in vivo lncPTEC silencing experiments.

For scRNA-seq analysis, blood glucose, urinary microalbumin (UmAlb), urinary creatinine, and urinary albumin-to-creatinine ratio (UACR) were measured in 12-week-old DKD and control mice. One month later, the mice were euthanized, and kidney samples were collected for scRNA-seq analysis. For lncPTEC knockdown experiments, lentiviruses carrying lncPTEC-shRNA were constructed and produced by TSINGKE (China). The lncPTEC primer sequences were as follows: forward 5′-ACCTCCAGCTCAATGTTATCATCAGTG-3′ and reverse 5′-AAGGGTTCTAAG AGCCGTTCATGTG-3′. Twelve-week-old DKD mice were administered tail vein injections of LV-sh-NC or LV-sh-lncPTEC lentivirus vectors at a concentration of 1 × 10^8^ TU/mL in a volume of 100 µL. Blood glucose, body weight, creatinine, and urinary microalbumin levels were monitored every two weeks, while the distribution of the lentivirus vector in the renal organ was assessed using the Wildelbard imaging system designed for small animals in Germany. Four weeks later, the mice were sacrificed. Tissue specimens were preserved either by fixation with 4% PFA or by freezing in liquid nitrogen.

### Clinical samples

Plasma specimens were obtained from DKD patients (*n* = 41) and healthy controls (*n* = 29) at the First People’s Hospital of Zunyi, and all plasma samples were stored at −80°C. Informed consent was obtained from all participants. The human study was conducted in accordance with the Declaration of Helsinki and approved by the Ethics Committee of the First People’s Hospital of Zunyi (No. 2022-120). Block randomization was used to allocate samples into different groups of similar sample size. Sample size was determined by power analysis using G*Power software (version 3.1.9.7), with the α set to 0.05 as a 5% probability of type I error occurrence and statistical power set at 0.8. Finally, the clinical information for DKD patients and normal individuals is provided in Table S1 (Supplementary information).

### Plasmids, siRNAs and cell transfection

The cDNA sequences of lncPTEC, SP1, MTHFD1 and UBQLN1 were amplified and subcloned into the pcDNA3.1 vector (TSINGKE, China), respectively, to construct overexpression plasmids. Plasmids encoding HA-tagged ubiquitin variants include wild-type ubiquitin (HA-Ub-WT) and lysine-only mutants in which all lysines except K11, K48, or K63 were substituted with arginine (HA-Ub-K11-only, HA-Ub-K48-only, and HA-Ub-K63-only, respectively). Small interfering RNAs (siRNAs) targeting lncPTEC, MTHFD1, UBQLN1, and SP1, along with their control reagents, were procured from RiboBio (China) and TSINGKE (China). The shRNA sequences designed to target lncPTEC, as well as control hairpin constructs, were inserted into the pGLVH1/GFP vector (TSINGKE, China). Cell transfection was performed with plasmids or siRNAs using Lipofectamine 2000, following the manufacturer’s protocols.

### ROS assay

Evaluation of ROS in renal tissue involves the use of dihydroethidium (DHE) staining (Sigma-Aldrich, Canada). This staining technique was applied to frozen kidney sections. In brief, frozen kidney sections were fixed in acetone for 30 min and subsequently incubated with DHE (5 μM) for another 30 min at 37°C. Images were acquired using an Olympus optical microscope.

Renal tubular epithelial cells were seeded into 96-well plates at a density of 2 × 10^4^ cells per well. After 24 hours, cells were cultured under low-serum conditions (1% FBS) for 6 hours. After 24 h treatment with different interventions, the cells were washed with PBS for 3 times. Subsequently, the medium was substituted with a serum-free solution containing 10 μmol/L DCFH-DA and incubated in the dark for half an hour. Discard the medium and wash with serum-free medium for 3 times. Finally, PBS was added, and fluorescence was measured using a microplate reader (excitation: 485 nm; emission: 525 nm).

### SOD activity assay

The SOD activity in renal tubular epithelial cells was measured following the kit instructions. The cells were lysed with RIPA buffer for total protein extraction, and protein concentration was determined using a BCA assay. Following the addition of the specified reagents, the plate was incubated at 37 °C for 20 min. Optical density measurements at 450 nm were recorded for each well using a plate reader.

### NADPH/NADP^+^ assay

The NADPH/NADP^+^ ratio in cells was evaluated using a colorimetric assay (Abcam, UK). Sorted cell populations were seeded in 96-well culture-treated plates at a density of 5 × 10^3^ cells per well. All experimental procedures were carried out in compliance with the manufacturer’s instructions.

### Mitochondrial membrane potential (MMP) assay

MMP was assessed using the cationic fluorescent dye JC-1. Cells were incubated with JC-1 working solution at 37 °C for 30 min in the dark, followed by three washes with PBS, each lasting 5 min. Then the cells were observed under a fluorescence microscope or analyzed using a fluorescence microplate reader. Under normal cellular homeostasis, JC-1 accumulates in polarized mitochondria, forming aggregates (red). During apoptotic progression, mitochondrial membrane depolarization results in JC-1 monomerization (green). The relative MMP was quantified by calculating the ratio of red to green fluorescence intensity.

### Liquid chromatography-tandem mass spectrometry (LC-MS/MS)

We conducted untargeted LC-MS/MS on plasma samples from both normal human subjects (n = 10) and DKD patients (*n* = 10) using METWARE in Wuhan, China. A TripleTOF 6600+ mass spectrometer (SCIEX, USA) coupled with an LC-30A system (Shimadzu, Japan) was used. The HPLC separation protocol was established using a Waters ACQUITY Premier HSS T3 analytical column (1.8 µm, 2.1 × 100 mm) at 40 ± 0.5 °C. A gradient elution system was prepared with two mobile phase components: (A) deionized water containing 0.1% formic acid and (B) HPLC-grade acetonitrile with 0.1% formic acid. Chromatographic conditions were maintained at a 0.4 mL/min flow rate with precise 4.0 μL injections, achieving optimal separation efficiency.

### Hcy assay

The Hcy content in renal tubular epithelial cells was quantified using the Hcy assay kit (RUIXIN BIOTECH, China) following the manufacturer’s guidelines. The samples were added to the assay kit microplate, followed by the addition of the reagents and incubation at 37 °C for 15 min in the dark. Following incubation, the stop solution was added to each well. Absorbance at 450 nm was measured using a spectrophotometer. We treated the mouse renal tubular epithelial cells under high glucose conditions with Hcy concentrations of 0, 50, 100, 200, and 500 μM.

### Single-cell RNA sequencing (scRNA-seq)

Renal tissue samples were obtained from db/db mice (DKD model group, *n* = 3) and age-matched controls (NC group, *n* = 3). Single-cell suspensions were prepared using the 10× Genomics platform for automated mRNA capture and cDNA synthesis. Sequencing libraries were constructed and analyzed using the Illumina sequencing system (Omicsmart, China). Raw sequencing data were analyzed with 10× Genomics Cell Ranger, while quality control and downstream analyses were conducted with the R package Seurat. Cells with more than 200 detected genes, fewer than 50,000 genes, and less than 25% mitochondrial gene content were retained for downstream analysis. Dimensionality reduction was visualized using t-distributed stochastic neighbor embedding (t-SNE) overlays and marker gene heatmaps. Cell clusters were determined using Seurat’s FindClusters function, which employs a K-means clustering algorithm.

### RNA extraction, cytoplasmic/nuclear fractionation and qRT-PCR

Total RNA from cells or tissue samples was extracted using TRIzol reagent (Takara, China) according to the manufacturer’s instructions. The isolated RNA was subsequently reverse-transcribed into cDNA and analyzed by quantitative real-time PCR (qRT-PCR). Cellular fractionation into cytoplasmic and nuclear components was achieved using the PARIS™ Kit (Thermo Fisher Scientific, USA), followed by RNA purification from each fraction. Reverse transcription was conducted using the PrimeScript RT Reagent Kit (Takara, China). The cDNA was amplified using TB Green Premix Ex Taq (Takara, China) on a Bio-Rad CFX96 system (Bio-Rad, USA). The mRNA expression was quantified using the 2^–ΔΔCT^ method with β-actin as internal control. All primer sequences and their validation data are provided in Supplementary Table S2.

### Immunofluorescence (IF) and fluorescence in situ hybridization (FISH)

For immunofluorescence staining, cells grown on coverslips were fixed with 4% paraformaldehyde, permeabilized with 0.1% Triton X-100, and subsequently blocked with 5% bovine serum albumin (BSA). They were then incubated with primary antibodies against MTHFD1 (A8661, ABclonal, China), Vim (A19607, ABclonal), α-SMA (A17910, ABclonal), E-Ca (A20798, ABclonal), ZO-1 (A0659, ABclonal), Col-IV (A10710, ABclonal), and Fn (A16678, ABclonal) at 4 °C overnight. The samples were then incubated with an FITC-conjugated secondary antibody for 2 hours at 37°C. Subsequently, the nuclei were stained with 4’, 6-diamidino-2-phenylindole (DAPI). Images were acquired using epifluorescence or confocal laser scanning microscopy and subsequently analyzed quantitatively using LAS AF Lite software (Leica, Germany).

A customized fluorescence in situ hybridization assay was performed with probes specifically designed to target the lncPTEC junction region according to the instructions (Genetic Pharmaceuticals, China), and subsequently counterstained with DAPI. U6 and 18S probes were used as nuclear and cytoplasmic localization controls, respectively. The samples were mounted and imaged using a confocal laser scanning microscope (Leica, Germany).

### Immunoblotting analysis

Total protein from cells or tissues was extracted using RIPA lysis buffer with PMSF, separated by SDS-PAGE, and transferred to PVDF membranes. Membranes were blocked with 5% skimmed milk and incubated overnight at 4 °C with primary antibodies targeting ZO-1, E-cadherin, Vimentin, α-SMA, Fibronectin, Collagen type IV, MTHFD1, SP1 (A19649, ABclonal), CCNF (sc-515207, SANTA), KCTD13 (sc-393994, SANTA), and β-actin (AC026, ABclonal). Subsequently, HRP-conjugated secondary antibodies (AS014, ABclonal) were applied at room temperature for 2 hours.

### RNA-seq

Differential mRNA expression between lncPTEC-overexpressing cells and control cells in the LG group was analyzed by RNA-seq using the Illumina HiSeq platform, and subsequent data analysis was performed by TSINGKE (China).

### Bioinformatics analysis methods

The secondary structure of lncPTEC was predicted using AnnoLnc2 (http://annoLnc.gao-lab.org/). The coding potential of lncPTEC was assessed using the CPAT online tool (http://lilab.research.bcm.edu/cpat/) and the UCSC Genome Browser (https://genome.ucsc.edu/). To elucidate the functional significance of lncPTEC, gene ontology (GO) and pathway enrichment analyses of transcripts altered by lncPTEC overexpression were performed using Metascape (https://metascape.org/) and KOBAS (http://kobas.cbi.pku.edu.cn). Potential transcription factors regulating lncPTEC expression were predicted using PROMO (http://acgt.cs.tau.ac.il/promo/) and LASAGNA (https://biogrid-lasagna.engr.uconn.edu/), while JASPAR (http://jaspar.genereg.net/) was used to predict binding sites of SP1 on the lncPTEC promoter. BIOGRID (http://thebiogrid.org) was used to predict potential interactions between MTHFD1 and ubiquitination-related molecules. Expression levels of UBQLN1 and MTHFD1 in diabetic kidney disease (DKD) patients and healthy controls were analyzed using the Nephroseq database (https://nephroseq.org).

### Transcription factor activation screening assay

Nucleic acids were extracted from proximal tubular epithelial cells and incubated with biotin-labeled probes targeting transcription factor-DNA binding sites, according to the manufacturer’s instructions for the Transcription Factor Activation Profiling Plate Array I (Silicon Valley, USA). Then, free probes were removed from the mixed solution through column centrifugation, and specific probes were separated from the complex for plate hybridization analysis. Finally, the activity of transcription factors in 96-well plates was detected by a microplate reader.

### Dual-luciferase reporter assay

A dual-luciferase reporter assay was conducted by subcloning the lncPTEC promoter, which includes five predicted binding sites and their respective mutants, into pGL3-Basic (TSINGKE, China) to create luciferase reporter vectors. Cells were simultaneously transfected with an SP1 overexpression plasmid, either lncPTEC-WT or lncPTEC-MUT reporter constructs, and Renilla luciferase plasmids. Following a 48-hour incubation period, luciferase activity was quantified through the Dual Luciferase Reporter Assay Kit (Hanbio, China), and normalized to Renilla luciferase activity according to the established protocol.

### Chromatin immunoprecipitation (ChIP)

ChIP assay was performed using SP1-specific antibodies, IgG control, and the SimpleChIP® Enzymatic Chromatin IP Kit (Millipore, USA) according to the manufacturer’s instructions.

### RNA pull‑down and mass spectrometry (MS)

RNA pull-down and MS assays were conducted using biotin-labeled probes targeting lncPTEC, synthesized with the MAXIscript^TM^ Kit (Thermo Fisher Scientific, USA) following the guidelines. RNA pull-down assay was performed using Pierce^TM^ Magnetic RNA-Protein Pull-down Kit (Thermo Fisher Scientific, USA) according to the manufacturer’s guidelines. Protein identification was achieved through either immunoblot detection or mass spectrometry (Novogene, China).

### RNA immunoprecipitation (RIP)

RIP was conducted using anti-MTHFD1, anti-IgG, and the EZ Magna RIP kit (Millipore, USA). The co-precipitated RNAs and MTHFD1 proteins were detected by qRT-PCR and immunoblotting, respectively.

### Coimmunoprecipitation (Co‑IP) and ubiquitination assays

Co-IP was conducted using the Pierce™ Classic Magnetic IP/Co-IP Kit (Dia-An Biotechnology, China) with antibodies against MTHFD1, UBQLN1, CCNF, KCTD13, or IgG. Antibodies were crosslinked with magnetic beads at 4 °C for 8 hours. Cells were lysed using IP lysis buffer, and the antibody-conjugated magnetic beads were incubated with the cell lysate at 4 °C for 12–16 hours. After washing 3 times, the immunoprecipitated proteins were eluted in loading buffer and analyzed by immunoblotting.

For the ubiquitination assay, cells were cotransfected with the indicated plasmids and then treated with the proteasome inhibitor MG132 at a final concentration of 10 μM for 8 hours before collection. Cell lysates were prepared using IP lysis buffer, diluted tenfold, and then subjected to sonication and centrifugation. Immunoprecipitation was performed as described for the Co-IP assay. MTHFD1 ubiquitination was analyzed by immunoblotting with anti-Ub antibodies (A19686, ABclonal) or the corresponding anti-HA tag antibody (AE105, ABclonal).

### Statistical analysis

Statistical analysis was performed by a researcher blinded to the experimental groups. Data from at least three independent experiments are presented as mean ± SD. Before parametric testing, the assumptions of normality and homogeneity of variance were confirmed using the Shapiro-Wilk test and Levene’s test, respectively. Based on these satisfied assumptions, inter-group comparisons were performed using the Student’s *t*-test or one-way ANOVA. Statistical analyses were performed using GraphPad Prism 8.0 and SPSS 22.0. *P*-value below 0.05 was deemed statistically significant.

## Results

### Mitochondrial oxidative damage mediated by Hcy accumulation is involved in DKD

**Fig. 1:**
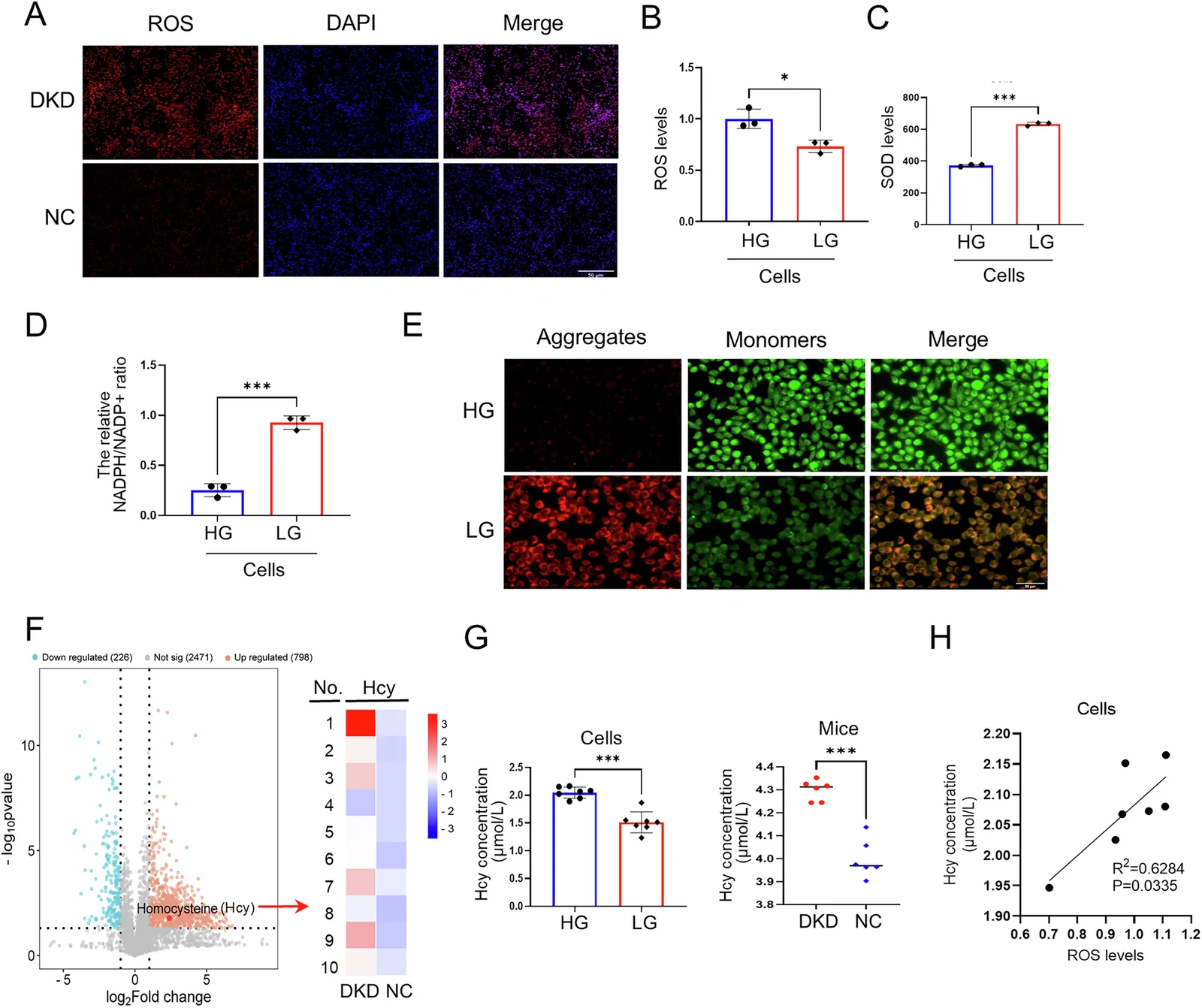
Mitochondrial oxidative damage mediated by Hcy is involved in DKD. **A** Representative images of changes in ROS levels in mice kidneys visualized by DHE (red) and nuclei (DAPI, blue) staining. The scale bar is 50 μm. **B** ROS levels, as assessed by DCFH-DA fluorescence, in PTECs in response to high glucose (HG) versus low glucose (LG) exposure. **C** SOD levels in PTECs following culture under HG or LG conditions. **D** The NADPH/NADP⁺ ratio in PTECs was altered by HG versus LG culture. **E** Representative fluorescent images showing changes in mitochondrial membrane potential (MMP), as determined by JC-1 staining, in PTECs cultured under HG or LG conditions. The scale bar is 50 μm. **F** The abnormal metabolites in DKD patients were examined by LC-MS/MS, *P* < 0.05, log2|FC | > 2. **G** Hcy levels were measured in cells of the HG and LG groups. Hcy levels in kidney tissues were measured in DKD mice group (4.30 ± 0.04 μmol/L, *n* = 6) and NC mice group (4.00 ± 0.08 μmol/L, *n* = 6) (*P* < 0.001). **H** Quantitative assessment using Pearson’s correlation metric revealed a positive association between reactive oxygen species accumulation and homocysteine levels in PTEC maintained under hyperglycemic conditions (*n* = 7). Data are shown as the mean ± SD. *P < 0.05, **P < 0.01, ***P < 0.001 by unpaired t-test (**B**–**D**, **G**).

Oxidative stress, defined as an imbalance between the production of free radicals, reactive metabolites, or ROS and their elimination, was assessed in DKD using oxidative damage markers in both in vivo and in vitro models, as well as by examining abnormal metabolites in DKD patients. Our findings indicated elevated ROS levels in both the kidney tissues of DKD mice and renal tubular epithelial cells exposed to high glucose, compared to respective control groups (Fig. 1A, B). SOD levels (Fig. 1C) and NADPH/NADP^+^ ratio (Fig. 1D) were decreased in cells cultured with high glucose. Meanwhile, a significant decrease in MMP in tubular epithelial cells cultured with high glucose was detected by JC-1 staining (Fig. 1E). Moreover, the abnormal metabolites in DKD patients were examined by LC-MS/MS. The results showed there were 1323 differential metabolites in DKD patients when compared with the normal controls, including 951 upregulated and 372 downregulated (*P* < 0.05, log2|FC | > 2) (Fig. 1F). Among these metabolites, Hcy was identified as upregulated in DKD patients. Based on prior research indicating that elevated Hcy can lead to oxidative mitochondrial damage [22], we investigated Hcy levels in DKD both in vivo and in vitro and explored the relationship between Hcy and ROS. Elevated Hcy levels were observed in both high glucose-cultured cells and kidney tissues of DKD mice (Fig. 1G). Furthermore, the content of Hcy was positively correlated with the level of ROS in cells under high glucose conditions (Fig. 1H). Together, these outcomes imply that Hcy-related oxidative mitochondrial damage occurs in DKD.

### LncPTEC is identified to be upregulated and proximal tubular cell-specific in DKD

**Fig. 2:**
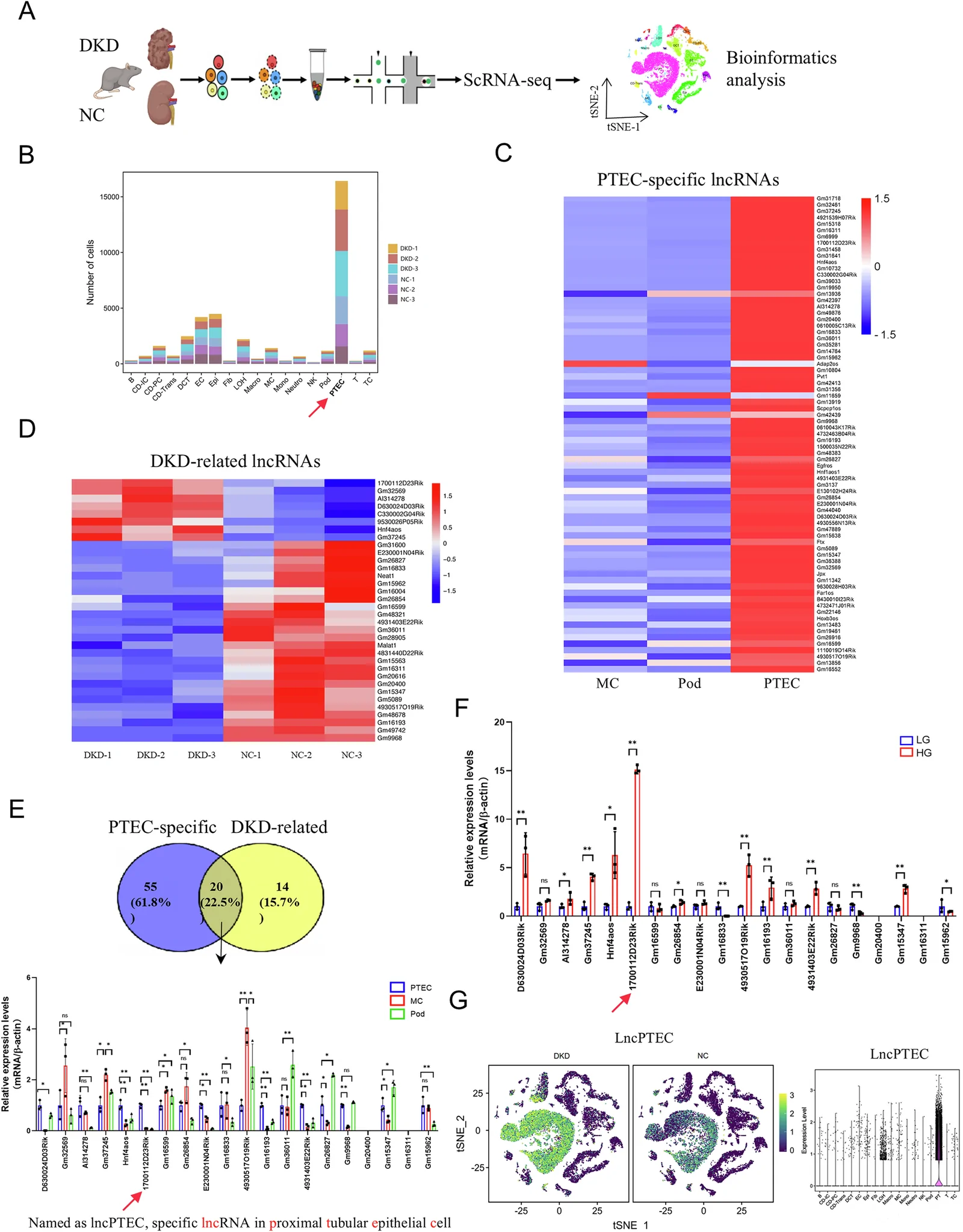
ScRNA-seq profiling of the renal tissues in DKD and NC mice. **A** Graphical summary of scRNA-seq. **B** Absolute values of the number of cells were analyzed by scRNA-seq for each cell cluster in a single cell suspension. **C** Heatmap of 75 PTEC-specific lncRNAs by scRNA-seq. **D** Analysis of the differential expression of lncRNAs in PTEC of DKD and NC groups by scRNA-seq. **E** Venny analysis of PT-specific and DKD-related lncRNAs. The transcriptional levels of 20 candidate lncRNAs in PTEC, POD, and MC were analyzed by qRT-PCR. **F** The expression of 20 candidate lncRNAs in LG-PTEC (5.5 mmol/L glucose+19.5 mmol/L mannitol) and HG-PTEC (25 mmol/L glucose) was analyzed by using qRT-PCR. **G** LncPTEC(1700112D23RIK) was highly expressed in proximal tubule cells of DKD mice by scRNA-seq. Data are presented as mean ± standard deviation, using ANOVA (**E**), unpaired t-test (**F**), with * indicating *P* < 0.05 and ** indicating *P* < 0.01, ns, not significant.

The single-cell expression profile of kidney tissues was explored in DKD and NC mice by scRNA-seq (Fig. 2A). Compared to the NC group, all db/db DKD mice exhibited DKD clinical features, including elevated blood glucose, UmAlb, UACR, and renal pathological changes such as thickened renal tubular basement membranes, increased renal tubular fibrin expression, and thickened renal tubules (Fig. S1). Furthermore, scRNA-seq results demonstrated that a total of 39098 cells were obtained, including 17837 cells from NC kidney tissues and 21261 cells from DKD kidney tissues. Through the identification of marker genes specific to each renal cell population, 18 distinct cell clusters were delineated, among which PTEC cluster was prominently featured (Fig. 2B). Furthermore, 75 lncRNAs were specific expressed in PTEC cluster compared with those in the other clusters (Fig. 2C). Meanwhile, differentially expressed PTEC lncRNAs between DKD and NC groups were analyzed, 34 lncRNAs showed significant differences, including 8 upregulated and 26 downregulated (Fig. 2D). Combined these results from PTEC-specific and DKD-related lncRNAs, we focused on 20 candidate lncRNAs, 1700112D23RIK, D630024D03Rik, Gm32569, AI314278, Gm37245, Hnf4aos, Gm16599, Gm26854, E230001N04Rik, Gm16833, 4930517O19Rik, 4931403E22Rik, Gm26827, Gm9968, Gm20400, Gm15347, Gm16311 and Gm15962. Next, the expressions of these potential lncRNAs were verified by qRT-PCR in three intrinsic renal cells (PTECs, podocytes and mesangial cells) under high glucose conditions and PTECs treated with high or low glucose. Analysis revealed that 1700112D23RIK was specifically expressed in PTECs compared with other renal cell types (Fig. 2E), and its expression was highest in PTECs cultured under high glucose conditions (Fig. 2F), which were consistent with the results of scRNA-seq (Fig. 2G). Moreover, the upregulated expression of 1700112D23Rik was found in the RNA-seq results of the DKD mouse kidney tissues among 26 differentially expressed lncRNAs (*P* < 0.05, log2|FC | > 2) (Fig. S2). As a result, we focused on lncRNA 1700112D23RIK in our study and named it lncPTEC, as the abbreviation of the specific lncRNA in proximal tubular epithelial cells.

### Enhanced lncPTEC positively correlates with proteinuria in DKD patients

**Fig. 3:**
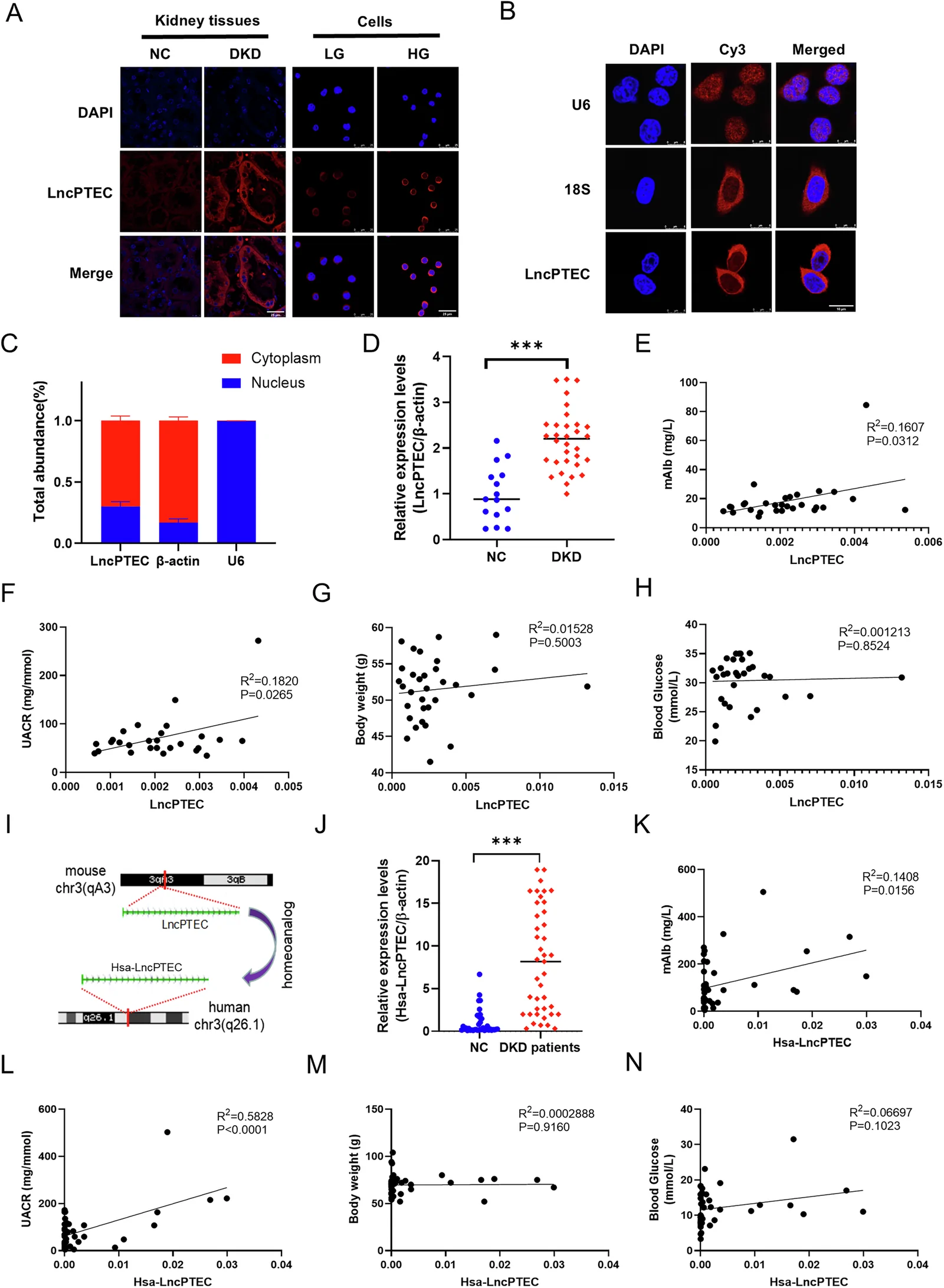
LncPTEC is markedly up-regulated in DKD and shows a positive correlation with renal function in both mice and patients. **A** LncPTEC expression was elevated in DKD both in vitro and in vivo, as demonstrated by fluorescence in situ hybridization (FISH). The scale bar is 25 µm. **B** FISH assay for lncPTEC intracellular localization in PTEC. 18S ribosomal RNA served as the endogenous cytoplasmic reference, while U6 small nuclear RNA functioned as the nuclear internal control. The scale bar is 10 µm. **C** Cytoplasmic/nuclear fractionation using qRT-PCR revealed that lncPTEC predominantly localized in the cytoplasm of PTEC, with β-actin for cytoplasmic normalization and U6 as the nuclear reference gene. **D** In db/db DKD mice (*n* = 32), LncPTEC expression in kidney tissue was greater than in the normal group (*n* = 15), as shown by qRT-PCR. **E**–**H** Pearson correlation coefficient analysis shown the high expression of lncPTEC is positively correlated with proteinuria and UACR of db/db DKD mice and negative of body weight and blood glucose (*n* = 32). **I** Location of lncPTEC and homologous analogs in human genes. **J** The plasma levels of hsa-lncPTEC in DKD patients (*n* = 41) were assessed using qRT-PCR and compared to those in healthy individuals (*n* = 29). **K**–**N** Pearson correlation coefficient analysis shown the elevated levels of LncPTEC were positively correlated with proteinuria and UACR of DKD patients, but not with body weight and blood glucose (n = 41). Data are plotted as mean ± SD, unpaired t-test (**D**, **J**), * *P* < 0.05, ** *P* < 0.01, *** *P* < 0.001.

To further explore the expression and localization in proximal tubular epithelial cells, FISH was used. The data indicated an upregulation of lncPTEC in the DKD group both in vitro and in vivo (Fig. 3A). Both nucleoplasmic FISH (Fig. 3B) and qRT-PCR (Fig. 3C) results indicated that lncPTEC predominantly localizes in the cytoplasm. In addition, the connection between increased lncPTEC expression and kidney function was examined in both DKD mice and patients. lncPTEC expression was elevated in db/db DKD mice (n = 32), compared to the NC group (Fig. 3D). Pearson’s correlation analysis revealed a positive correlation between lncPTEC expression and both UmAlb (Fig. 3E) and UACR (Fig. 3F), whereas no significant correlation was found with body weight (Fig. 3G) or blood glucose (Fig. 3H). In patients, the expression of hsa-lncRNA (human homolog for lncPTEC, Ensembl: ENST00000655926; AC061708.1, chr3:170,908,857-171,054,973) was examined using NCBI-BLAST and UCSC database (Fig. 3I). qRT-PCR analysis revealed elevated hsa-lncPTEC expression in the plasma of DKD patients (*n* = 41) relative to normal healthy individuals (n = 29) (Fig. 3J). Moreover, the expression of hsa-lncPTEC was positively related to UmAlb (Fig. 3K) and UACR (Fig. 3L) in DKD patients, while it was not correlated with admission body weight (Fig. 3M) and blood glucose (Fig. 3N). These findings indicate that lncPTEC is notably upregulated in DKD and may serve as a proteinuria marker for patients with DKD.

### LncPTEC knockdown diminishes EMT and fibrosis in DKD

**Fig. 4:**
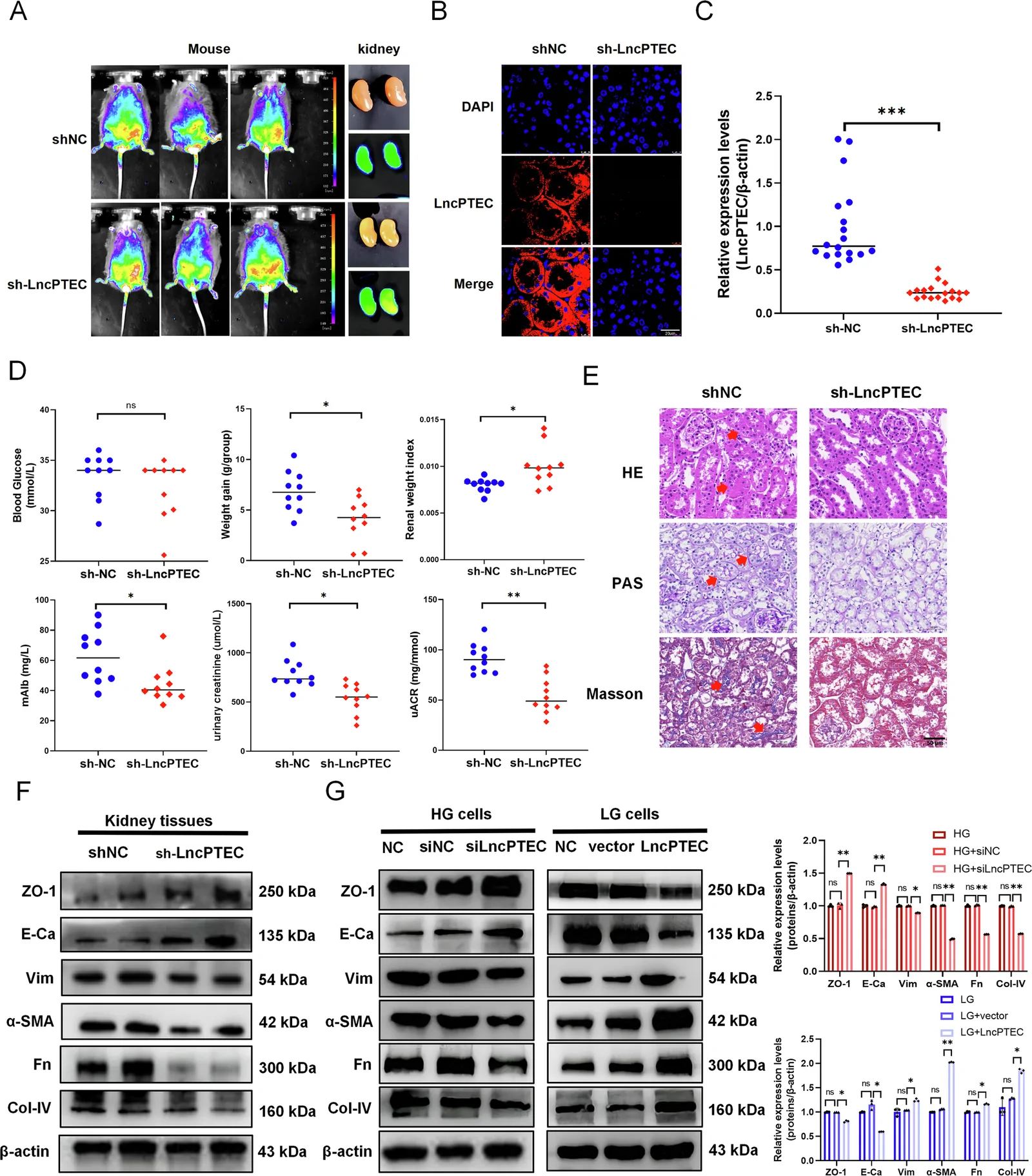
lncPTEC influences DKD progression by enhancing EMT and fibrosis both in vitro and in vivo. **A** Using mouse imaging methods analyzed how lncPTEC was distributed in the mice and their kidneys. **B** FISH analysis of lncPTEC expression in kidney tissue from mice in the sh-lncPTEC group and the sh-NC group. The scale bar is 20 μm. **C** The expression level of lncPTEC in kidney tissue was measured using qRT-PCR in both the sh-lncPTEC group (*n* = 10) and the sh-NC group (*n* = 10). **D** In both sh-lncPTEC (*n* = 10) and sh-NC (*n* = 10) groups, biochemical markers of DKD, including blood glucose, body weight, kidney weight to body weight ratio, urinary albumin, urinary creatinine, and UACR, were evaluated. **E** HE, PAS, and Masson’s staining indicated less kidney structural damage in both the sh-lncPTEC and sh-NC groups. The scale bar is 50 μm. The red arrows indicate lesions, including enlarged renal tubules, thickened basement membranes of renal tubules and interstitial fibrosis. **F** Immunoblotting analysis assesses the protein levels of ZO-1, E-ca, Vim, α-SMA, Fn, and Col-Ⅳ in kidney tissues from both the sh-lncPTEC and sh-NC groups. **G** Immunoblotting was performed to evaluate the protein expression of ZO-1, E-ca, Vim, α-SMA, Fn, and Col-Ⅳ following the transfection of lncPTEC over-expression plasmid in LG or lncPTEC siRNAs in HG. Results are presented as mean ± SD, unpaired t-test (**C**, **F**), *P < 0.05, **P < 0.01, ***P < 0.001, ns, not significant.

To examine the impact of lncPTEC on DKD progression, lentivirus-mediated lncPTEC knockdown and control groups (*n* = 10 per group) were developed using db/db DKD mice. Small animal imaging showed the lentivirus (both shNC and sh-lncPTEC) successfully reached the kidney (Fig. 4A). The renal distribution and knockdown efficacy of lncPTEC were confirmed by FISH and qRT-PCR (Fig. 4B, C). Interestingly, we found that lncPTEC knockdown ameliorated kidney function and albuminuria in DKD (Fig. 4D). HE, PAS, and Masson staining demonstrated that lncPTEC knockdown markedly alleviated DKD-related pathological changes, including enlargement of renal tubules, increased thickness of the tubular basement membrane, and the development of fibrosis in the interstitial tissue (Fig. 4E). Immunoblotting confirmed that lncPTEC knockdown increased epithelial markers E-ca and ZO-1, while reducing mesenchymal markers Vim and α-SMA, as well as fibrosis markers Col-IV and Fn in DKD mice and renal tubular epithelial cells exposed to high glucose conditions. Conversely, elevated lncPTEC reduced E-ca and ZO-1 expression and elevated Vim, α-SMA, Col-IV, and Fn expression in renal tubular epithelial cells under low glucose environments (Fig. 4F, G). On the whole, these results suggest that lncPTEC knockdown significantly reduces kidney fibrosis in DKD.

### LncPTEC increases Hcy-related oxidative stress in tubular epithelial cells

**Fig. 5:**
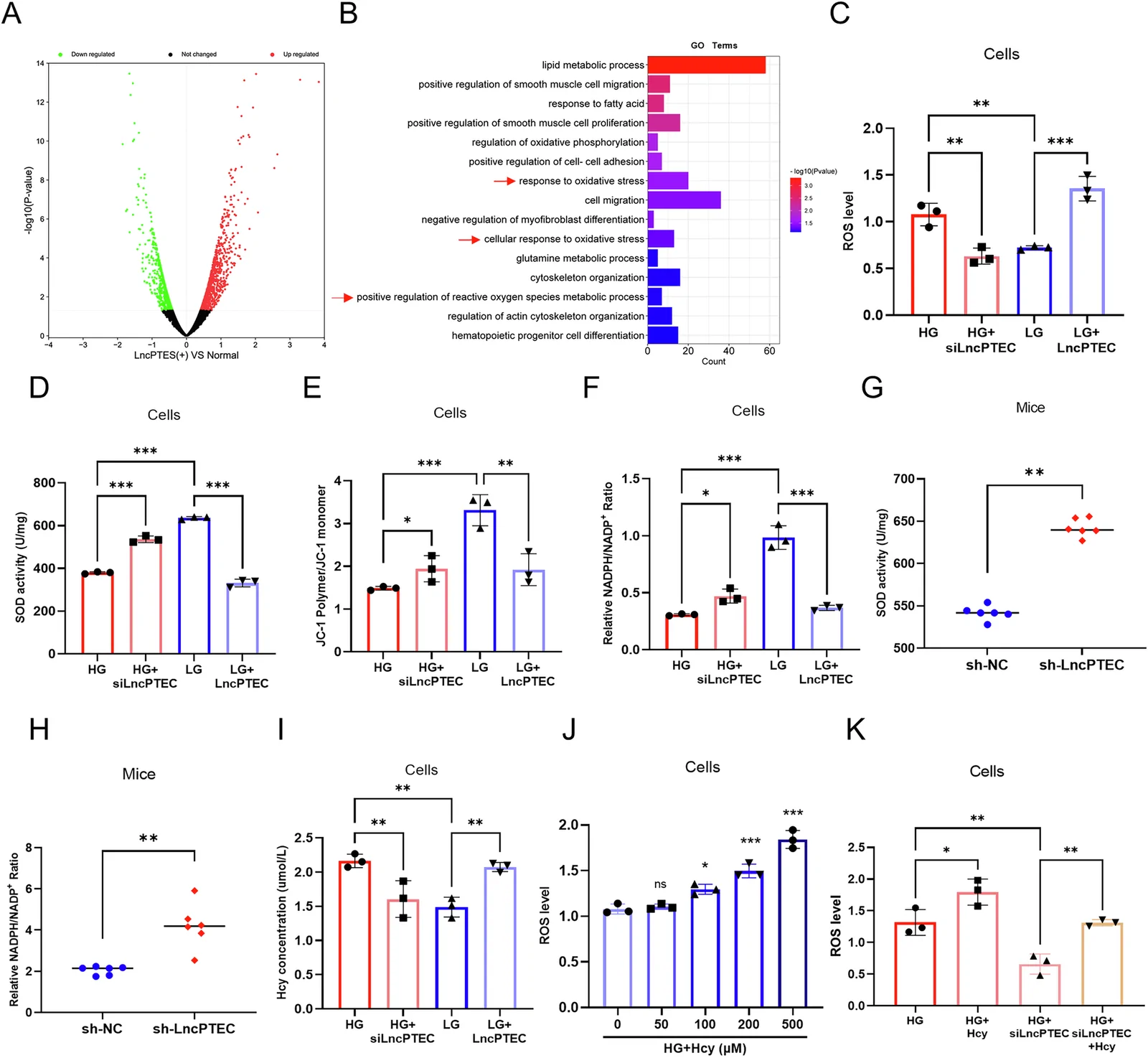
lncPTEC enhances Hcy-related oxidative stress in renal tubular epithelial cells and kidney tissues in mice with DKD. **A** RNA-seq revealed significantly differentially expressed genes between lncPTEC-overexpressing LG PTEC and control cells. **B** GO enrichment assessment of differentially expressed genes. **C** ROS levels were measured by fluorescent dye DCFH-DA with Fluorescent enzyme labeling instrument assays following the transfection of overexpressed lncPTEC under LG conditions or lncPTEC siRNAs under HG conditions. **D** SOD levels were measured following the transfection of lncPTEC overexpression in LG conditions or lncPTEC siRNAs in HG conditions. **E** MMP levels were assessed using the fluorescent dye JC-1 and a fluorescent enzyme labeling instrument following the transfection of either over-expressed lncPTEC in LG or lncPTEC siRNAs in HG. **F** NADPH/NADP^+^ ratio levels were measured after transfecting of over-expression lncPTEC in LG or lncPTEC siRNAs in HG. **G** SOD levels in kidney tissues were measured in both the sh-lncPTEC and sh-NC groups. **H** The NADPH/NADP^+^ ratio in kidney tissues was measured in both the sh-lncPTEC and sh-NC groups. **I** Hcy levels were measured after transfecting of over-expression lncPTEC in LG or lncPTEC siRNAs in HG. **J** Detection of ROS levels in high-glucose cells stimulated by different concentrations of Hcy. **K** Detection of ROS levels after adding Hcy or knocking down lncPTEC or knocking down lncPTEC followed by adding Hcy, under high glucose conditions. Results are presented as mean ± SD, unpaired t-test (**C**–**K**), *P < 0.05, **P < 0.01, ***P < 0.001.

To investigate the functional role of lncPTEC in proximal tubular epithelial cells, we conducted RNA-seq in cells overexpressing lncPTEC and corresponding controls. The study identified 1128 differentially expressed genes in the overexpressed lncPTEC group, with 679 upregulated and 449 downregulated (Fig. 5A). These genes were significantly abundant in oxidative stress-related terms, including response to oxidative stress, cellular response to oxidative stress, and positive regulation of ROS metabolic process, indicating a potential role of lncPTEC in the oxidative stress associated with DKD (Fig. 5B). We assessed oxidative mitochondrial damage in DKD both in vivo and in vitro. Hyperglycemia can induce oxidative stress [23]. Our results show that hyperglycemia significantly elevates ROS levels in renal tubular epithelial cells and silencing lncPTEC markedly reduces ROS production, while ectopic expression of lncPTEC increases ROS levels compared to controls (Fig. 5C, Fig. S4). Under high glucose conditions, renal tubular epithelial cells exhibited a reduction in SOD activity, MMP, and the NADPH/NADP^+^ ratio. Silencing lncPTEC increased these parameters, whereas ectopic lncPTEC expression reduced them under low glucose conditions (Fig. 5D–F). Furthermore, downregulation of lncPTEC was observed to elevate SOD levels and enhance the NADPH/NADP^+^ ratio in vivo, aligning with in vitro findings (Fig. 5G, H). lncPTEC knockdown decreased Hcy levels in renal tubular epithelial cells when treated with high glucose conditions, whereas increasing lncPTEC elevated Hcy levels in cells under low glucose conditions (Fig. 5I). We found that the ROS levels of high glucose cells stimulated by Hcy increased in a gradient manner with the increase of Hcy concentration (Fig. 5J). Moreover, after knockdown of lncPTEC and subsequent addition of Hcy for stimulation, the decreased ROS level was rescued (Fig. 5K). Collectively, these results suggested that lncPTEC could promote Hcy-related oxidative stress under DKD condition.

### SP1 enhances the transcription of lncPTEC in response to hyperglycemia

**Fig. 6:**
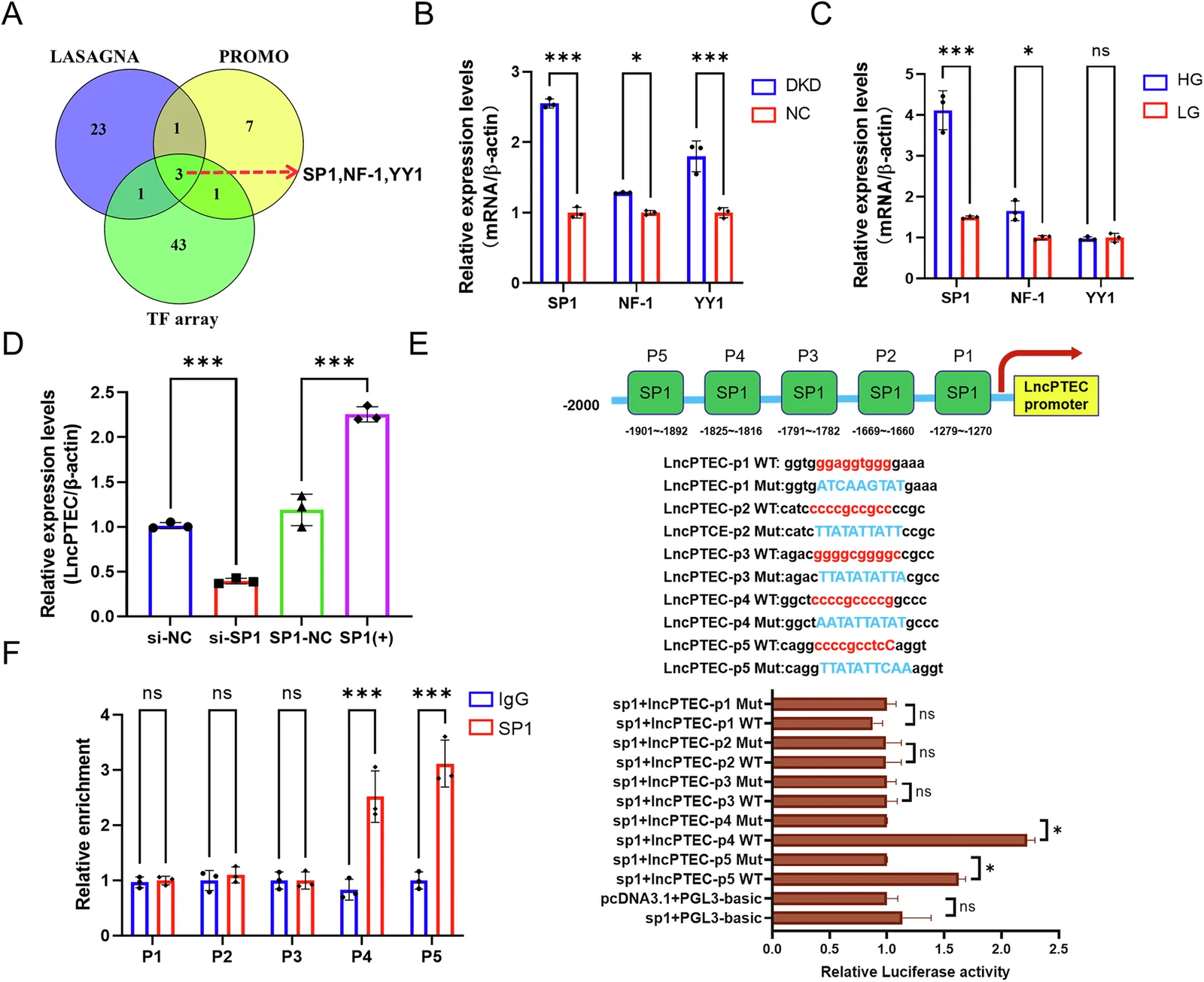
Transcription factor SP1 increases the transcription of lncPTEC in response to hyperglycemia. **A** The Venny analysis shown that 3 potential transcription factors were connected with lncPTEC. The potential transcription factors associated with lncPTEC were predicted by PROMO and IASAGNA databases. **B** qRT-PCR analysis was conducted to evaluate the expression levels of SP1, YY1, and NF-1 in kidney tissues obtained from db/db DKD mice (n = 3) and healthy control mice (*n* = 3). **C** The expression levels of SP1, YY1, and NF-1 in LG and HG cells were assayed by qRT-PCR. **D** LncPTEC levels were tested after transfecting SP1 siRNAs in HG and overexpressing SP1 plasmid in LG by qRT-PCR. **E** A schematic diagram of the luciferase reporter plasmids illustrates five predicted SP1 binding sites along with the WT and Mut sequences within the lncPTEC regulatory region. The SP1 interaction sites in the lncPTEC promoter were analyzed through dual luciferase reporter assays. **F** ChIP-qPCR assays identified the SP1 transcriptional binding motifs on the lncPTEC promoter in PTEC, using IgG as a negative control. All data are presented as mean ± standard deviation, unpaired t-test, with * indicating *P* < 0.05, ** indicating *P* < 0.01, *** indicating *P* < 0.001 and ns, not significant.

We investigated transcription factor activity in tubular epithelial cells cultured under high glucose conditions to gain insight into the upregulation of lncPTEC caused by hyperglycemia. The PROMO and LASAGNA databases were utilized to predict transcription factors targeting the lncPTEC promoter. As depicted in Fig. 6A, three transcription factors (YY1, NF-1, SP1) were found via combining the above tests. qRT-PCR analysis revealed SP1 expression was elevated in both animal models and cell culture experiments (Fig. 6B, C). Given these findings, we speculate that lncPTEC may be influenced by SP1 under high glucose conditions. To evaluate this hypothesis, we developed siRNAs and an overexpression plasmid for SP1, and their efficiency was verified at both the mRNA and protein levels (Fig. S5). Knockdown of SP1 significantly decreased lncPTEC levels under high glucose conditions, whereas SP1 overexpression increased lncPTEC levels (Fig. 6D). JASPAR was used to examine the five SP1 binding motifs in the lncPTEC promoter. Subsequently, luciferase reporter vectors were generated containing either the wild-type (WT) or mutated (Mut) lncPTEC sequences corresponding to these binding sites. Dual-luciferase reporter assays evidenced that SP1 notably enhanced the luciferase signal of lncPTEC promoters with SP1 binding sites P4 and P5, but not P1, P2, or P3 (Fig. 6E). ChIP assays confirmed the direct binding of SP1 to the P4 and P5 sites of the lncPTEC promoter under high glucose conditions (Fig. 6F). Thus, these data suggest that the transcription of lncPTEC is driven by SP1 under high glucose conditions.

### LncPTEC promotes UBQLN1-dependent MTHFD1 ubiquitination degradation via binding to MTHFD1

**Fig. 7:**
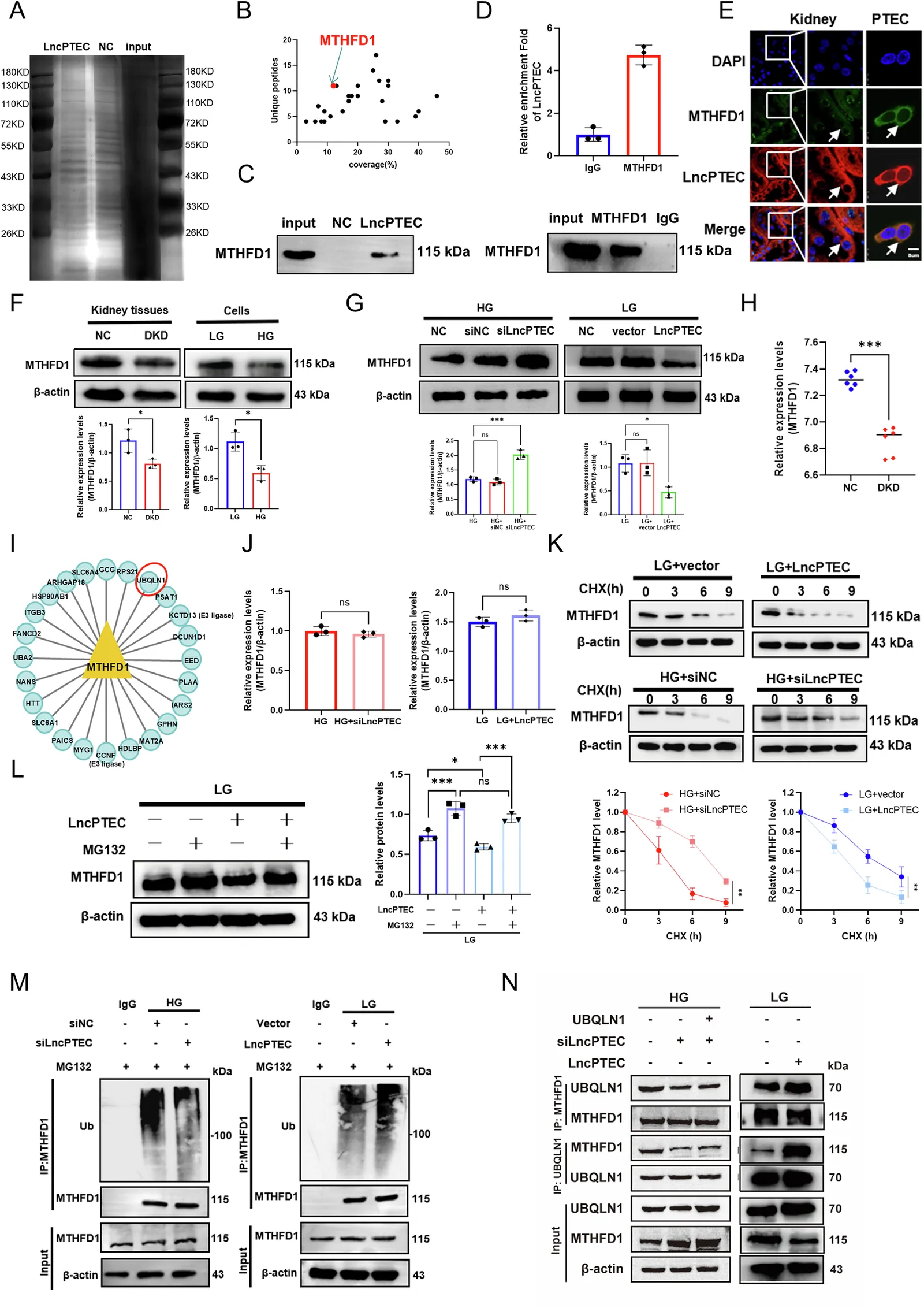
lncPTEC directly binds to MTHFD1 and promotes its ubiquitination degradation. **A** Proteins interacting with lncPTEC probes were identified using RNA pulldown followed by mass spectrometry analysis. A gel image displays the proteins captured using sense and antisense lncPTEC probes. **B** RNA pulldown followed by mass spectrometry analysis was performed to detect proteins interacting with lncPTEC probes. **C** Proteins interacting with lncPTEC probes were captured using sense and antisense RNAs, followed by immunoblotting with an anti-MTHFD1 antibody. The quantification of band intensity is shown below the blot. **D** MTHFD1 protein levels were analyzed by RNA immunoprecipitation (RIP), while lncPTEC mRNA expression was measured by qRT-PCR. **E** The localization of lncPTEC (red) and MTHFD1 (green) in the kidney tissue of db/db DKD mice and PTEC. The scale bar is 8 µm. **F** Immunoblotting was performed to detect and quantify the levels of MTHFD1 in the DKD and NC mice or HG and LG cells (*n* = 3 each group). **G** Immunoblotting was conducted to evaluate and quantify the MTHFD1 protein expression following transfection with lncPTEC over-expression plasmid in LG or lncPTEC siRNAs in HG. **H** The expression level of MTHFD1 in DKD patients in the Nephroseq database (*n* = 6 each group). **I** Potential binding proteins of MTHFD1. **J** mRNA level of MTHFD1 in lncPTEC over-expression plasmid in LG or lncPTEC siRNAs in HG by qRT-PCR. **K** Immunoblotting was conducted to evaluate and quantify the MTHFD1 protein expression following transfection with lncPTEC over-expression plasmid in LG or lncPTEC siRNAs in HG, using CHX (50 µg/mL) treatment. **L** Immunoblotting was performed to detect and quantify the expression of MTHFD1 with MG132 (10 µM) after overexpression in lncPTEC. **M** Immunoblotting analysis of the ubiquitination of MTHFD1 in lncPTEC over-expression plasmid in LG or lncPTEC siRNAs in HG and treated with 10 mM MG132 for 8 hours (Ub, ubiquitin; IP, immunoprecipitation). **N** Co-IP analysis was performed to examine the association between MTHFD1 and UBQLN1. Results are shown as mean ± SD, unpaired t-test (**D**, **F**, **G**, **I**, **L**), 2-way ANOVA (**J**, **K**) and * denoting *P* < 0.05, ** indicating *P* < 0.01, *** indicating *P* < 0.001, ns, not significant.

Based on the results above, we further investigated the function of lncPTEC in Hcy-mediated oxidative stress and the progression of DKD. Consequently, an RNA pulldown assay was conducted, followed by MS analysis. Biotin-labeled lncPTEC-specific probes identified 28 unique proteins. Strikingly, we found that MTHFD1 was pulled down by lncPTEC with 11 peptides through MS (Fig. 7A, B). It has been reported that MTHFD1, as a key enzyme in the methionine-folate cycle, can reduce Hcy accumulation [24]. Thus, these findings suggest that MTHFD1 may mediate the effects of lncPTEC on Hcy accumulation in DKD. To further validate this interaction, RNA pulldown and RIP analyses confirmed the physical association of lncPTEC with MTHFD1 (Fig. 7C, D). Moreover, FISH-IF analysis showed that lncPTEC and MTHFD1 co-localized in tubular epithelial cells, providing further evidence for their interaction (Fig. 7E). We observed reduced MTHFD1 expression in the renal tissues of DKD mice and cells exposed to high glucose by Western blot (Fig. 7F). However, lncPTEC knockdown resulted in elevated MTHFD1 levels in renal tubular cells under high glucose conditions, whereas enhancing lncPTEC expression produced the contrary effect (Fig. 7G, Fig. S6). We also observed the reduction of MTHFD1 in the renal tissues of DKD patients in the Nephroseq database (Fig. 7H). Thus, these findings suggest lncPTEC inhibits the expression of MTHFD1 via directly interacting with MTHFD1.

To determine the mechanism underlying MTHFD1 degradation, we investigated the ubiquitination of MTHFD1 based on the potential interacting proteins identified using the BIOGRID database. We identified Ubiquilin1 (UBQLN1), an ubiquitination related protein acting as ubiquitination organizer recruiting various E3 ubiquitin ligases to promote substrate ubiquitination and the proteasomal shuttle delivering the ubiquitinated proteins to the proteasome for degradation [25], as a potential binding partner for MTHFD1 (Fig. 7I). We examined the expression of UBQLN1 in human and mouse kidney tissues using the Nephroseq database and db/db mice, respectively. We found an increase in DKD (Fig. S7). When UBQLN1 was knocked down under high glucose conditions, it was observed that the levels of ROS and Hcy decreased (Fig. S8), suggesting UBQLN1-dependent MTHFD1 ubiquitination may participate in DKD. In order to further explore the specific mechanism, we conducted experiments at the transcriptional and protein levels. We found no significant difference in mRNA level of MTHFD1 after lncPTEC knockdown or overexpression (Fig. 7J). Moreover, MTHFD1 protein stability markedly increased after lncPTEC knockdown, as demonstrated by CHX treatment (Fig. 7K). We employed the proteasome inhibitor MG132 to prevent MTHFD1 degradation. By immunoblotting analysis, MG132 significantly restored MTHFD1 levels when overexpressing lncPTEC (Fig. 7L), indicating that MG132 prevented MTHFD1 degradation. To further verify this, we evaluated the polyubiquitination of MTHFD1 in cells with the overexpression of lncPTEC or lncPTEC knockdown. As expected, MTHFD1 ubiquitination was significantly increased when overexpressing lncPTEC and was decreased when silencing lncPTEC (Fig. 7M). To investigate whether the ubiquitination level of MTHFD1 is associated with disease progression, we examined its relationship with fibrotic markers Fn and α-SMA in control and DKD mice. Western blot analysis showed that MTHFD1 ubiquitination increased in the DKD mice compared with controls (Fig. S9A). Meanwhile, both Fn and α-SMA exhibited corresponding changes (Fig. S9B). Spearman correlation analysis further suggested a potential association between MTHFD1 ubiquitination levels and the expression of Fn or α-SMA (Fig. S9C). These findings imply that dysregulation of MTHFD1 ubiquitination may be linked to renal fibrosis in DKD. UBQLN1, belonging to the conserved ubiquitin-like protein family, acts as a delivery protein by targeting specific proteins for ubiquitination and facilitating their degradation via the proteasome. We observed that lncPTEC overexpression markedly enhanced the interaction between MTHFD1 and UBQLN1, whereas lncPTEC silencing decreased their binding, which could not be restored by UBQLN1 overexpression (Fig. 7N). Therefore, these suggest lncPTEC may promote MTHFD1 protein degradation via binding to MTHFD1. Since the E3 ligases recruited by UBQLN1 are the key mediators in the ubiquitination of MTHFD1, we further identified E3 ligases involved in the process of MTHFD1 ubiquitination promoted by lncPTEC. Based on the bioinformatics analysis via the BIOGRID database, two E3 ligases, CCNF and KCTD13, were found to potentially interact with MTHFD1 (Fig. 7I). Furthermore, Co-IP results verified that KCTD13, but not CCNF, was the key E3 ligase (Fig. S10). Overexpression of KCTD13 increased MTHFD1 ubiquitination and knockdown of lncPTEC partly attenuated this effect (Fig. S11). Subsequently, we evaluated KCTD13 expression in DKD patients using the Nephroseq database. Compared with normal individuals, KCTD13 was significantly elevated in DKD patients (Fig. S12A), which was consistent with the results of qRT-PCR experiments on mouse kidney tissues (Fig. S12B) and renal tubular epithelial cells (Fig. S12C). Therefore, these data suggest that the recruitment of E3 ligase KCTD13 via lncPTEC/UBQLN1 may play a key role in MTHFD1 ubiquitination in DKD. Given that lncPTEC promoted MTHFD1 ubiquitination, we sought to determine the type of ubiquitin chain that lncPTEC modifies on the MTHFD1 protein. Among the ubiquitination modifications, the two most common ubiquitin modes are K11-, K48-, and K63-linked polyubiquitination [26]. Therefore, renal tubular epithelial cells under high-glucose conditions were transfected with HA-tagged K11-only, K48-only, or K63-only ubiquitin mutants, together with or without silncPTEC. We found that MTHFD1 ubiquitination was detected only in cells expressing K48-only ubiquitin, whereas silencing lncPTEC inhibited K48-linked polyubiquitination of MTHFD1 (Fig. S13).

### MTHFD1 mediates mitochondrial damage through Hcy accumulation

**Fig. 8:**
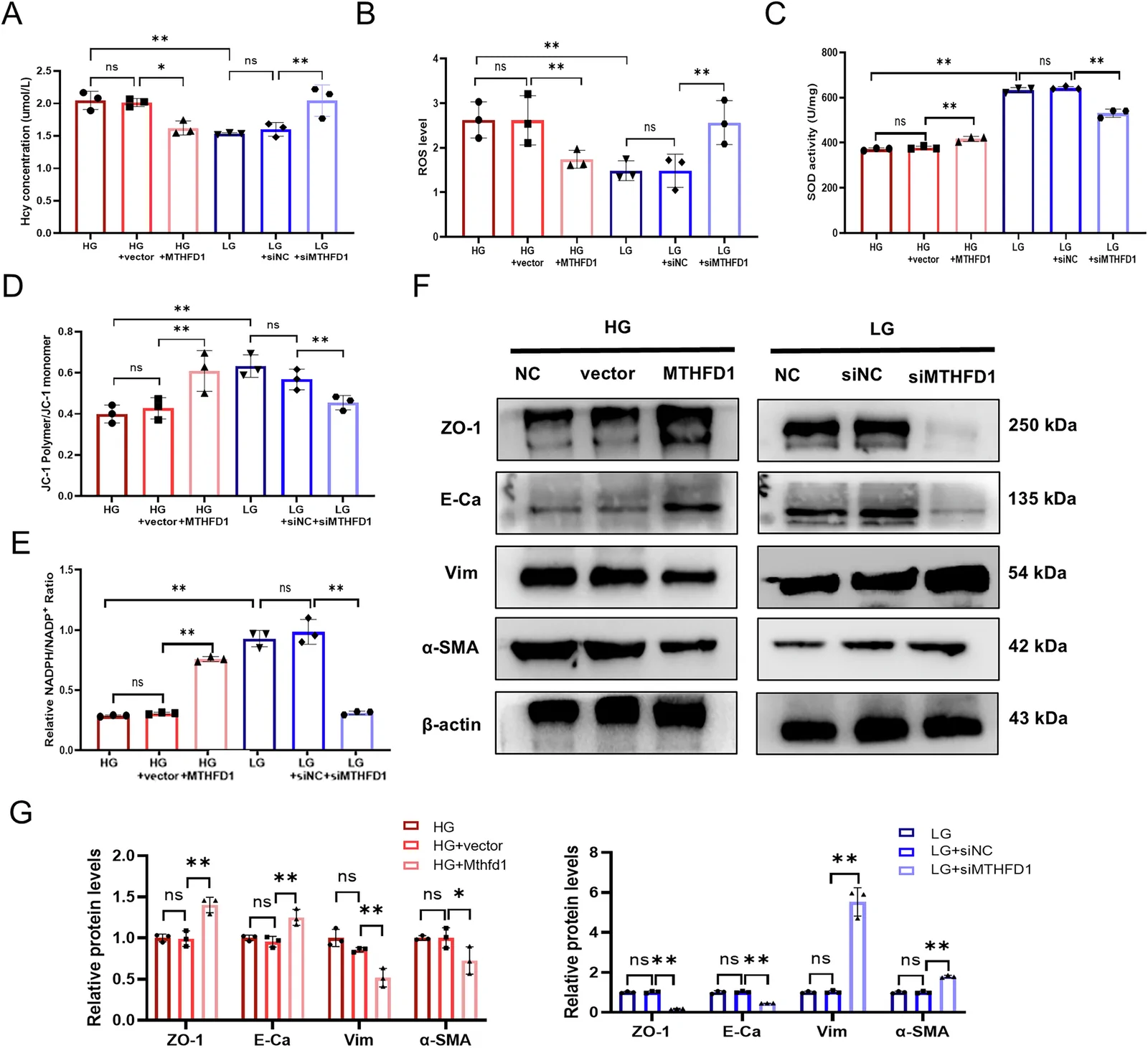
MTHFD1 regulates Hcy accumulation and mitochondrial damage in DKD. MTHFD1 was overexpressed in HG cells or knocked down via siRNA in LG cells. **A** Hcy levels were measured. **B** ROS levels were detected using DCFH-DA fluorescent dye with a fluorescence microplate reader. **C** SOD levels were measured. **D** MMP levels were assessed by fluorescence microplate reader assays. **E** The NADPH/NADP⁺ ratio was measured. **F** Protein expression of ZO-1, E-cadherin, Vimentin, and α-SMA was evaluated. **G** Quantitative analysis of Western blot data was conducted. Results are presented as mean ± SD. Statistical significance was assessed with unpaired t-tests (**A**–**F**) and ANOVA (**G**), with * *P* < 0.05, ** *P* < 0.01, and ns, not significant.

MTHFD1, a crucial enzyme in the folate metabolism process, contributes to the conversion of Hcy into methionine. We wondered whether MTHFD1 participates in the oxidative stress associated with DKD. Therefore, we conducted MTHFD1 overexpression plasmid or siRNA si-MTHFD1 to further investigate its function in DKD. Then, Hcy concentration was detected in the cells with MTHFD overexpression or MTHFD1 knockdown. MTHFD1 overexpression decreased Hcy levels in renal tubular epithelial cells under high glucose conditions, whereas MTHFD1 knockdown elevated Hcy levels in cells under low glucose conditions (Fig. 8A). Furthermore, MTHFD1 overexpression decreased ROS levels and increased SOD activity, mitochondrial membrane potential, and the NADPH/NADP^+^ ratio under high glucose conditions. Conversely, MTHFD1 knockdown in low glucose conditions elevated ROS levels and reduced SOD activity, mitochondrial membrane potential, and the NADPH/NADP^+^ ratio (Fig. 8B–E). Immunoblotting analysis revealed that MTHFD1 overexpression decreased EMT and fibrosis marker proteins in high glucose conditions, while MTHFD1 knockdown produced contrary effects in low glucose conditions (Fig. 8F, G). These findings suggest that MTHFD1 mitigates oxidative stress in renal tubular epithelial cells via Hcy metabolism and reduces EMT and fibrosis in DKD.

### Lnc contributes to oxidative stress and fibrosis by suppressing MTHFD1 in renal tubular epithelial cells

**Fig. 9:**
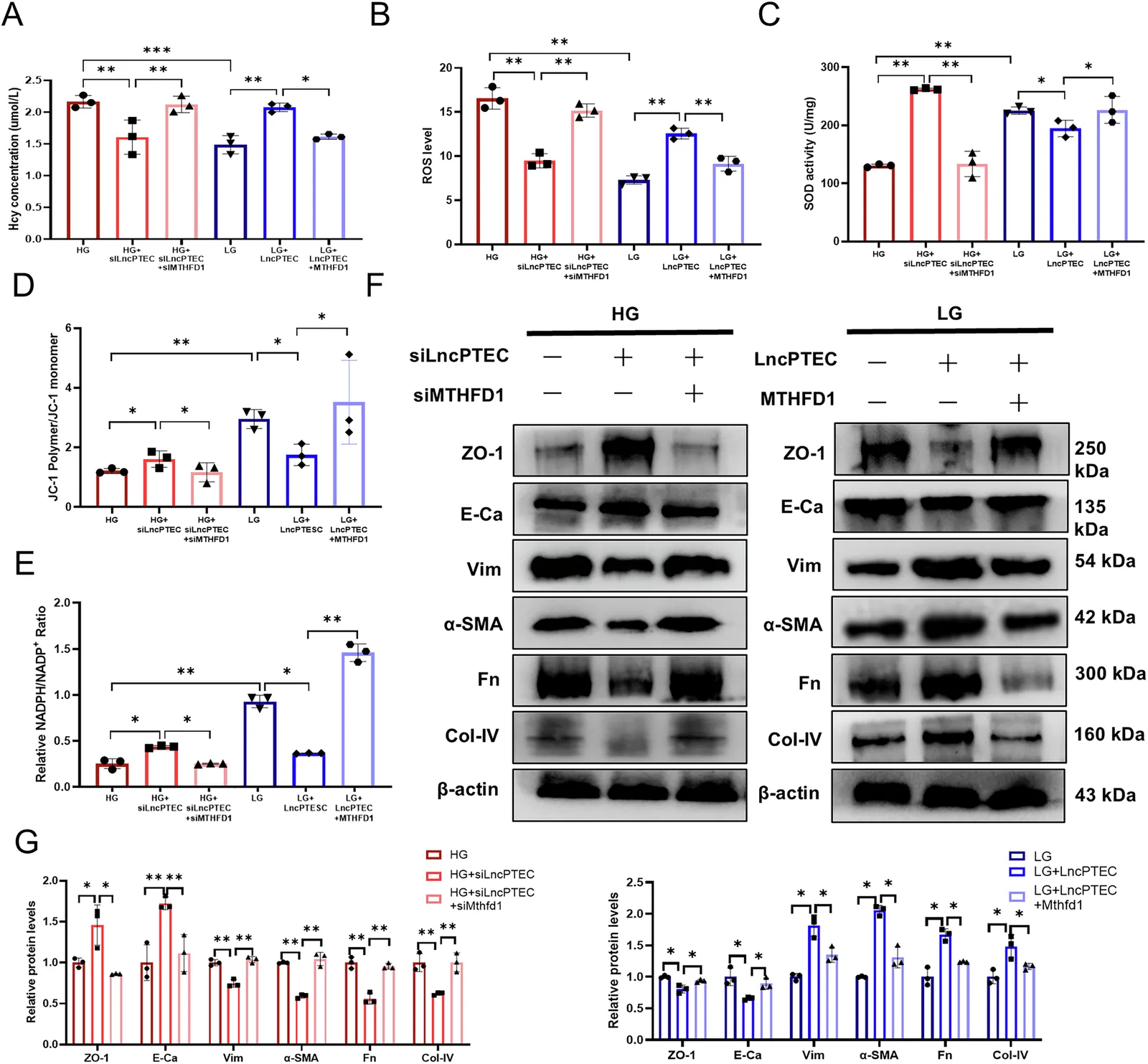
lncPTEC regulation of Hcy-mediated EMT and fibrosis by MTHFD1. Cells were transfected with the lncPTEC vector alone or co-transfected with both lncPTEC and MTHFD1 vectors. **A** Hcy levels were measured. **B** ROS levels were detected using DCFH-DA fluorescent dye with a fluorescence microplate reader. **C** SOD levels were measured. **D** MMP levels were assessed by fluorescence microplate reader assays. **E** The NADPH/NADP⁺ ratio was measured. **F** Protein expression of ZO-1, E-cadherin, Vimentin, α-SMA, Fibronectin, and Collagen IV was evaluated. **G** Quantitative analysis of Western blot data was performed. The results are represented as mean ± SD. Statistical analysis was conducted using ANOVA, with *P < 0.05 and **P < 0.01.

We explored the functional relationship between lncPTEC and MTHFD1 in renal tubular epithelial cells subjected to elevated glucose environments. Overexpression of MTHFD1 in these cells neutralized the elevated Hcy and ROS levels caused by ectopic lncPTEC, whereas MTHFD1 depletion reversed the reduced Hcy and ROS levels resulting from lncPTEC knockdown (Fig. 9A, B). Moreover, the effects of ectopic lncPTEC on decreased SOD activity, mitochondrial membrane potential and NADPH/NAPD+ ratio were rescued by overexpression of MTHFD1; while the effects of lncPTEC knockdown on increased SOD activity, mitochondrial membrane potential and NADPH/NAPD^+^ ratio were rescued by downexpression of MTHFD1 (Fig. 9C–E). Additionally, the impact of lncPTEC overexpression on elevated EMT and fibrosis marker expression was mitigated by MTHFD1 upregulation in cells exposed to high glucose environments. In contrast, the above effects of lncPTEC knockdown could be rescued by downexpression of MTHFD1 in cells under low glucose condition (Fig. 9F, G). In conclusion, the findings indicate that lncPTEC enhances oxidative stress, EMT, and fibrosis processes in renal tubular epithelial cells caused by Hcy by suppressing MTHFD1.

## Discussion

Renal fibrosis is a crucial pathological characteristic of DKD and a major factor in the progression to end-stage renal disease [27]. The primary causes of renal interstitial fibrosis-affected DKD progression include abnormal profibrotic factor expression, myofibroblast activation, and EMT [28]. A study found that activated myofibroblasts originating from epithelial cells significantly decreased E-cadherin expression and initiated fibrosis by expressing α-SMA and adopting a mesenchymal phenotype characterized by the secretion of fibrotic collagen [29]. Renal tubular epithelial cells undergo EMT, losing their reabsorption and secretory functions, which leads to the destruction of renal parenchymal structure and promotes renal fibrosis [30, 31]. The ultimate result of DKD is renal fibrosis, which remains a challenging issue in clinical practice with no proven cure. Therefore, exploring the developmental mechanisms of renal interstitial fibrosis in DKD could facilitate the identification of novel diagnostic indicators or potential therapeutic strategies [32]. Our study identified that lncPTEC enhances mitochondrial oxidative stress, promotes EMT, and drives fibrotic processes in renal tubular epithelial cells, contributing to renal injury both in vitro and in vivo, offering a novel insight into DKD research.

LncRNA has been shown to have the potential as a clinical biomarker and possible treatment target involved in the pathological damage process of DKD [33, 34]. According to a study, lncRNA PVT1 was significantly overproduced in DKD patients’ serum, and its level of expression was linked to different DKD stages and had a positive correlation with KIM-1 and NGAL [35]. In DKD patients, reduced proteinuria and renal function were positively connected with downregulated LINC01619 expression [36]. In this work, we identified renal tubular epithelial cell-specific lncRNA lncPTEC in DKD from scRNA-seq screening and discovered the hsa-lncPTEC of lncPTEC. Both lncPTEC and hsa-lncPTEC were up-regulated in DKD mice and patients, and Pearson correlation analysis showed their positive association with UmAlb and UACR, without significant correlation with body weight and blood glucose, suggesting the effect of lncPTEC may be more on the kidney than the whole body system. The above results preliminarily suggest that hsa-lnPTEC could potentially act as a biomarker for DKD. However, our results of lncPTEC knockdown in db/db mice showed that body weight was significantly decreased. It seems to contradict our finding that there was no correlation between lncRNA expression levels and body weight in DKD patients and mice, suggesting that lncPTEC may have the potential to regulate body weight on its own, but its function might be masked or compensated under pathological conditions. Future investigations with an increased sample size are required to substantiate this effect.

The scientific community widely acknowledges the crucial role of transcription factors in regulating lncRNA dysfunction [37, 38]. A prior whole transcriptome analysis of DKD mice revealed abnormal expression of certain transcription factors during DKD progression [39]. We investigated transcription factors involved in lncRNA dysregulation to understand the regulatory mechanism behind abnormal lncPTEC expression in DKD. Combined with the results of transcription factors array and bioinformatics analyses, transcription factor SP1 was found to be active in DKD and contained putative CG-rich motifs in the lncPTEC promoter. Sp1, a prominent member of the Sp/KLF family, is integral to several biological processes. Specifically, it exerts significant influence over cell growth, differentiation, and apoptosis [40]. Liu X demonstrated that PGAM5 exacerbates mitochondrial fission and the progression of diabetic renal tubular injury, with activation by SP1 [41]. Our findings indicate that high glucose levels induce SP1 expression in renal tubular cells associated with DKD. SP1 overexpression elevated lncPTEC levels, whereas SP1 downregulation decreased lncPTEC expression. Luciferase reporter and ChIP assays demonstrated that SP1 binds to the lncPTEC promoter, suggesting that SP1 transcriptionally activates lncPTEC during DKD progression.

Oxidative stress is a hallmark pathophysiological feature of DKD and is a result of an imbalance between pro-oxidant substances and antioxidant systems [42]. Hcy exacerbates imbalanced homeostasis and cellular redox, leading to significant oxidative stress [43]. Based on RNA-seq data and GO functional assessment, a significant upregulation was observed in the redox-related signal transduction cascade within cells overexpressing lncPTEC. In tubular epithelial cells, lncPTEC knockdown under high glucose conditions significantly decreased Hcy concentration, while its overexpression under low glucose conditions significantly increased Hcy concentration. This suggests that lncPTEC may influence Hcy-related oxidative stress in renal tubular epithelial cells. Interestingly, we discovered that silencing lncPTEC successfully normalized ROS but did not fully reverse mitochondrial dysfunction, which underscores the severe and potentially lasting damaging effects of prior Hcy exposure on mitochondrial enzymes, membrane integrity, and metabolic redox balance. While lncRNAs are known to regulate genes through interactions with DNA, RNA, or proteins [44], recent findings highlight their significant role as regulators by directly targeting proteins [45]. This study uncovers a novel mechanism where lncPTEC physically interacts with MTHFD1, facilitating its ubiquitination and subsequent degradation. MTHFD1 is a crucial enzyme in the folate cycle that regulates redox homeostasis in various diseases. Folate has a decisive auxiliary role in Hcy metabolism. Disorders of folate metabolism can lead to the methionine cycle, making Hcy metabolism and increasing concentration [46]. Field MS et al. reported that a mouse model of MTHFD1 insufficiency showed increased Hcy levels and decreased methionine and cystathionine, indicating impaired Hcy regulation [47]. The MTHFD1-rs2236225 missense variant results in a protein that is heat-refractory, with decreased half-life and synthetase activity [48]. This variant leads to decreased 5, 10-methylene THF formation, which is essential for Hcy regulation and may negatively impact this process [24]. Our research demonstrated that MTHFD1 knockdown in kidney tubular epithelial cells when glucose levels are low led to Hcy accumulation. Conversely, in high glucose conditions, although lncPTEC knockdown initially reduced Hcy levels, subsequent MTHFD1 knockdown resulted in Hcy accumulation once more, causing mitochondrial oxidative stress. This implies that MTHFD1 could be essential in the oxidative stress caused by Hcy in DKD.

The ubiquitin-proteasome system is a promising therapeutic target for human diseases, with ubiquitination serving as a typical degradation pathway for MTHFD1. Studies have shown that the Arsenic enhances the small-ubiquitin-like modification of MTHFD1, and the subsequent proteolytic degradation of MTHFD1 and serine hydroxymethyltransferases [49]. Rao et al. identified that the G1958A SNP in MTHFD1, which encodes the R653Q variant, is linked to reduced protein stability due to ubiquitination-mediated degradation [50]. To delve deeper into the underlying mechanism of MTHFD1’s function, we predicted the binding proteins of MTHFD1 through the BIOGRID online website and found that UBQLN1 has a high binding score. UBQLN1, part of the UBQLN family, functions as an important factor involved in protein degradation in both the recruitment of E3 ligases and the delivery of ubiquitinated proteins to the proteasome [51]. We identified that the E3 ligase KCTD13 could interact with MTHFD1 through BIOGRID database and Co-IP. KCTD13, also known as BACURD1 or POLDIP1, interacts with cullin3 (CUL3) through its N-terminal BTB domain, while the C-terminal binds and recruits substrate proteins to the E3 ubiquitin ligase complex for ubiquitination [52, 53]. Our results showed that after knocking down lncPTEC, the interaction between KCTD13 and MTHFD1 decreased. Furthermore, overexpression of KCTD13 enhanced MTHFD1 ubiquitination, and knockdown of lncPTEC partly attenuated this effect. Also, the level of KCTD13 was significantly elevated in DKD patients, mice and the renal tubular cells cultured with high glucose, suggesting the recruitment of E3 ligase KCTD13 via lncPTEC/UBQLN1 plays key roles in MTHFD1 ubiquitination in DKD. Our findings also indicate that both overexpression and silencing of lncPTEC significantly enhance MTHFD1’s binding to UBQLN1. The silence of UBQLN1 can help restore the level of MTHFD1. Thus, this suggests that lncPTEC may promote the binding of MTHFD1 to UBQLN1, which leading to ubiquitination degradation of MTHFD1. In addition, K48-linked ubiquitin chains are the canonical signal for proteasomal degradation [54]. Moreover, the E3 ubiquitin ligase KCTD13 we found in this study was reported to be associated with mediating K48-linked ubiquitination [52]. And our results show that silncPTEC inhibited K48-linked polyubiquitination of MTHFD1 protein. This reveals the crucial role of K48 ubiquitination in the degradation of MTHFD1. Furthermore, Spearman correlation analysis suggested a potential association between MTHFD1 ubiquitination levels and the expression of Fn or α-SMA, suggesting that dysregulation of MTHFD1 ubiquitination may be linked to renal fibrosis in DKD. However, further assays in DKD patient tissue samples and clinical pathological indicators are urgently needed to validate the correlation above, which will significantly enhance the translational medical value of this discovery.

In summary, our findings demonstrate that lncPTEC exhibits elevated expression in renal tubular epithelial cells across DKD in vivo and in vitro experimental systems. Additionally, the expression levels of lncPTEC and its human homolog are positively associated with increased albuminuria in DKD mice and patients. Under hyperglycemic conditions, SP1 transcriptionally upregulated lncPTEC, which in turn enhanced mitochondrial oxidative stress, EMT, and fibrosis in kidney tubular epithelial cells both in vivo and in vitro. Further mechanistic investigations demonstrated that lncPTEC has the ability to directly attach to MTHFD1 to enhance ubiquitination-related protein UBQLN1-dependent MTHFD1 ubiquitination degradation, and activate Hcy-related oxidative stress and DKD progression. Our findings indicate that hyperglycemia contributes to DKD development through lncPTEC, suggesting that lncPTEC could serve as a promising diagnostic indicator and potential intervention target for DKD.

## Supplementary information

Supplementary information

Original Western blots

## Data Availability

The data that support the findings of this study are available on request from the corresponding author, upon reasonable request.
