# Supplementary material for: LncPTEC mediated homocysteine accumulation elevates oxidative stress via UBQLN1-dependent MTHFD1 ubiquitination in DKD
Source: Cell Death Dis. 2026 Jul 7;17(1):842. doi: 10.1038/s41419-026-09093-2 (PMC13619569; doi:10.1038/s41419-026-09093-2)

Fig4F

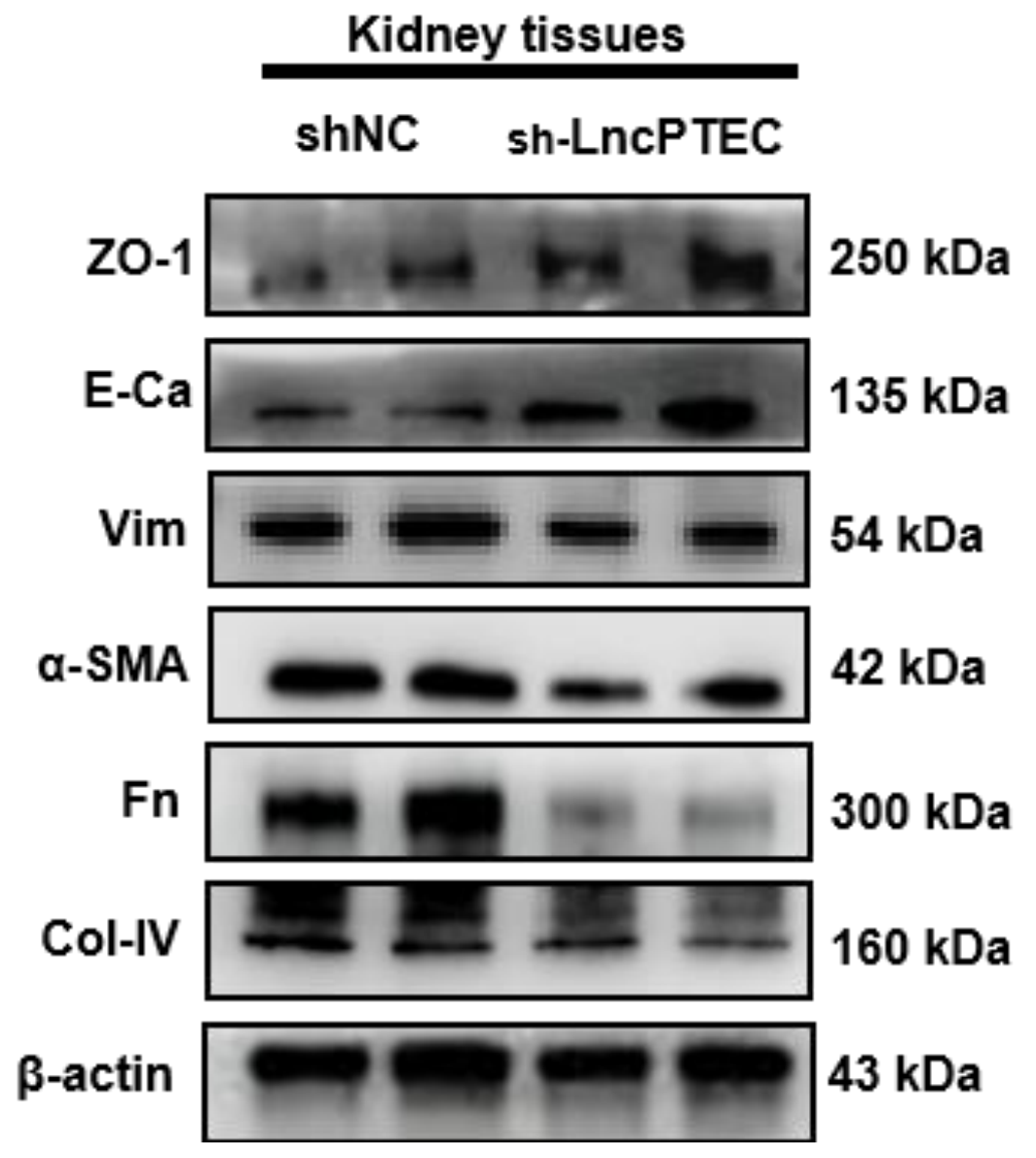

Original unprocessed gel

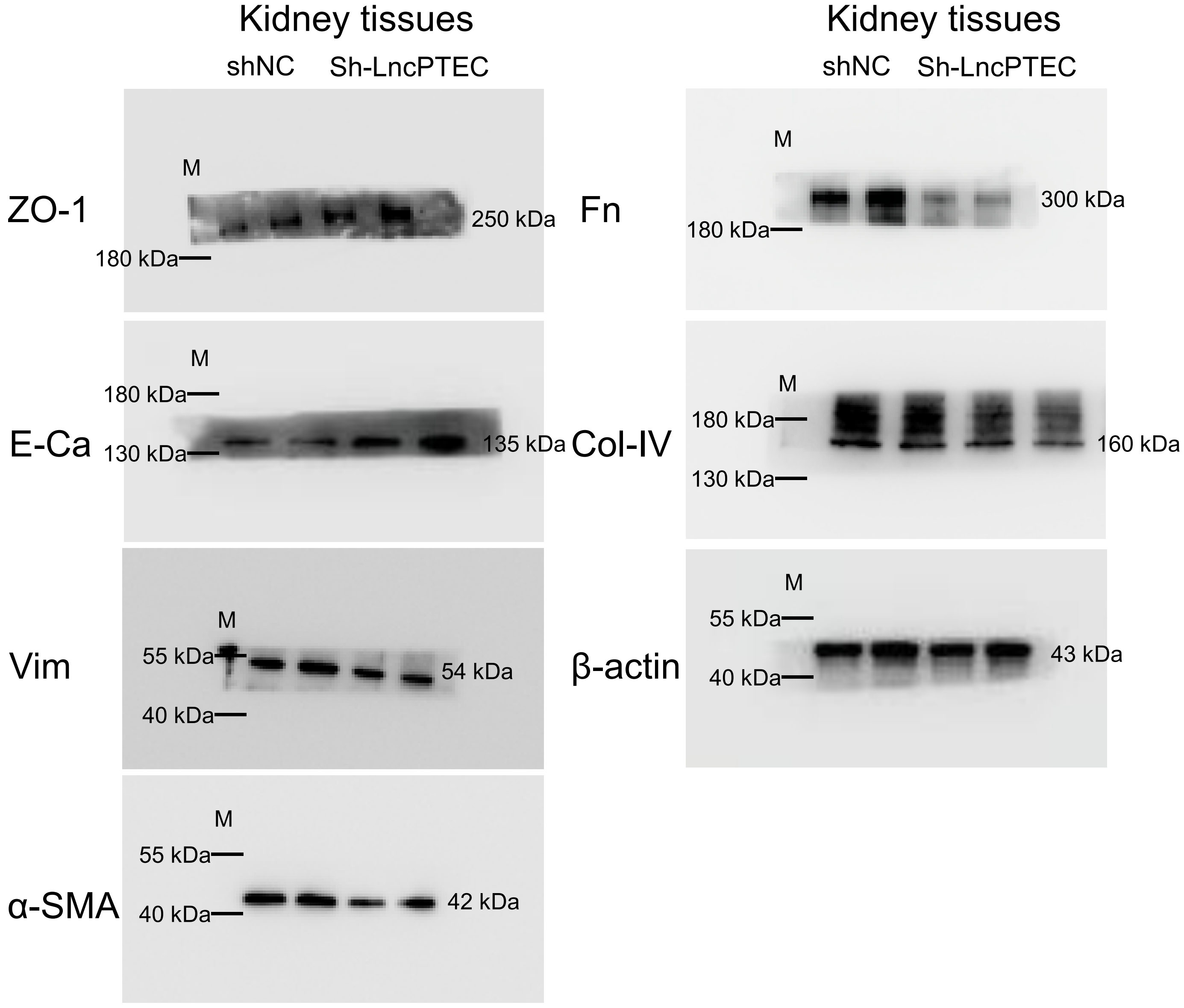

Fig4G
HG cells

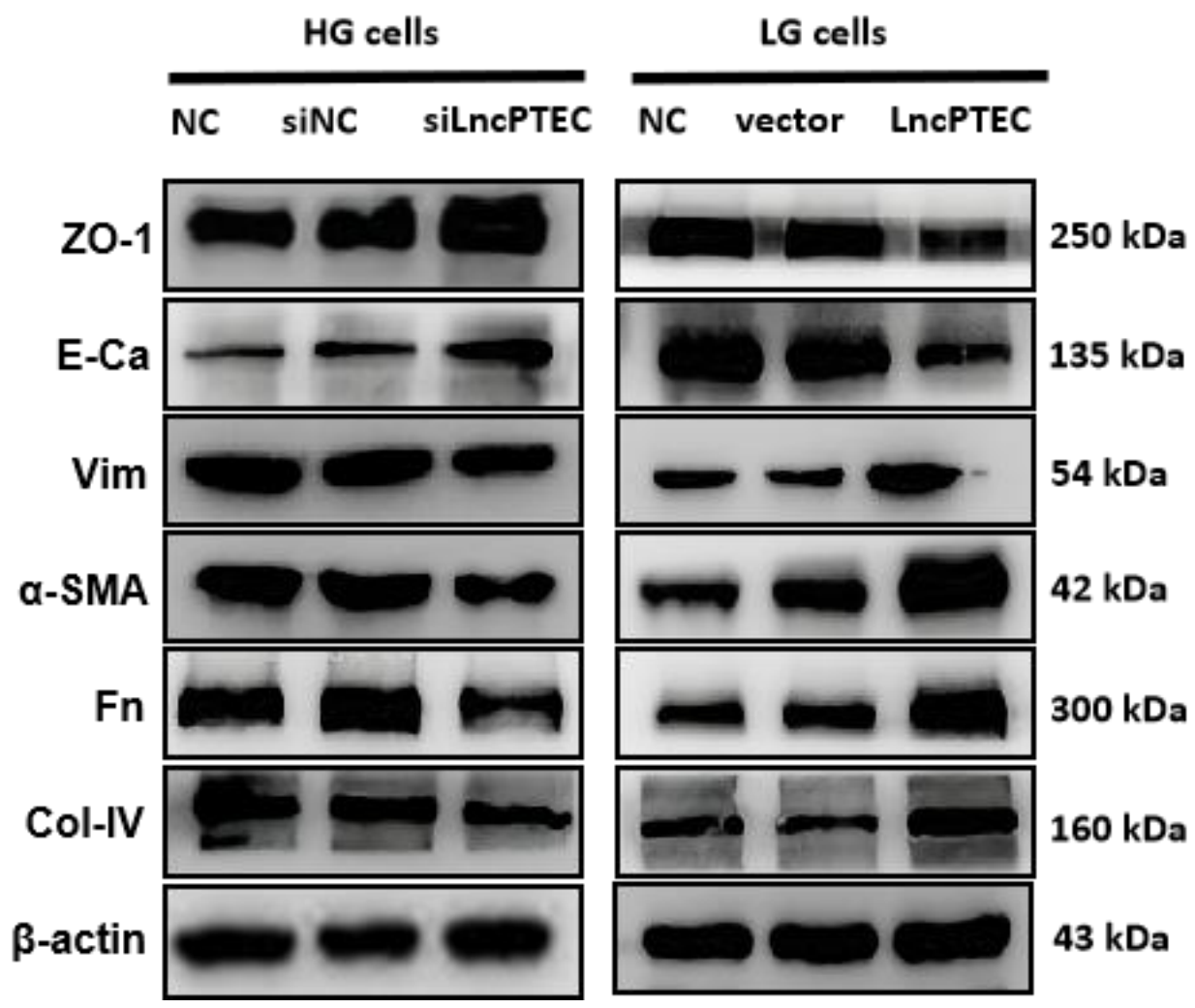

Original unprocessed gel

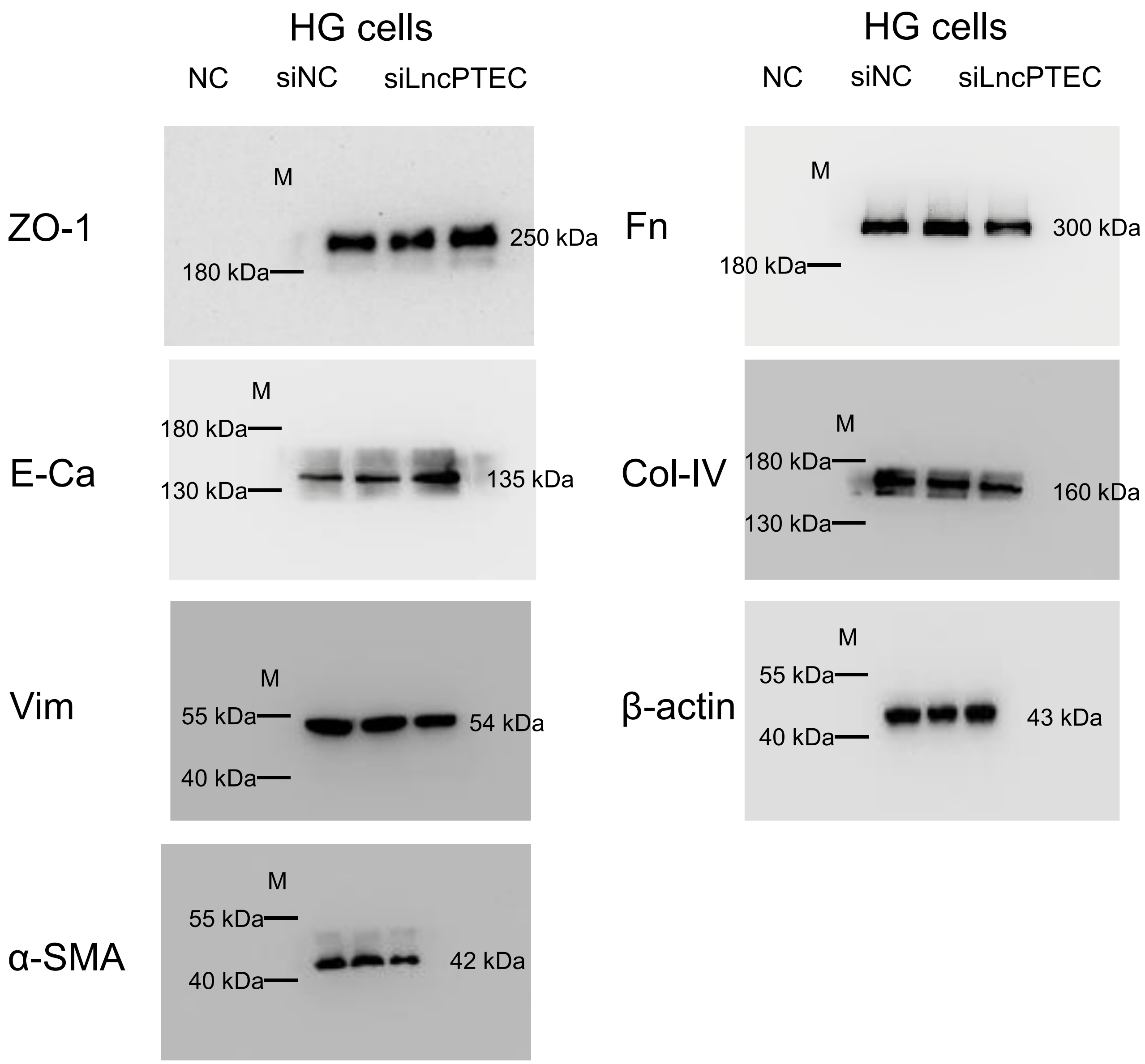

Fig4G
LG cells

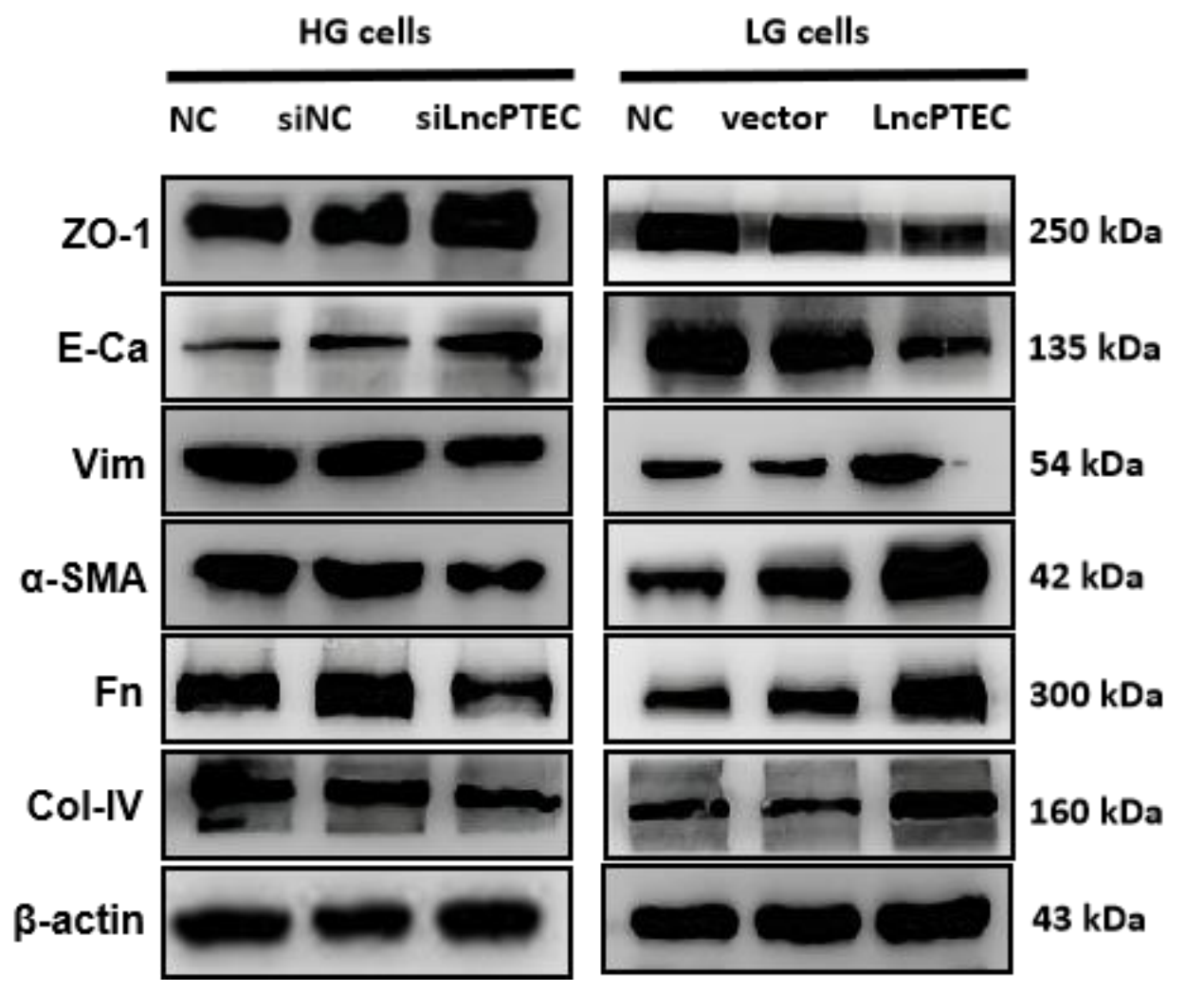

Original unprocessed gel

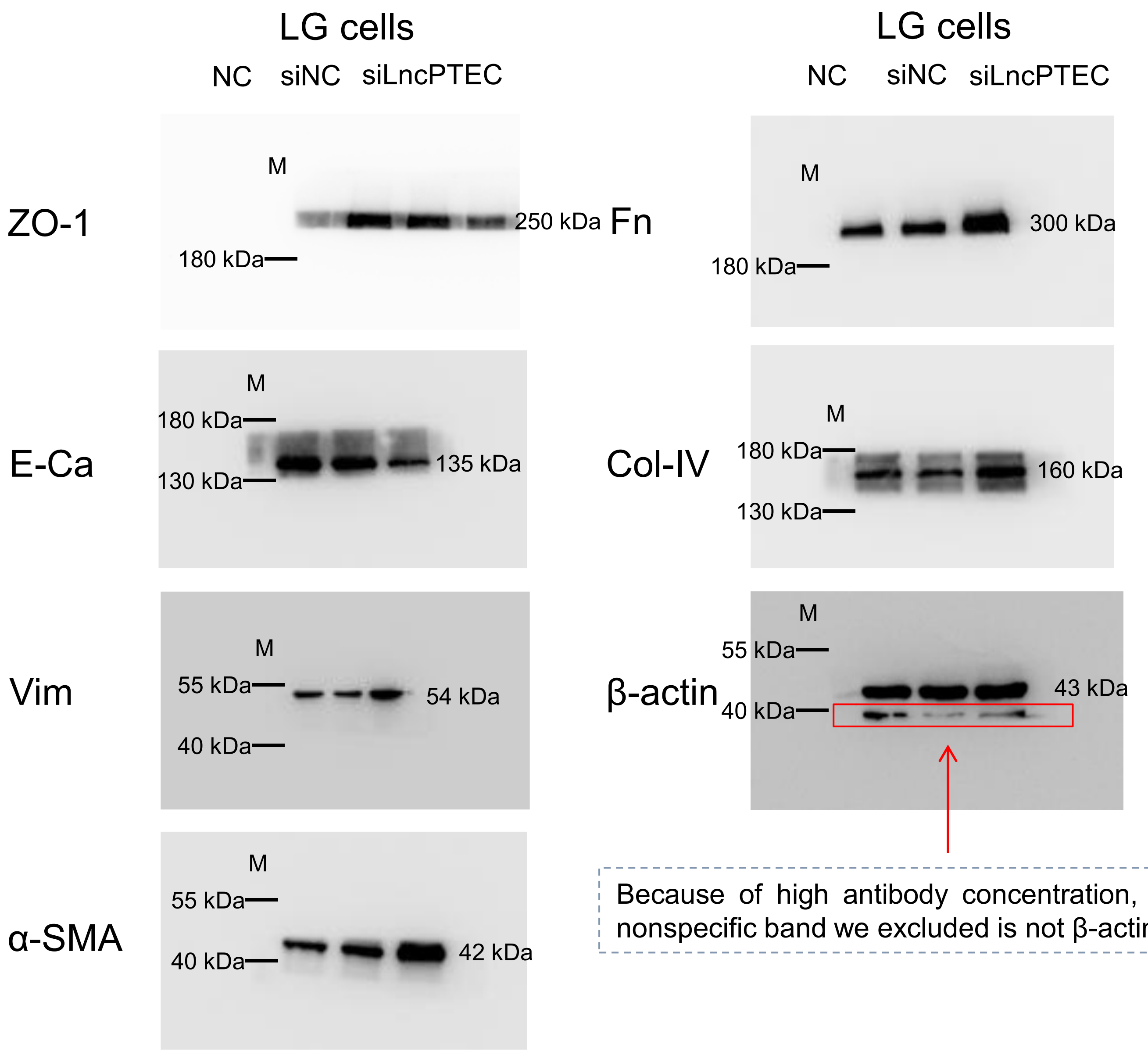

Because of high antibody concentration, the nonspecific band we excluded is not β-actin.

Fig7C

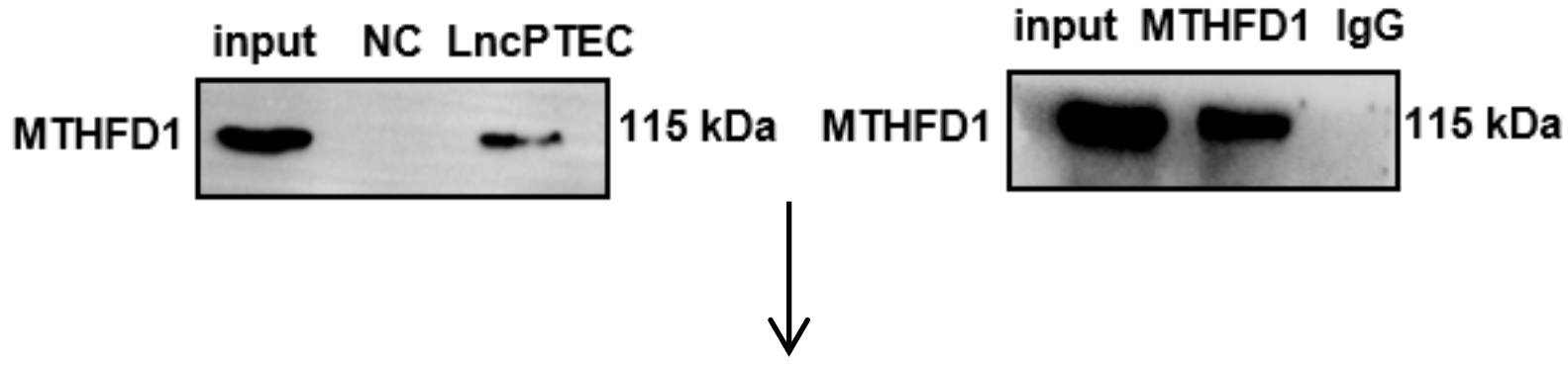

Original unprocessed gel

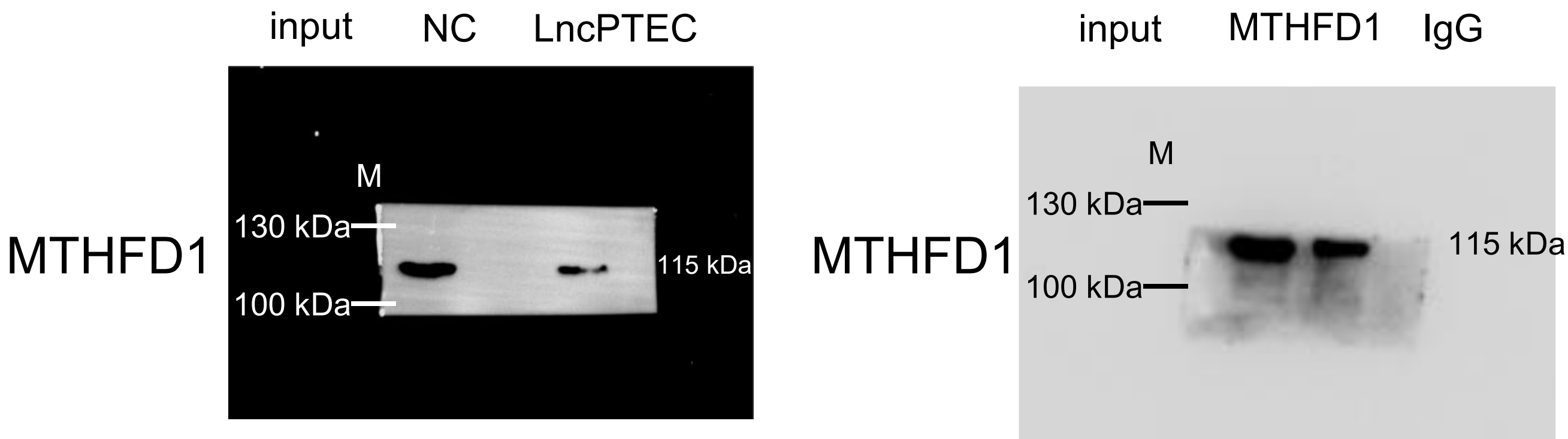

Fig7F

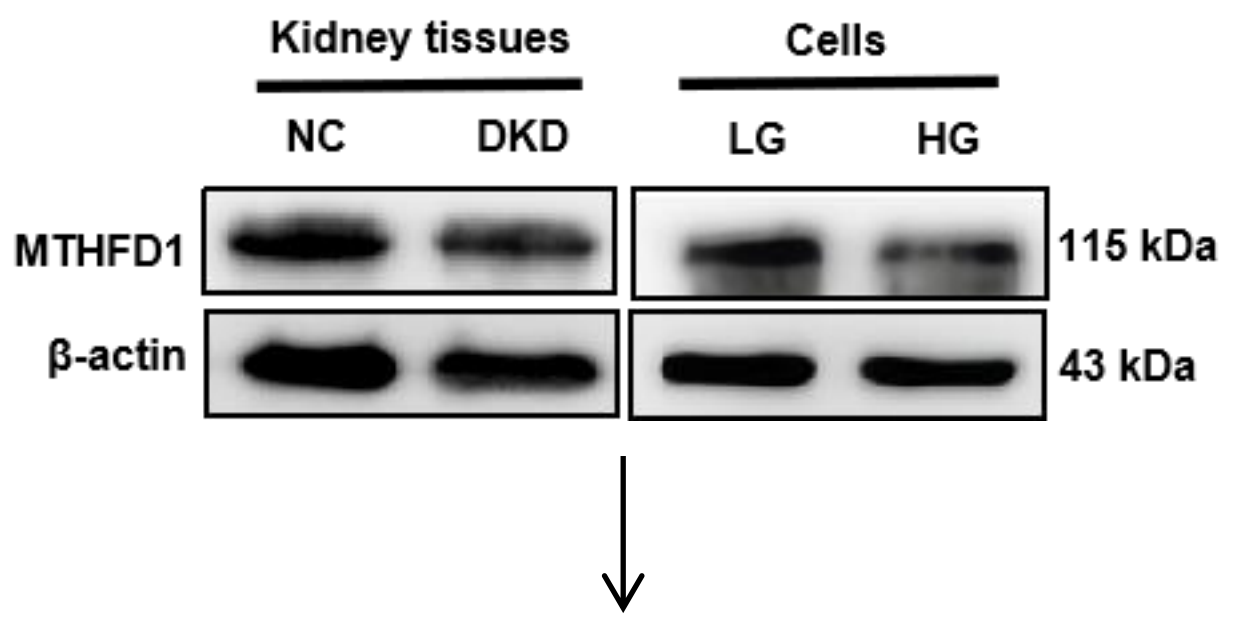

Original unprocessed gel

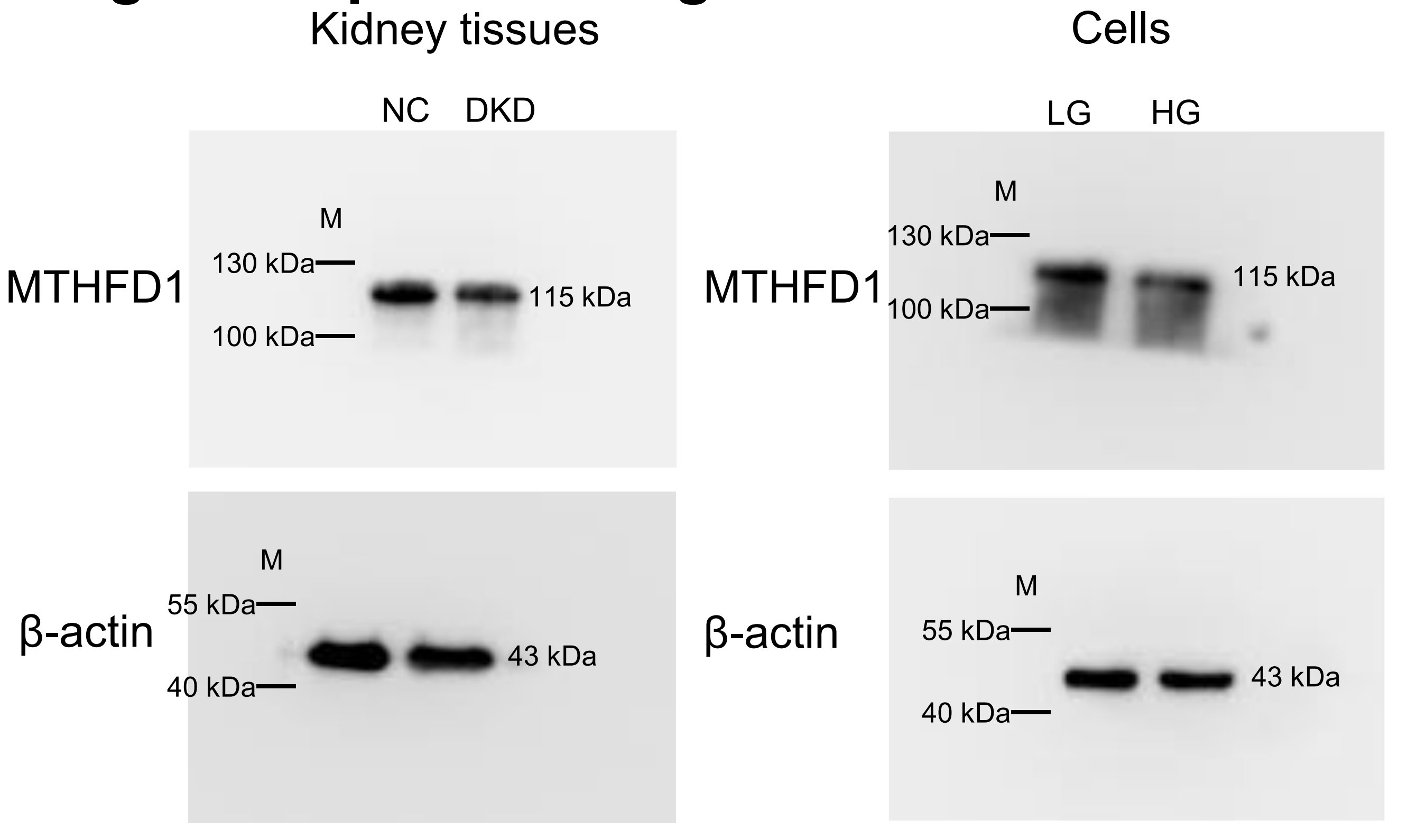

Fig7G

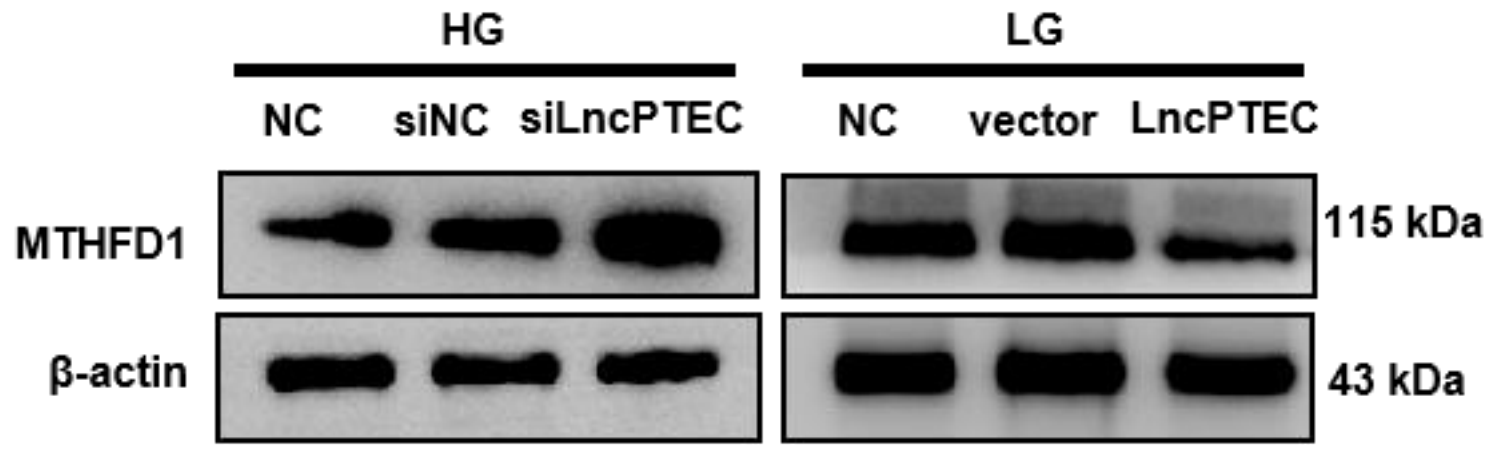

Original unprocessed gel

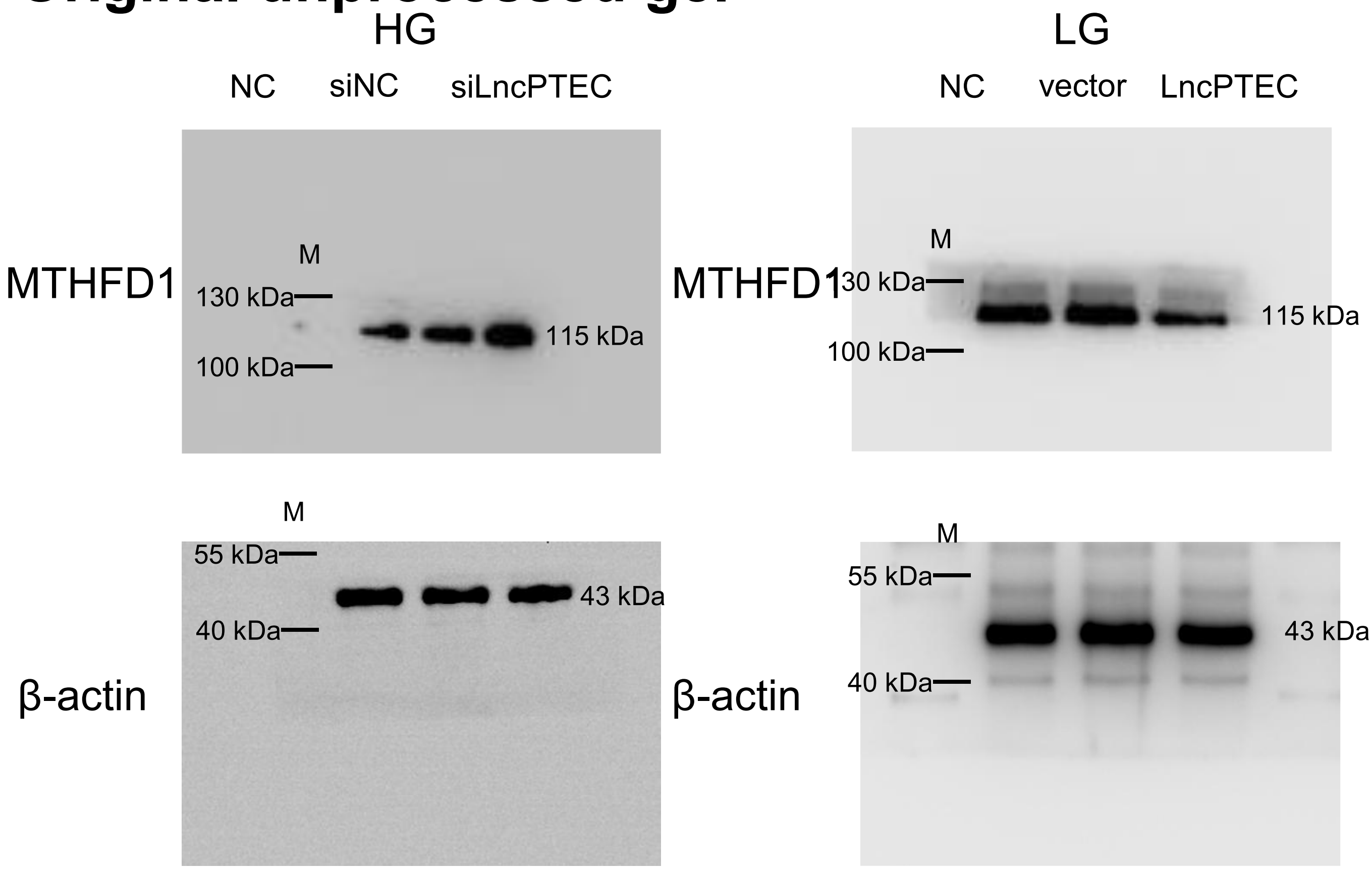

Fig7K-top

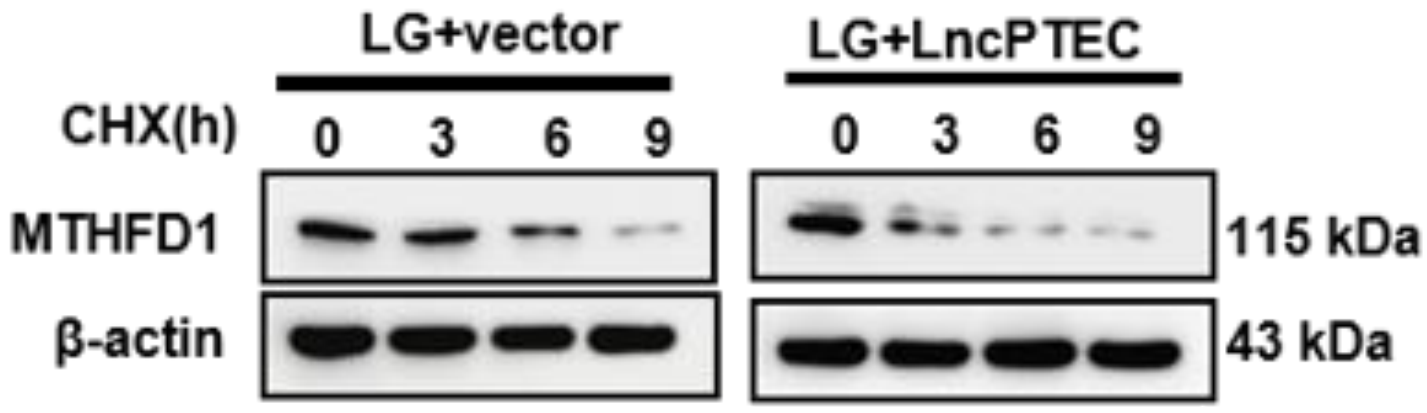

Original unprocessed gel

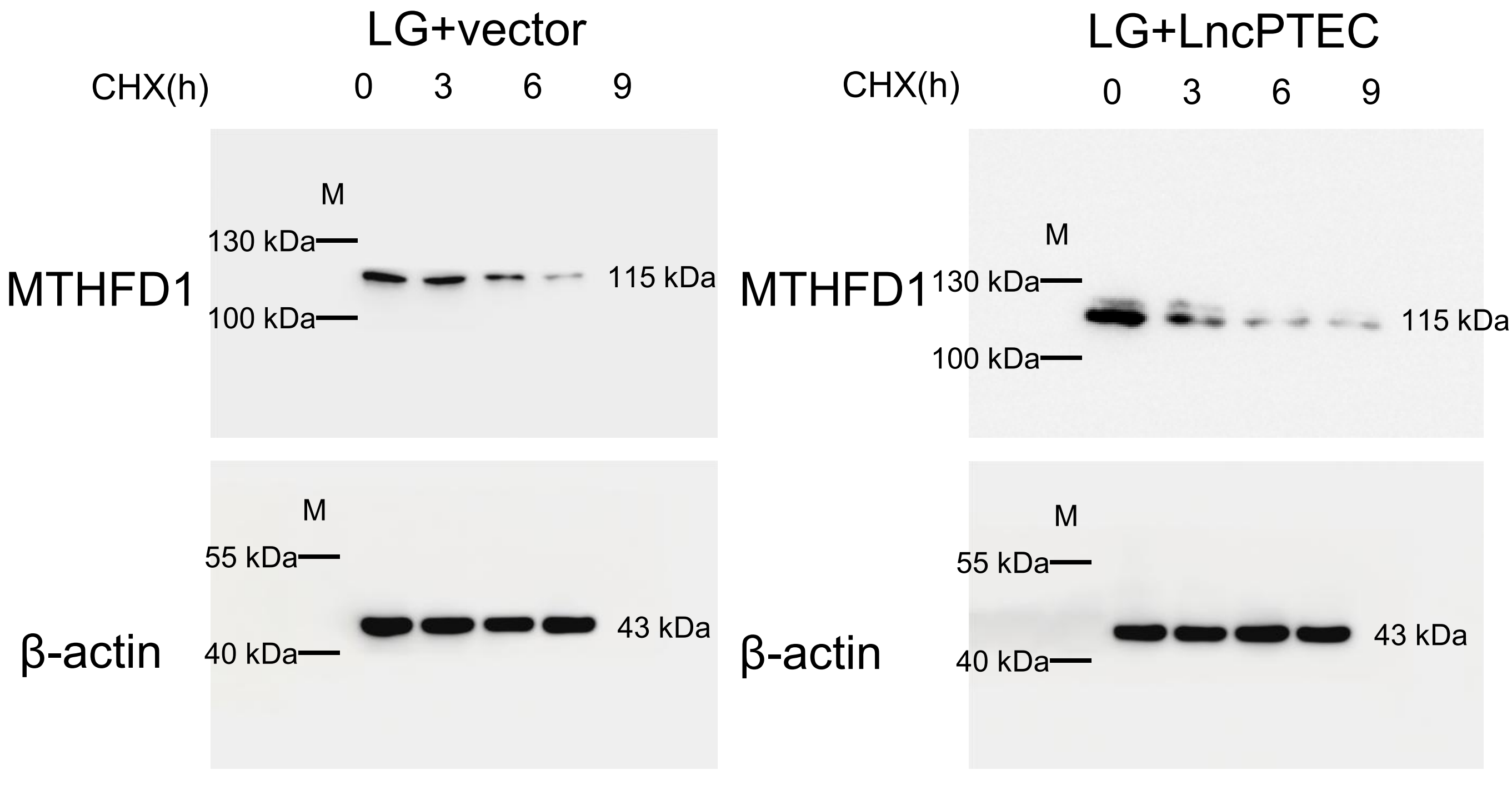

Fig7K-bottom

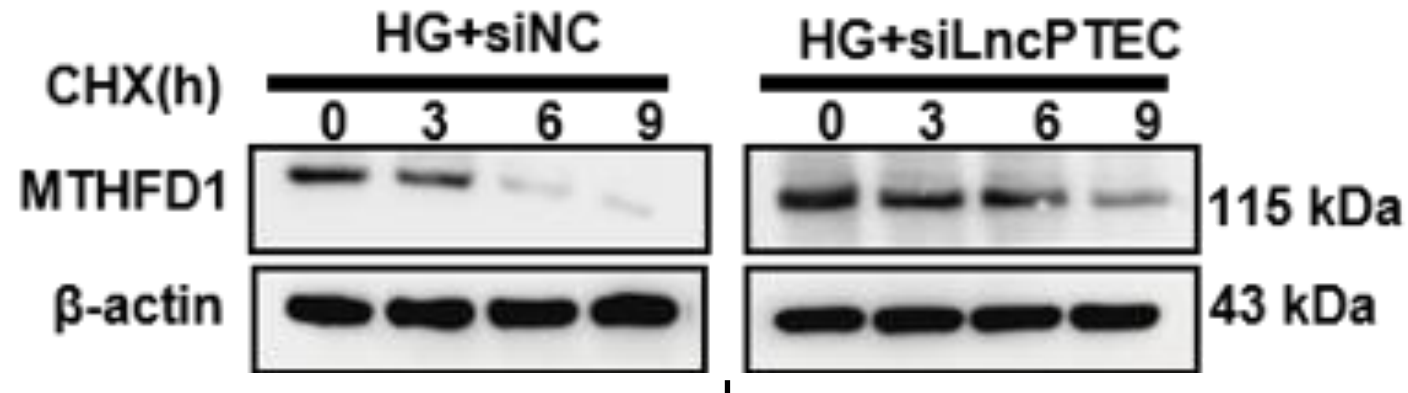

Original unprocessed gel

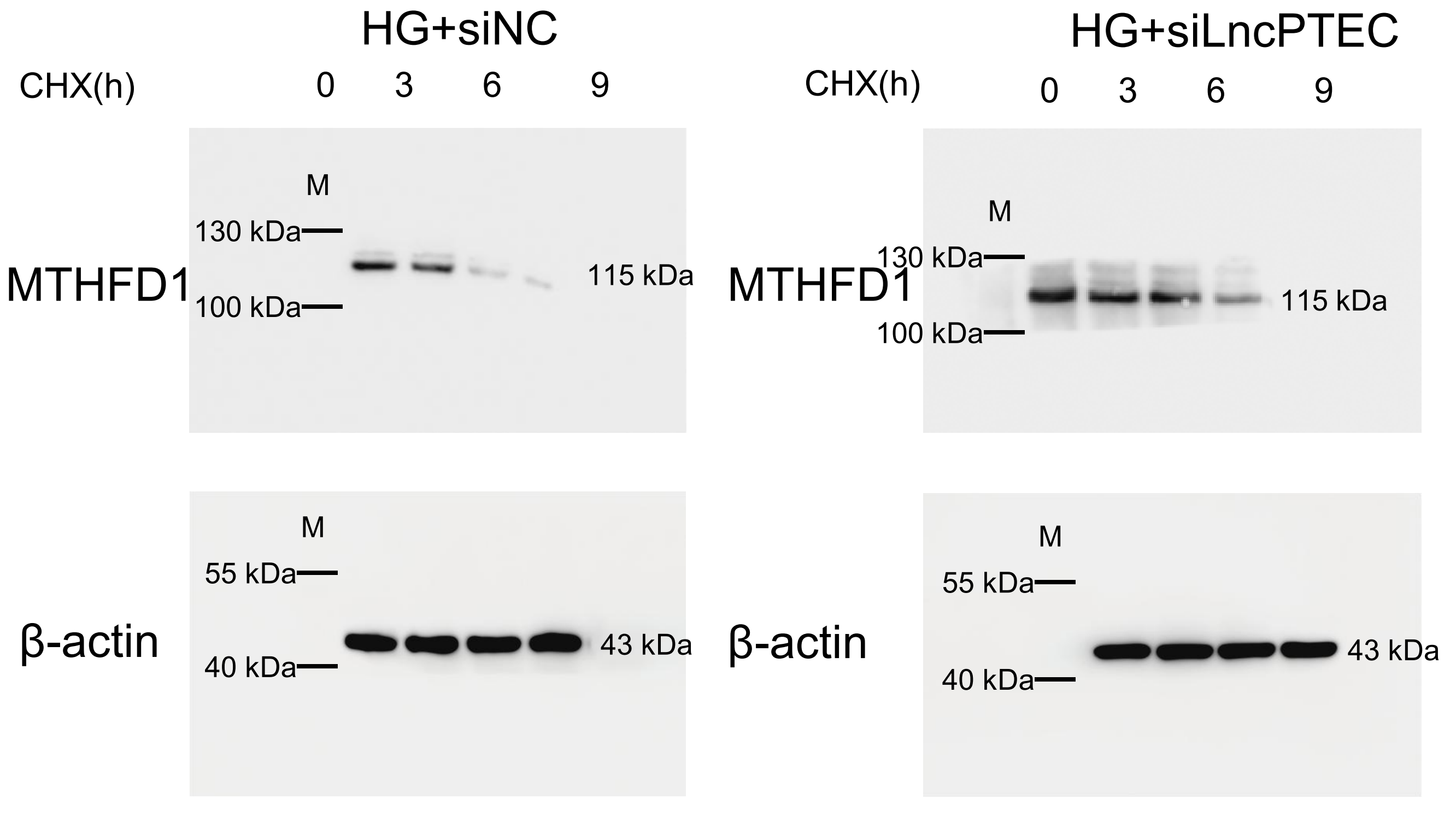

Fig7L

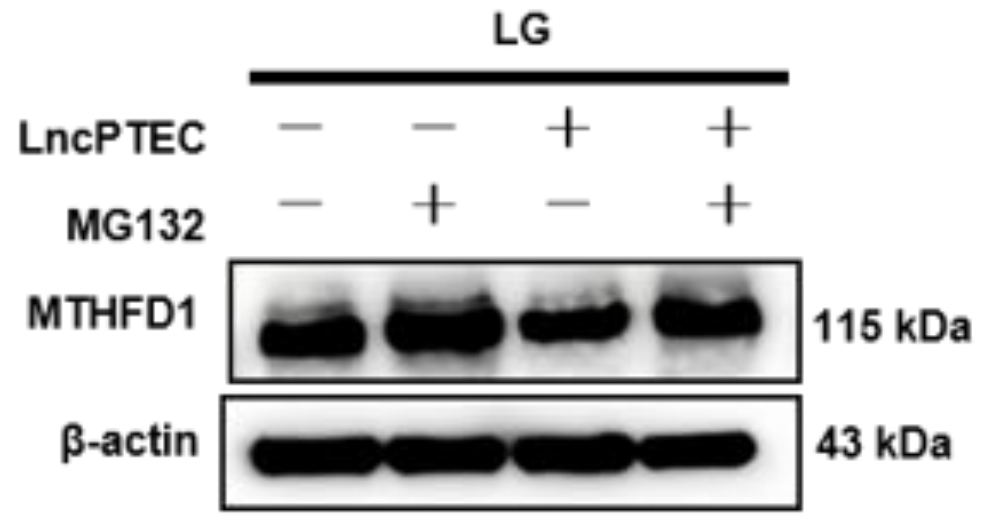

Original unprocessed gel

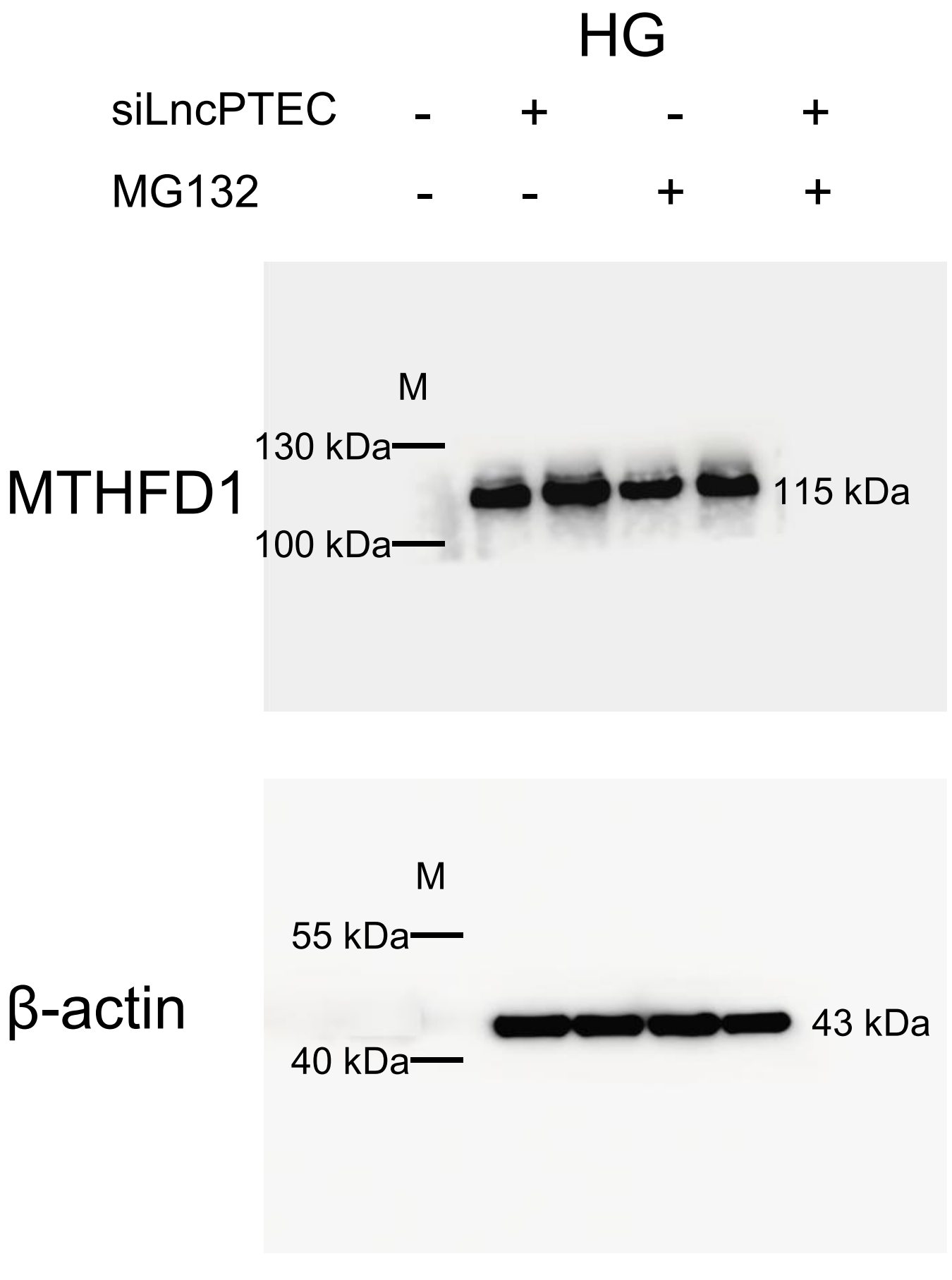

Fig7M

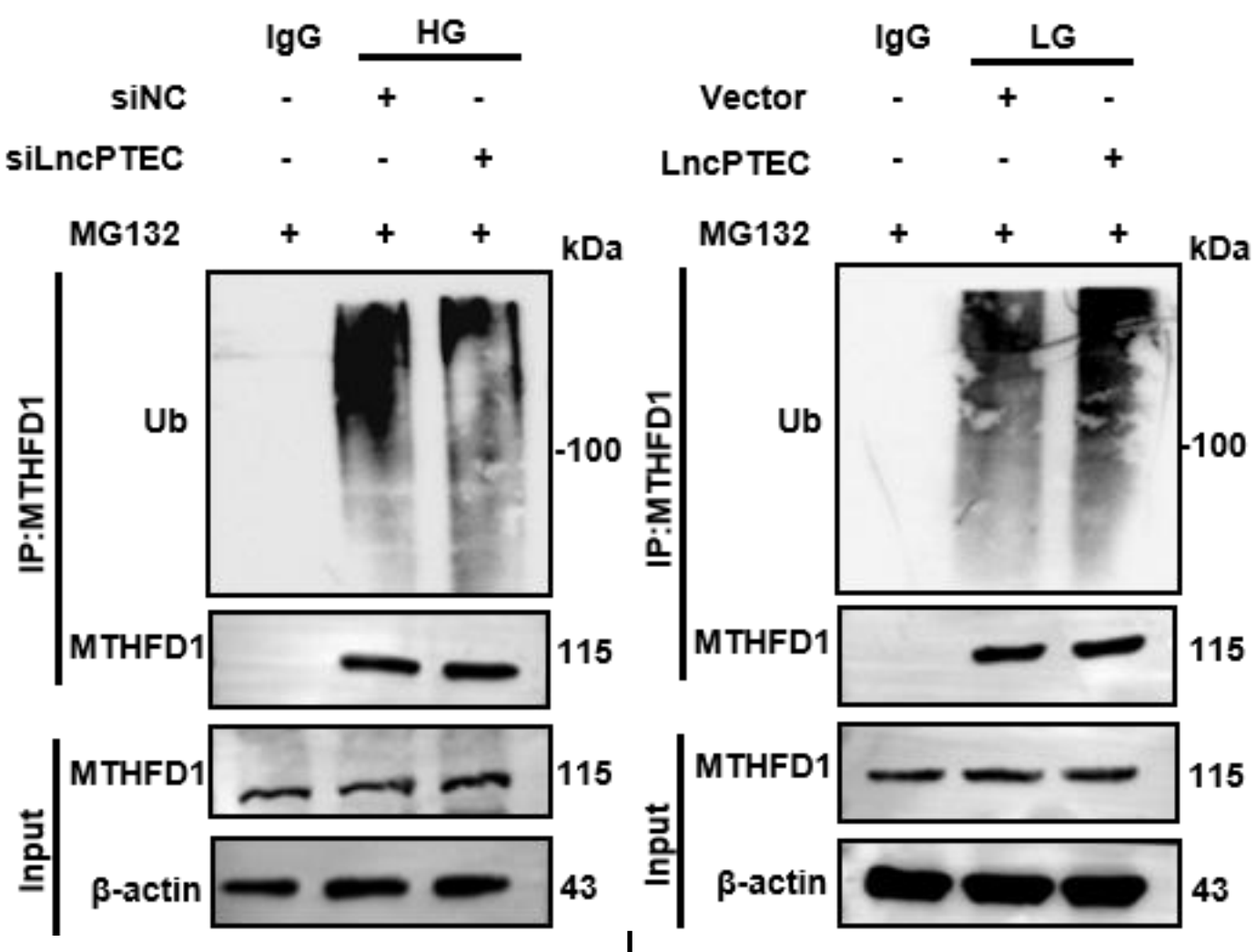

Original unprocessed gel

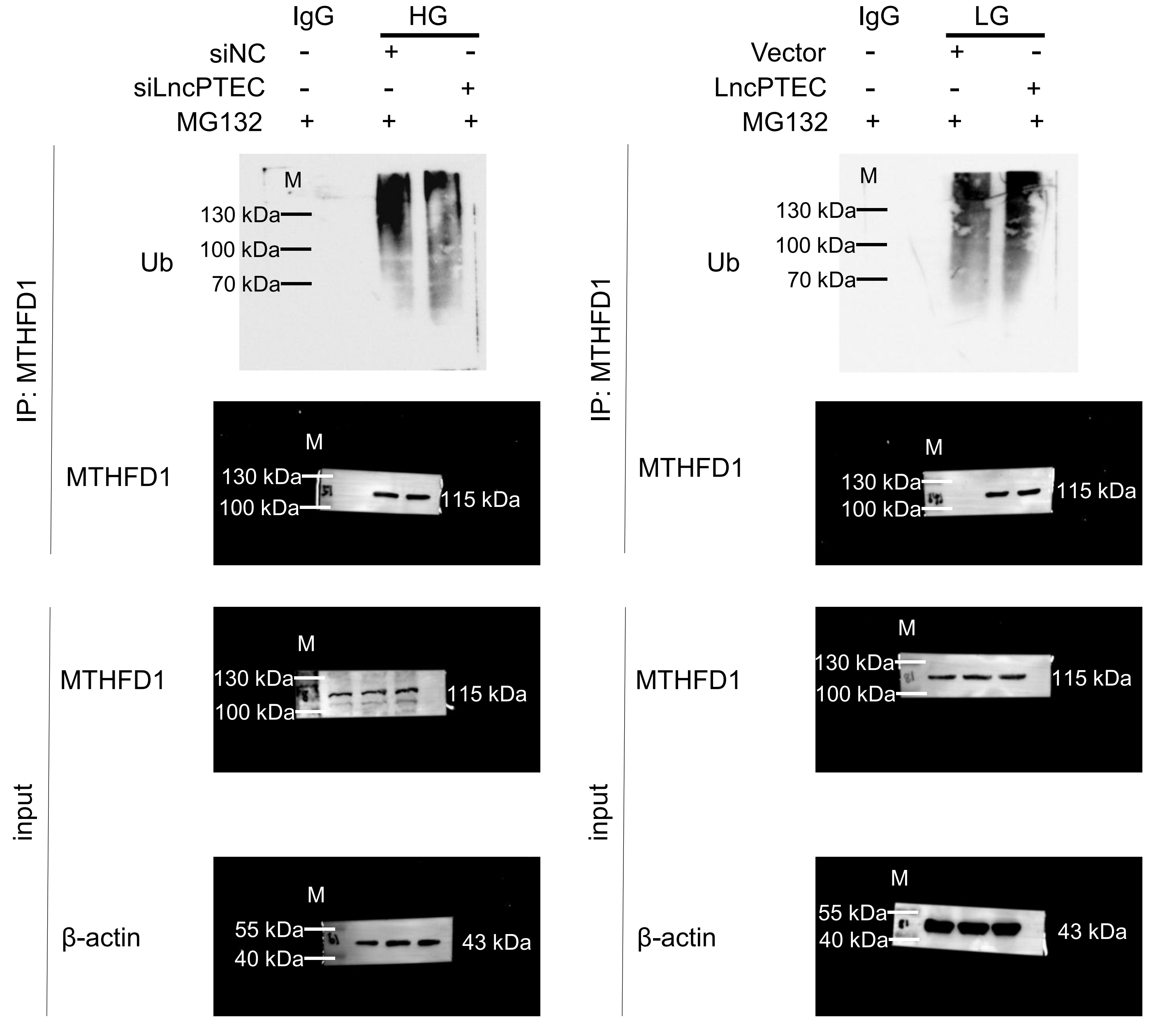

Fig7N-left

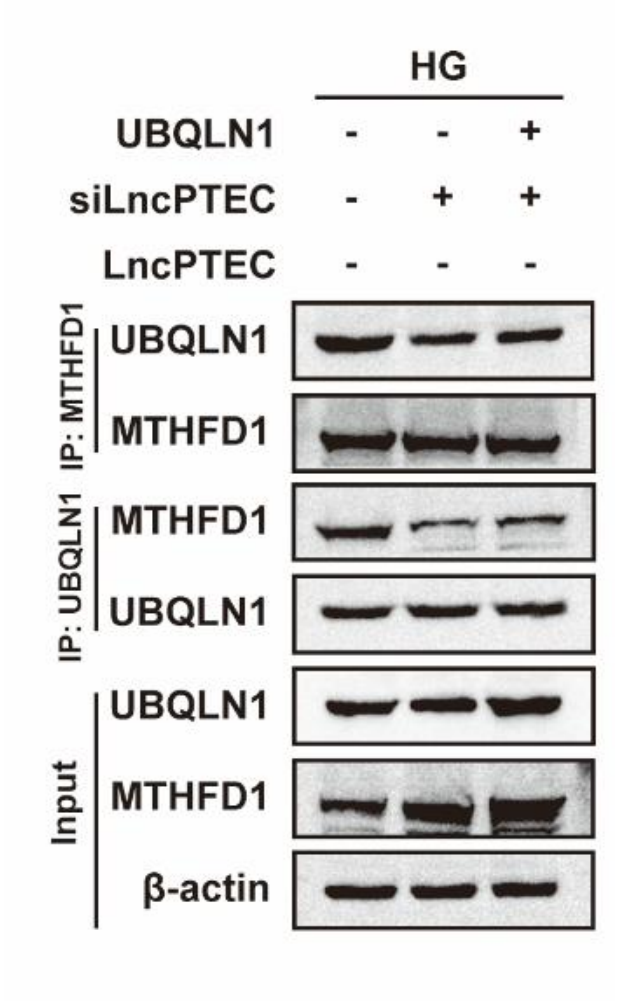

Original unprocessed gel

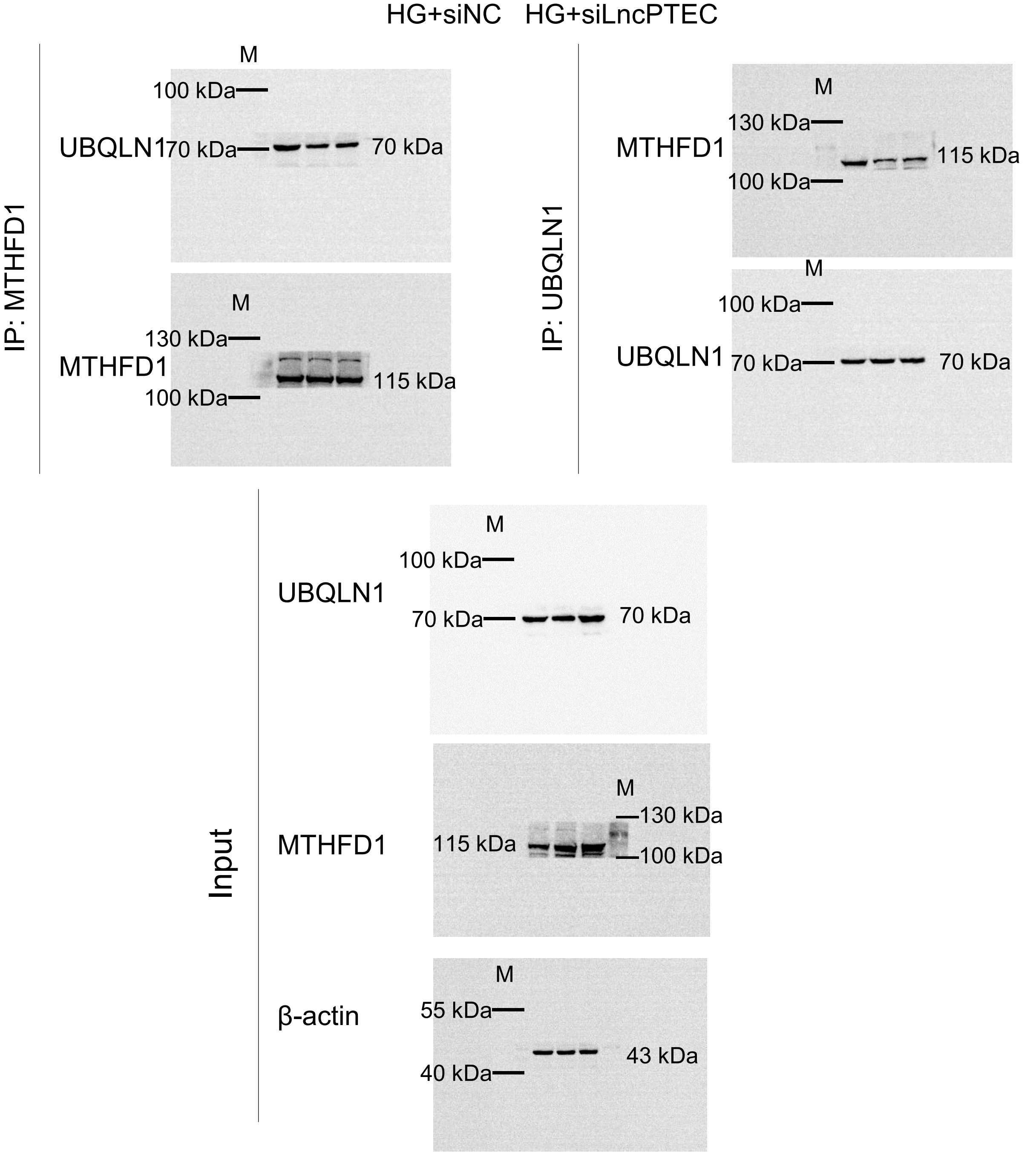

Fig7N-right

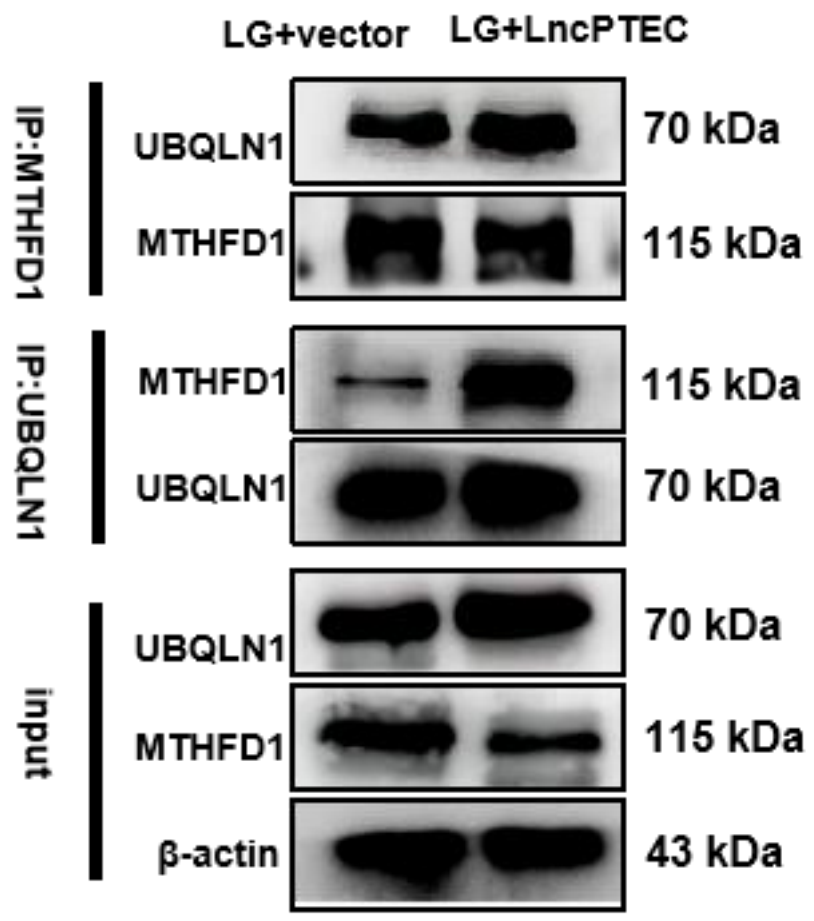

Original unprocessed gel

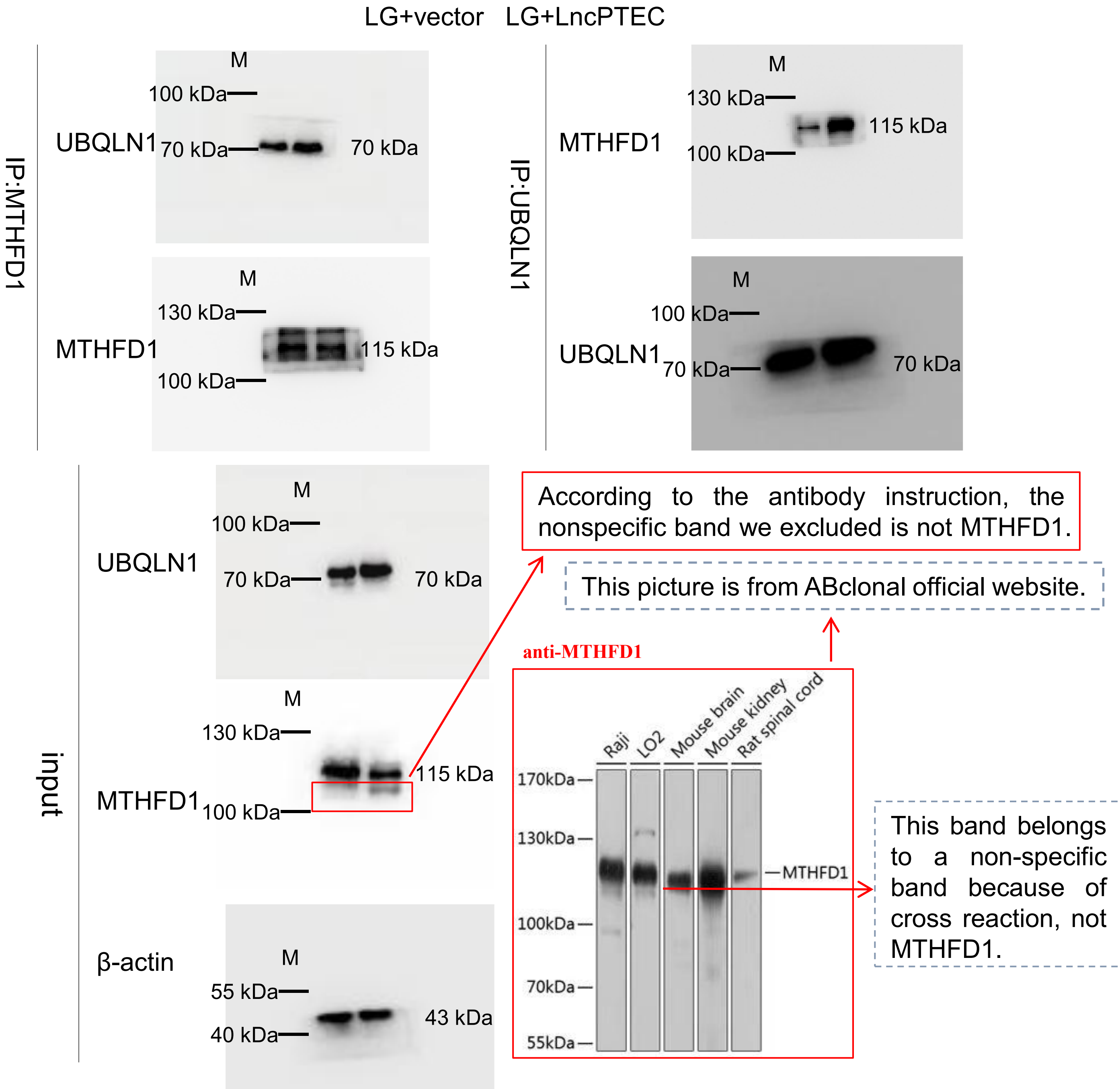

Fig8F
HG

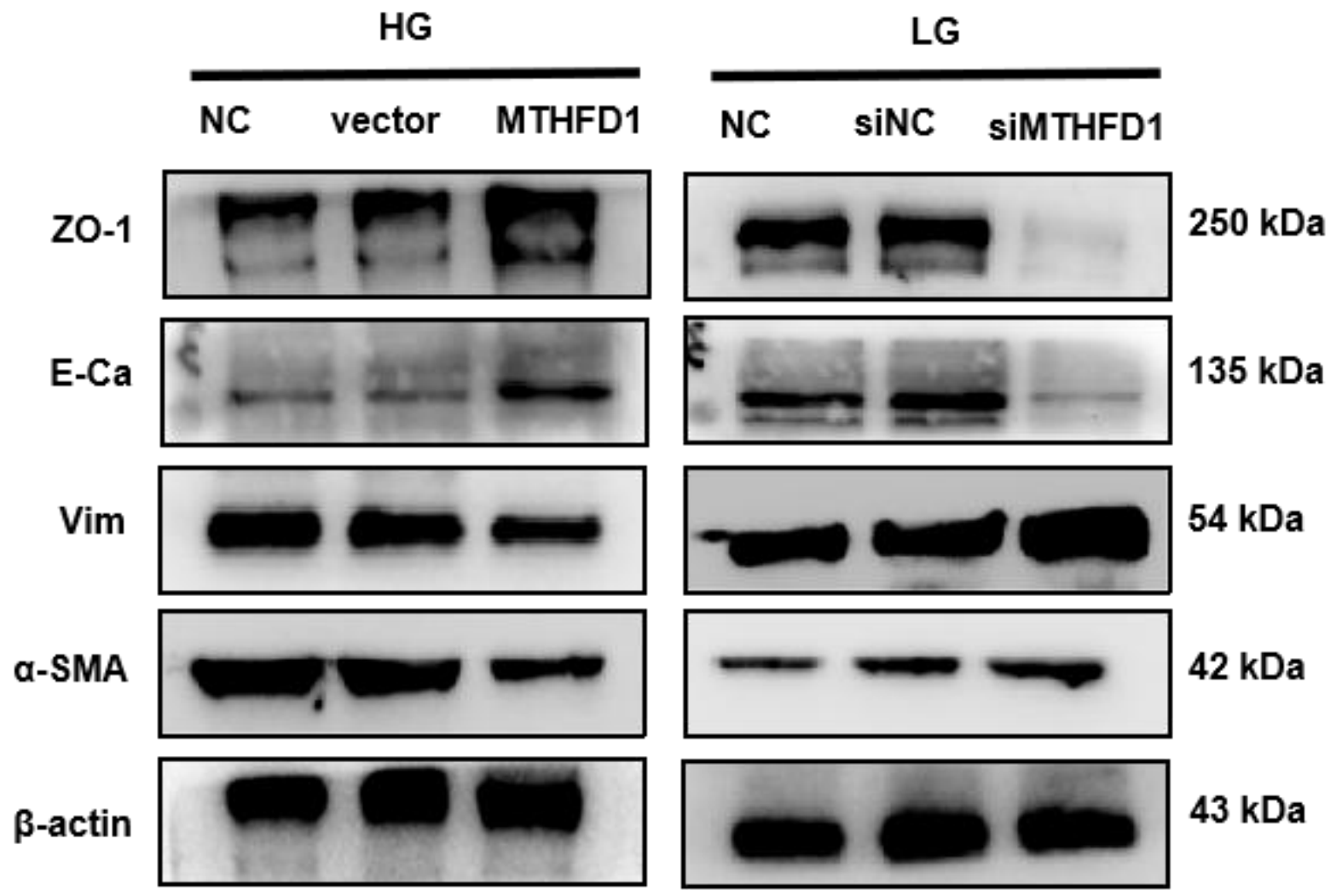

According to the antibody instruction, the nonspecific band we excluded is not ZO-1.

Original unprocessed gel

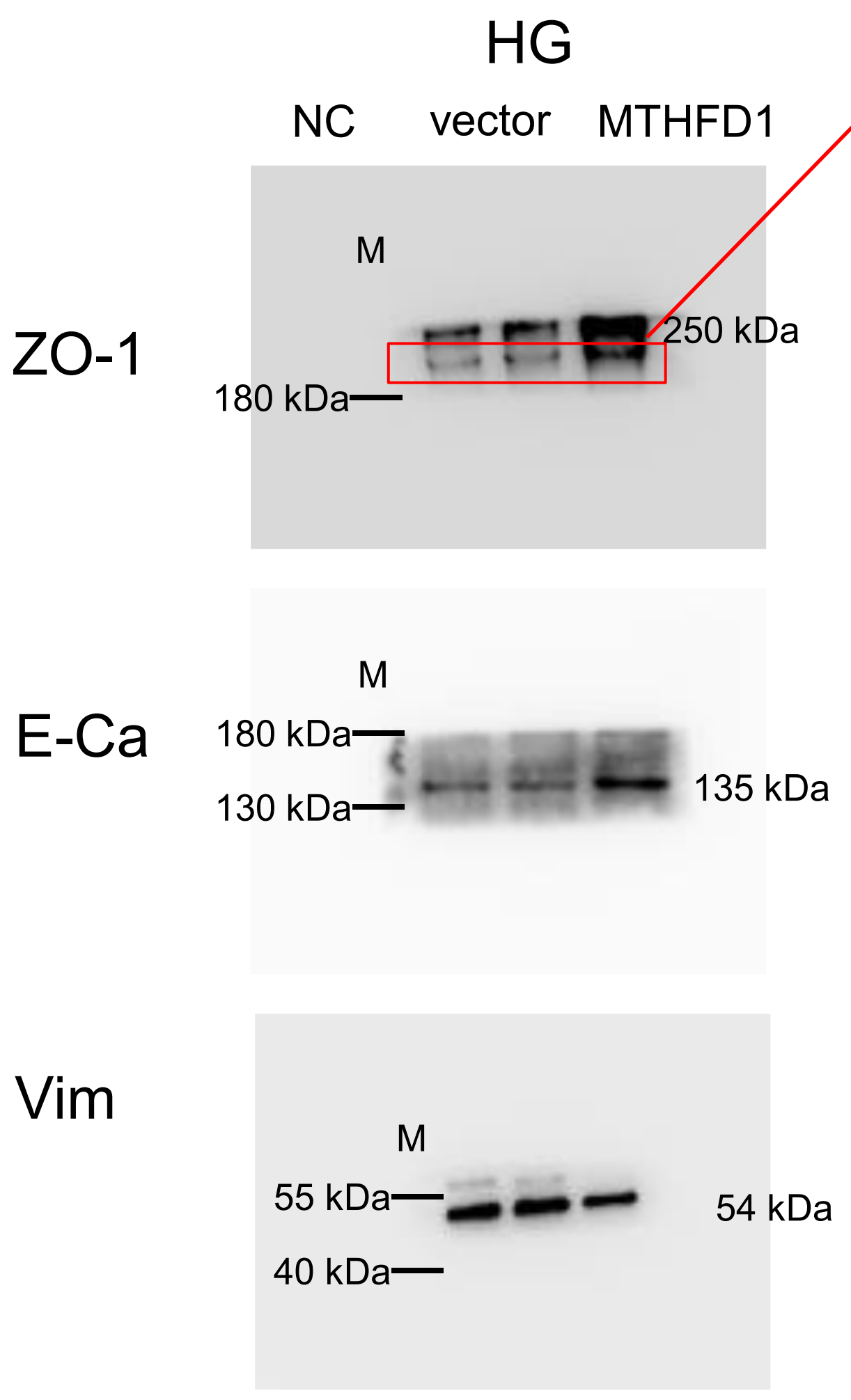

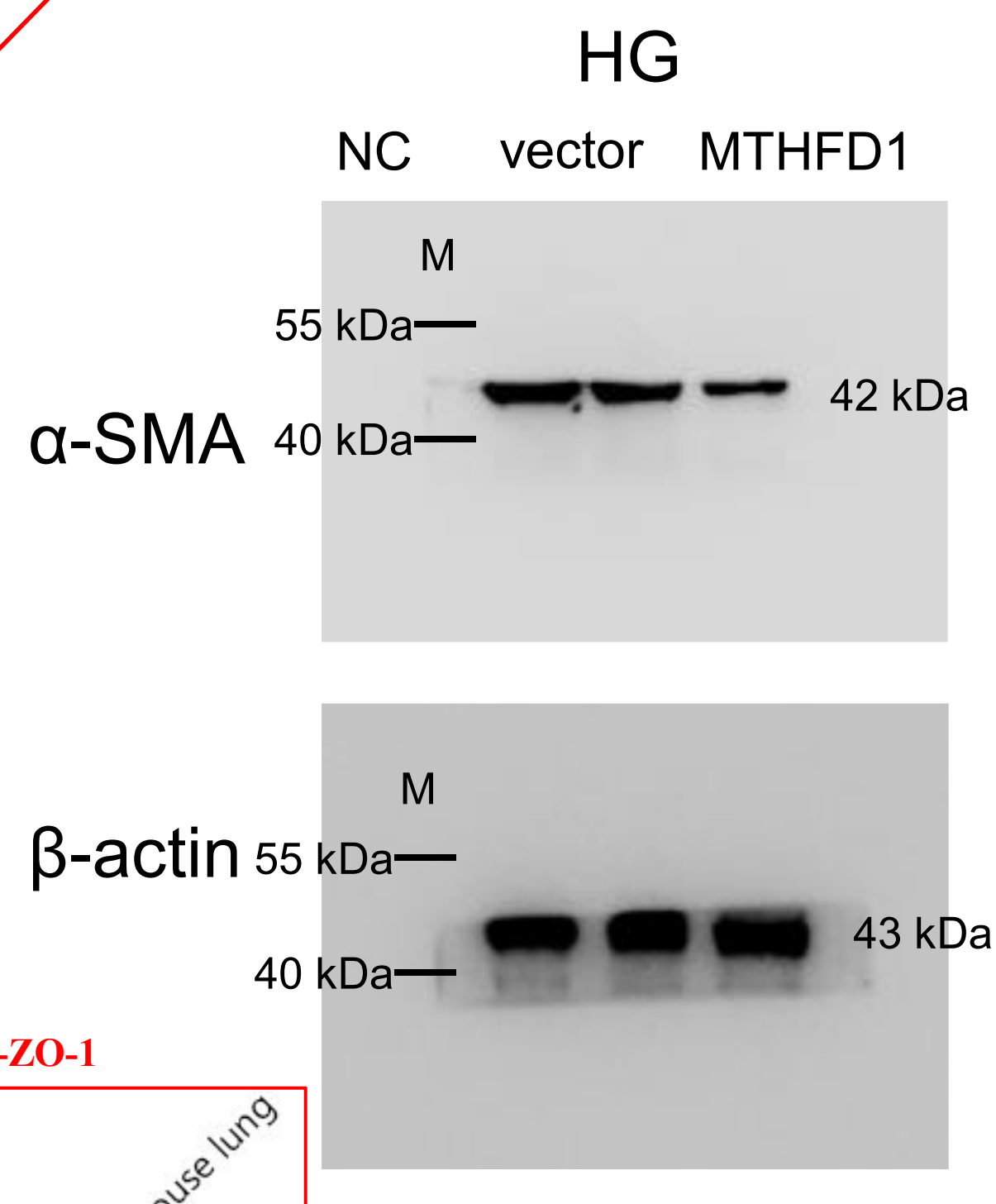

anti-ZO-1

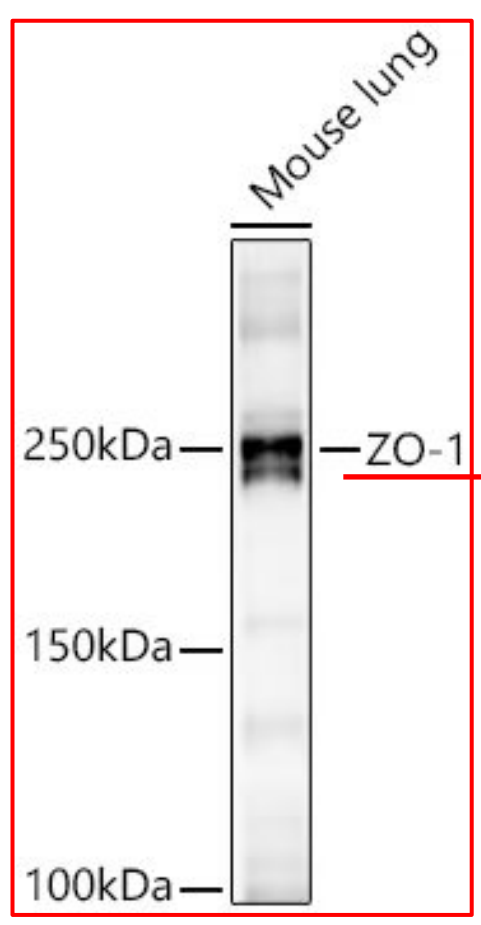

This band belongs to a non-specific band because of cross reaction, not ZO-1.

This picture is from ABclonal official website.

Fig8F
LG

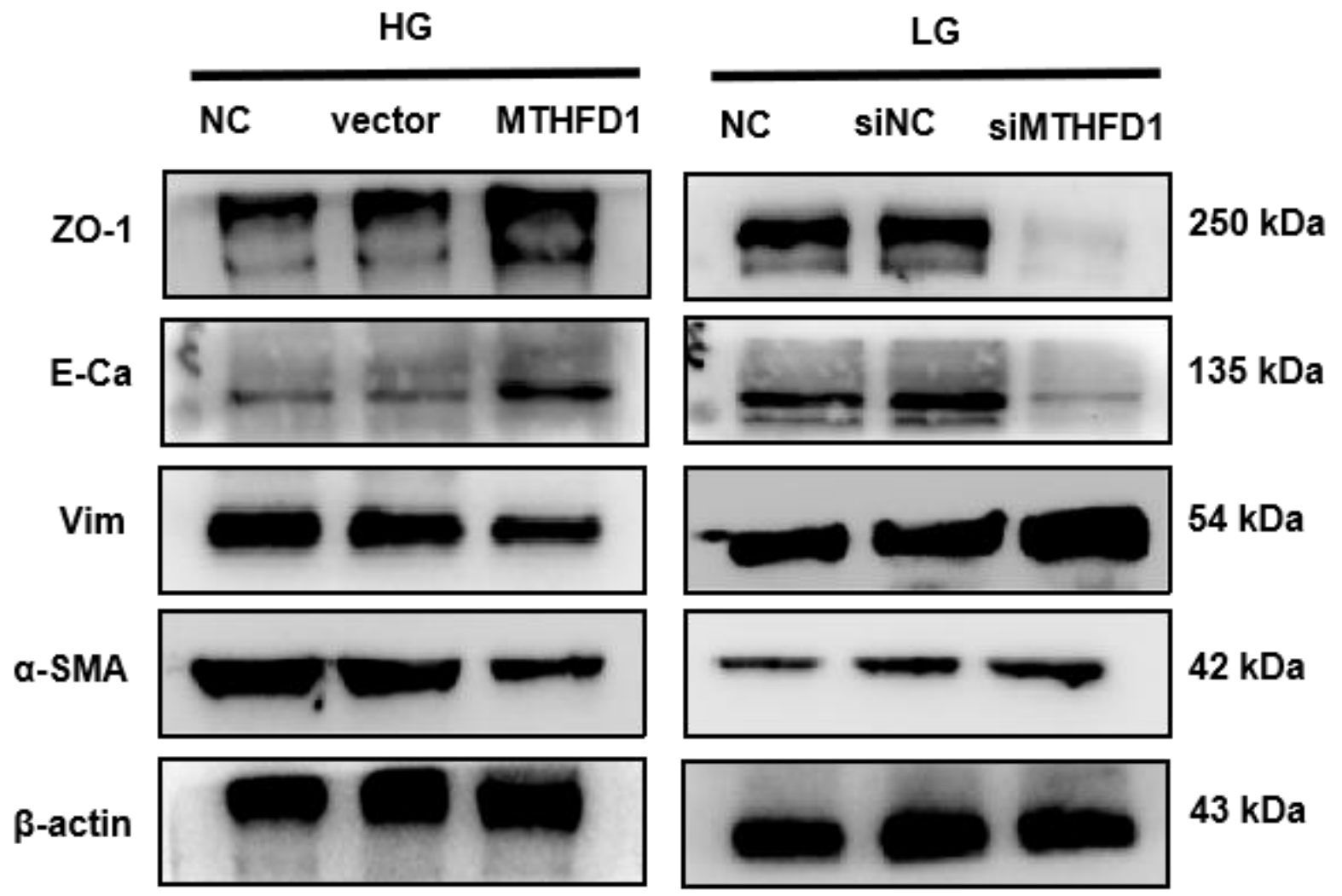

According to the antibody instruction, the nonspecific band we excluded is not ZO-1.

Original unprocessed gel

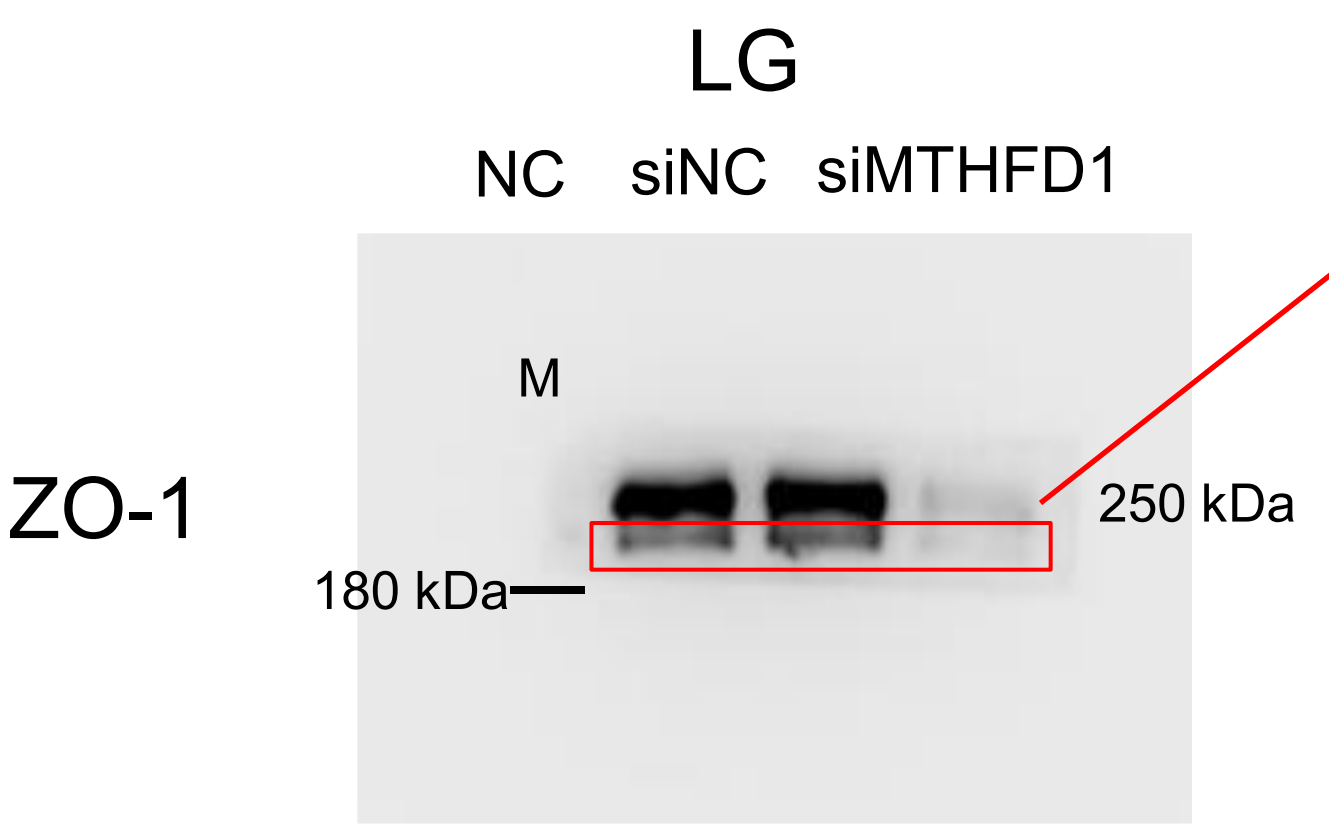

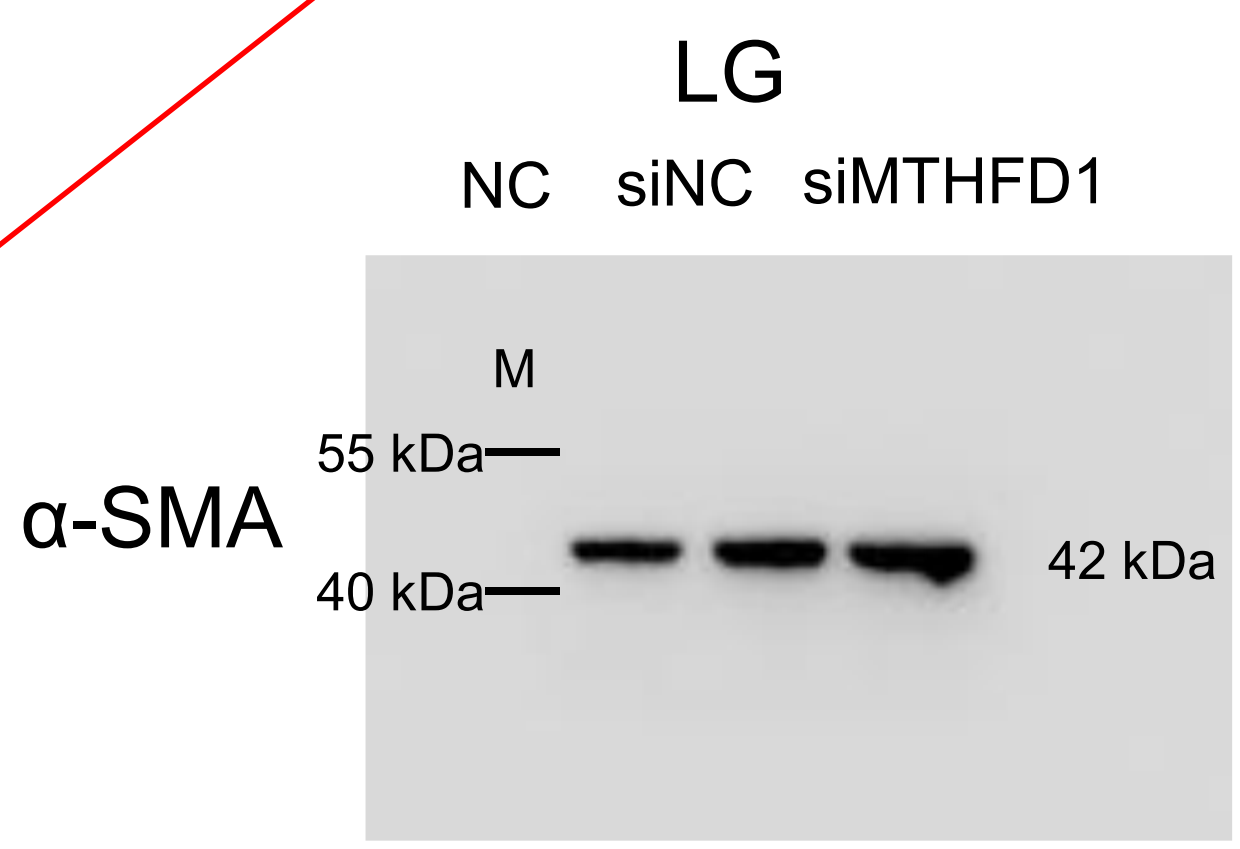

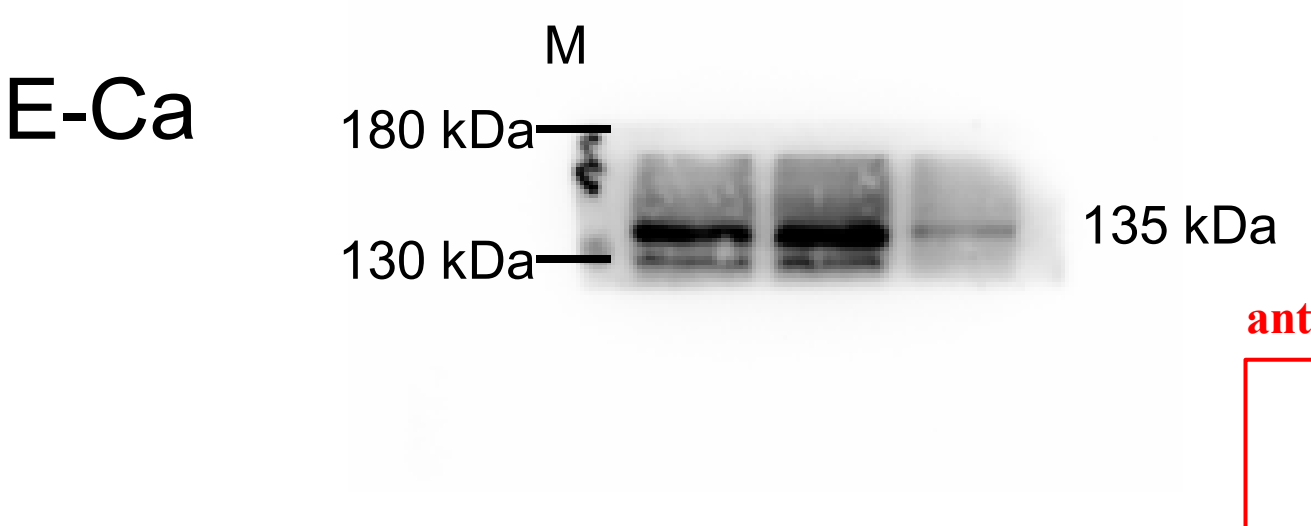

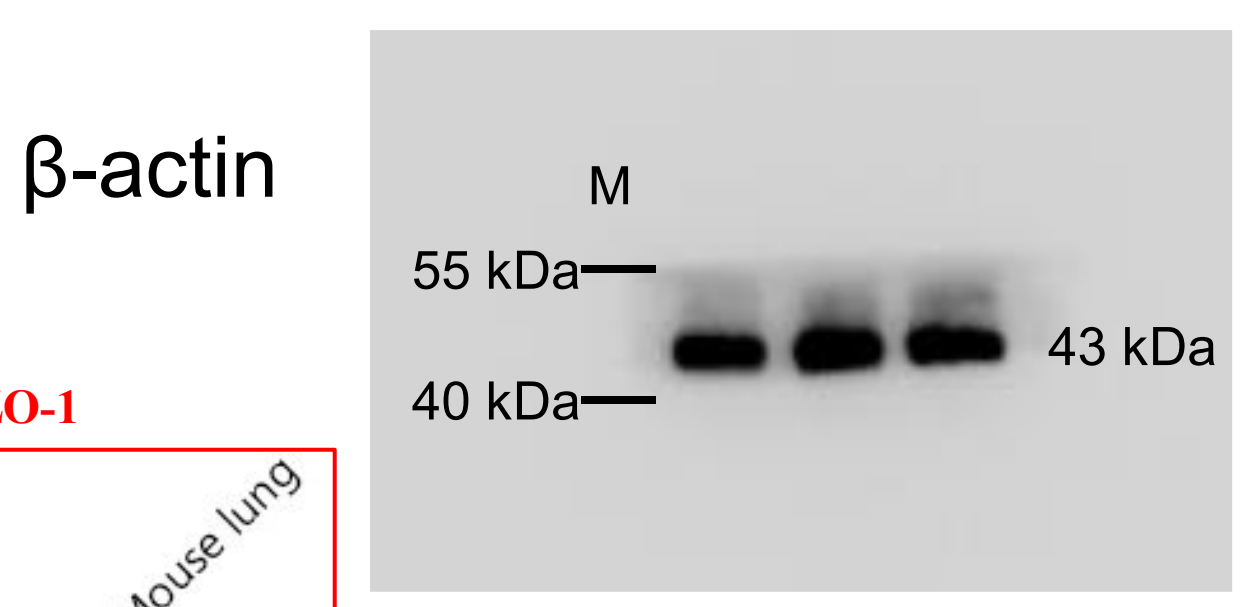

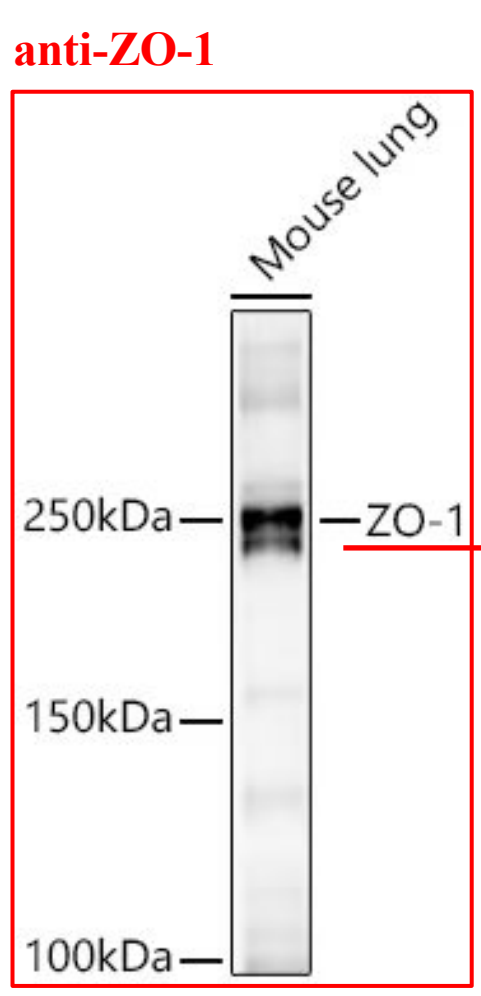

This band belongs to a non-specific band because of cross reaction, not ZO-1.

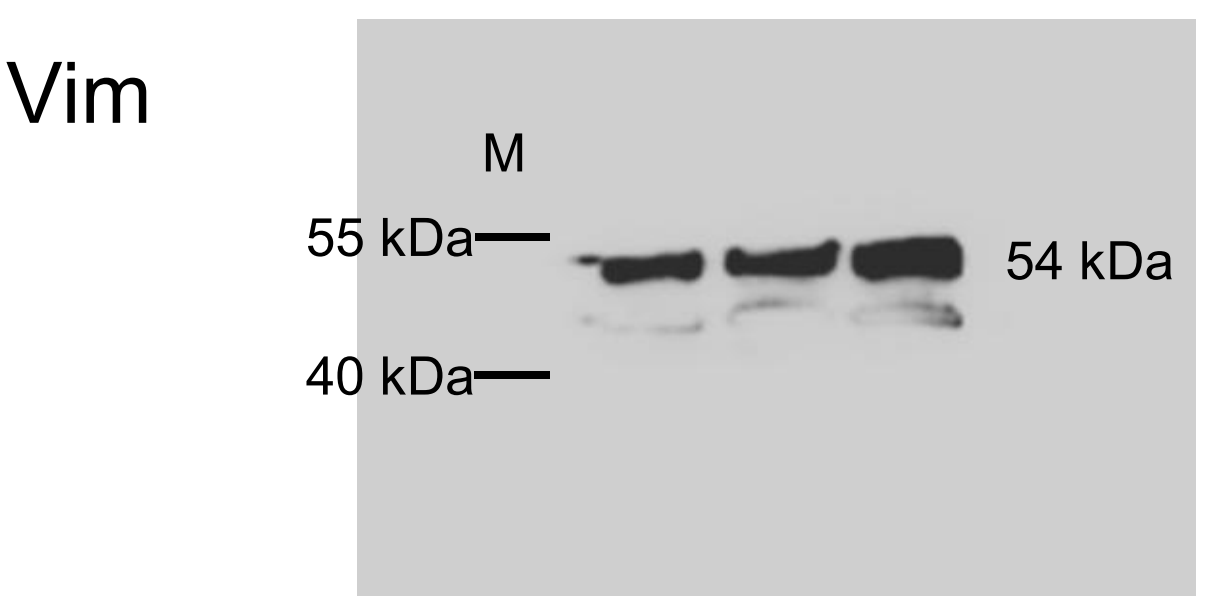

This picture is from ABclonal official website.

Fig9F
HG

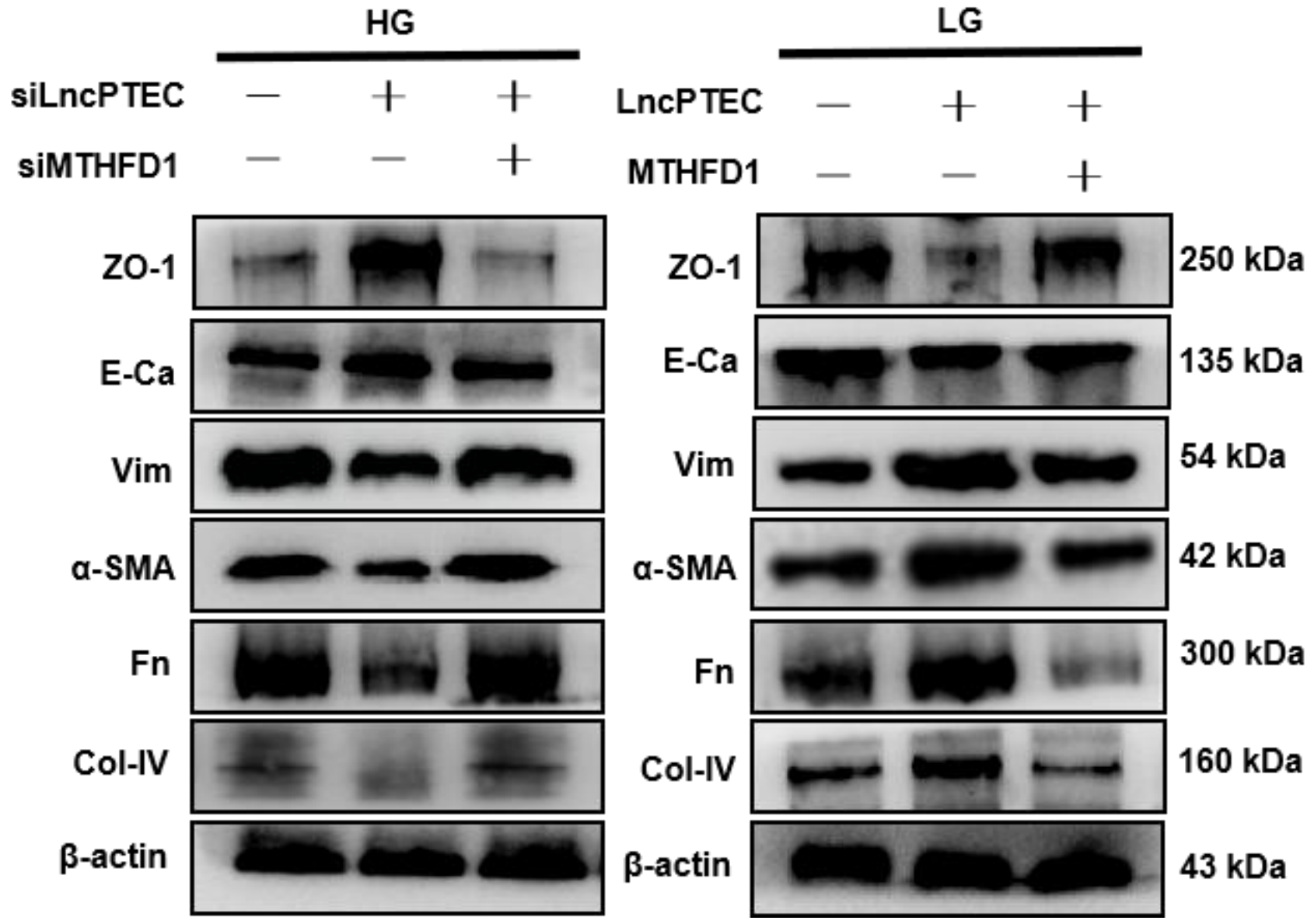

Original unprocessed gel

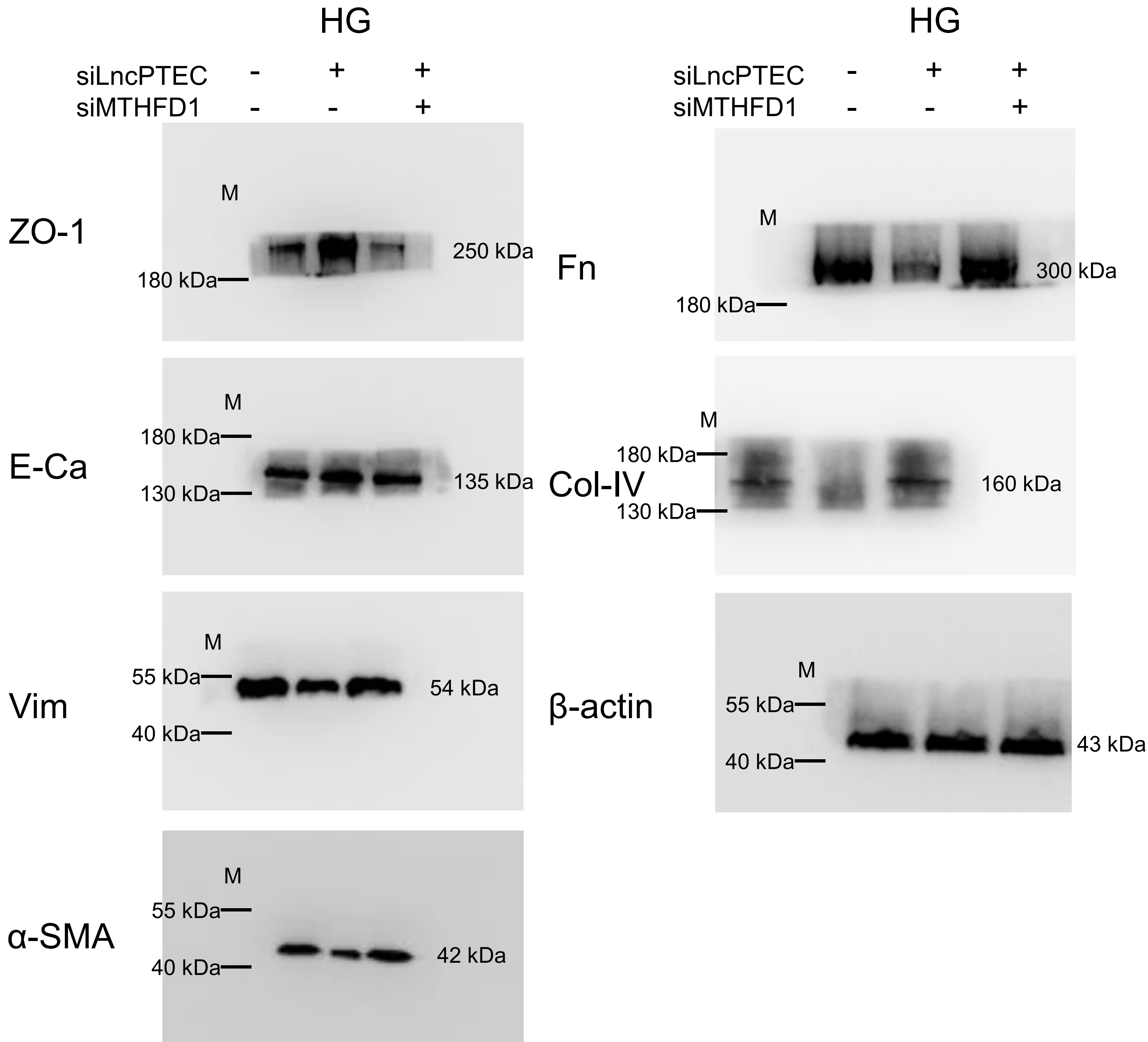

Fig9F
LG

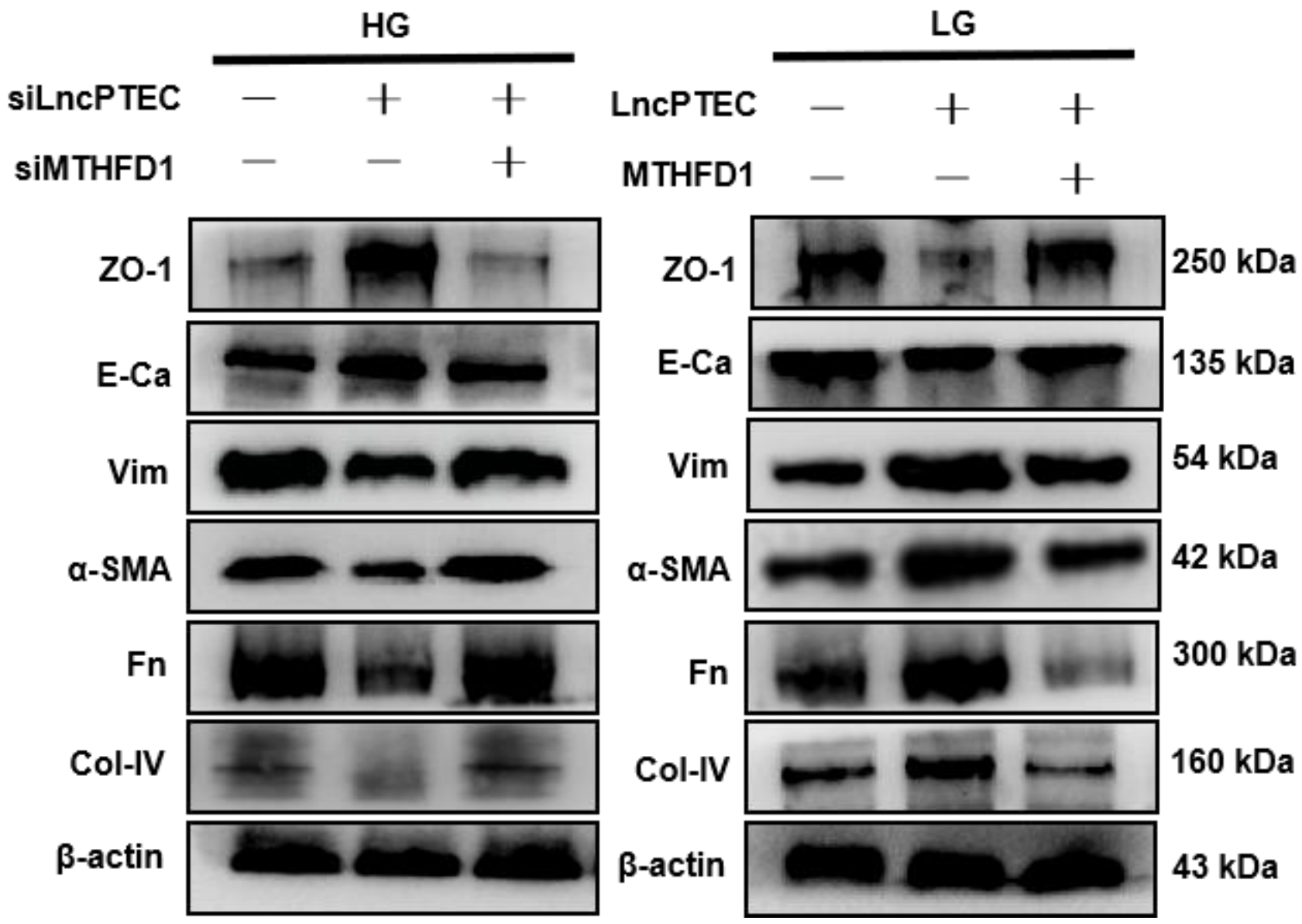

Original unprocessed gel

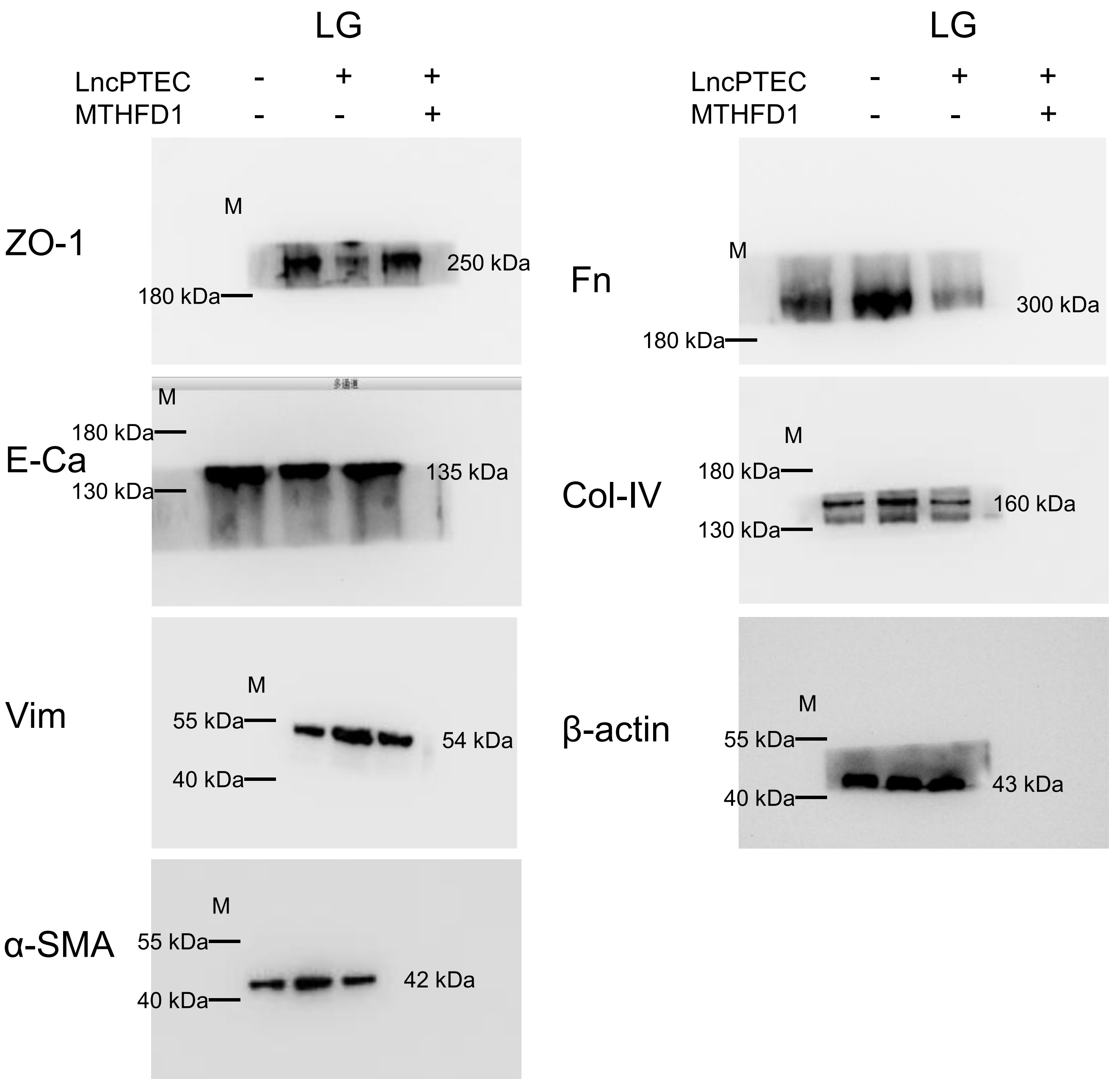

FigS5

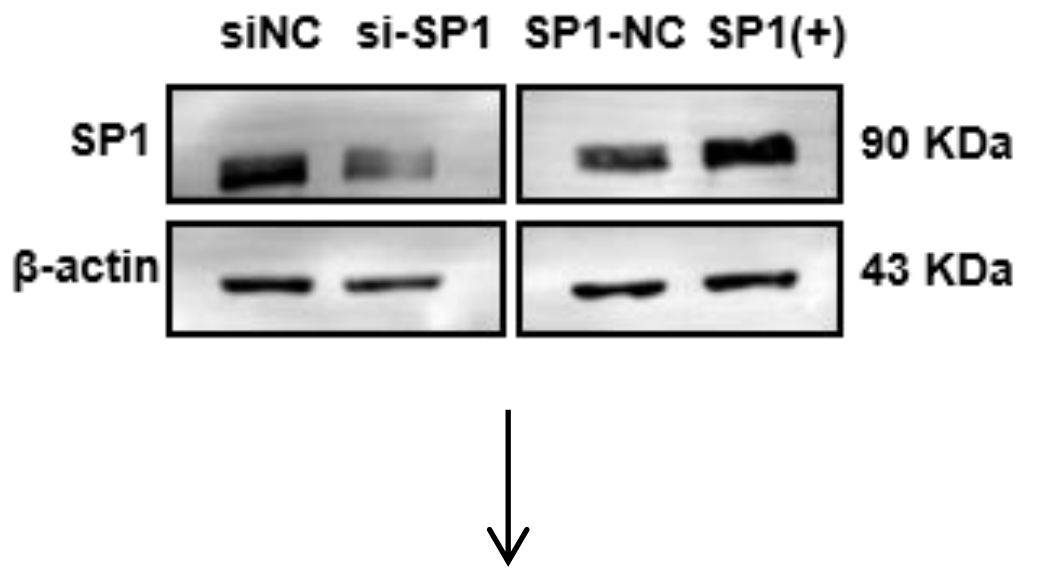

Original unprocessed gel

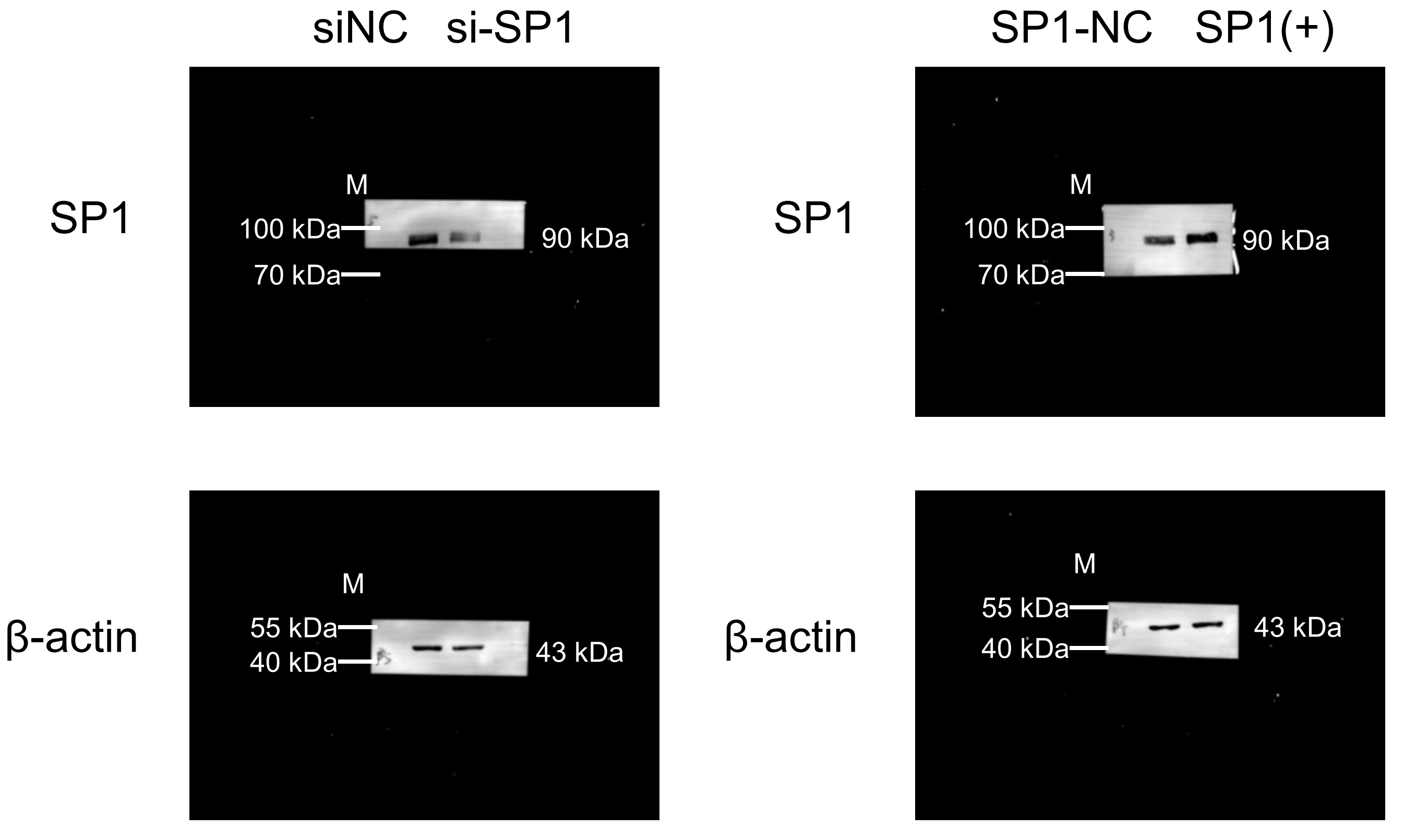

FigS6

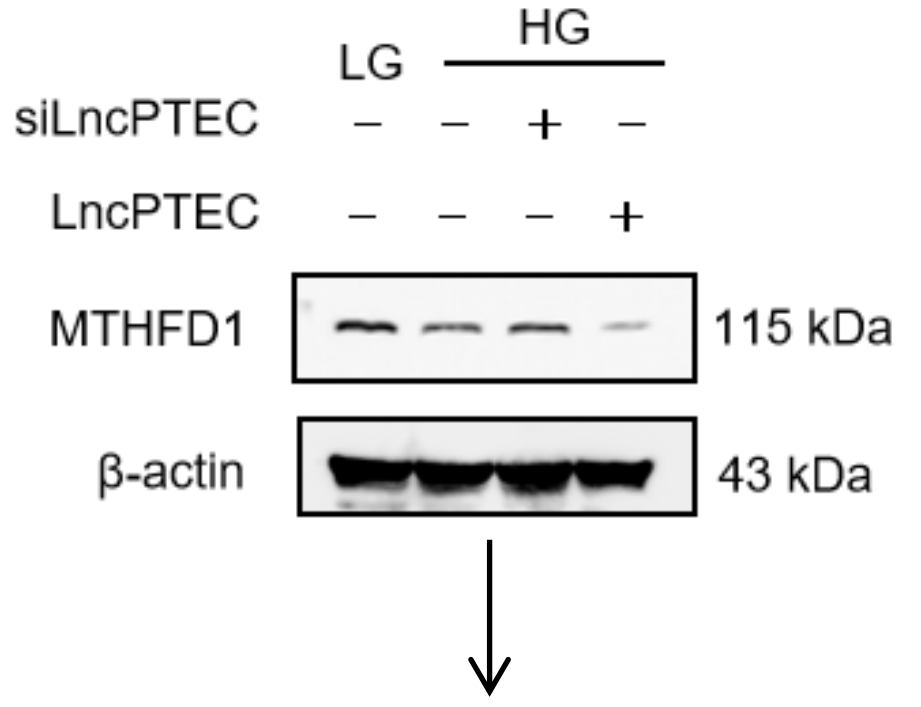

Original unprocessed gel

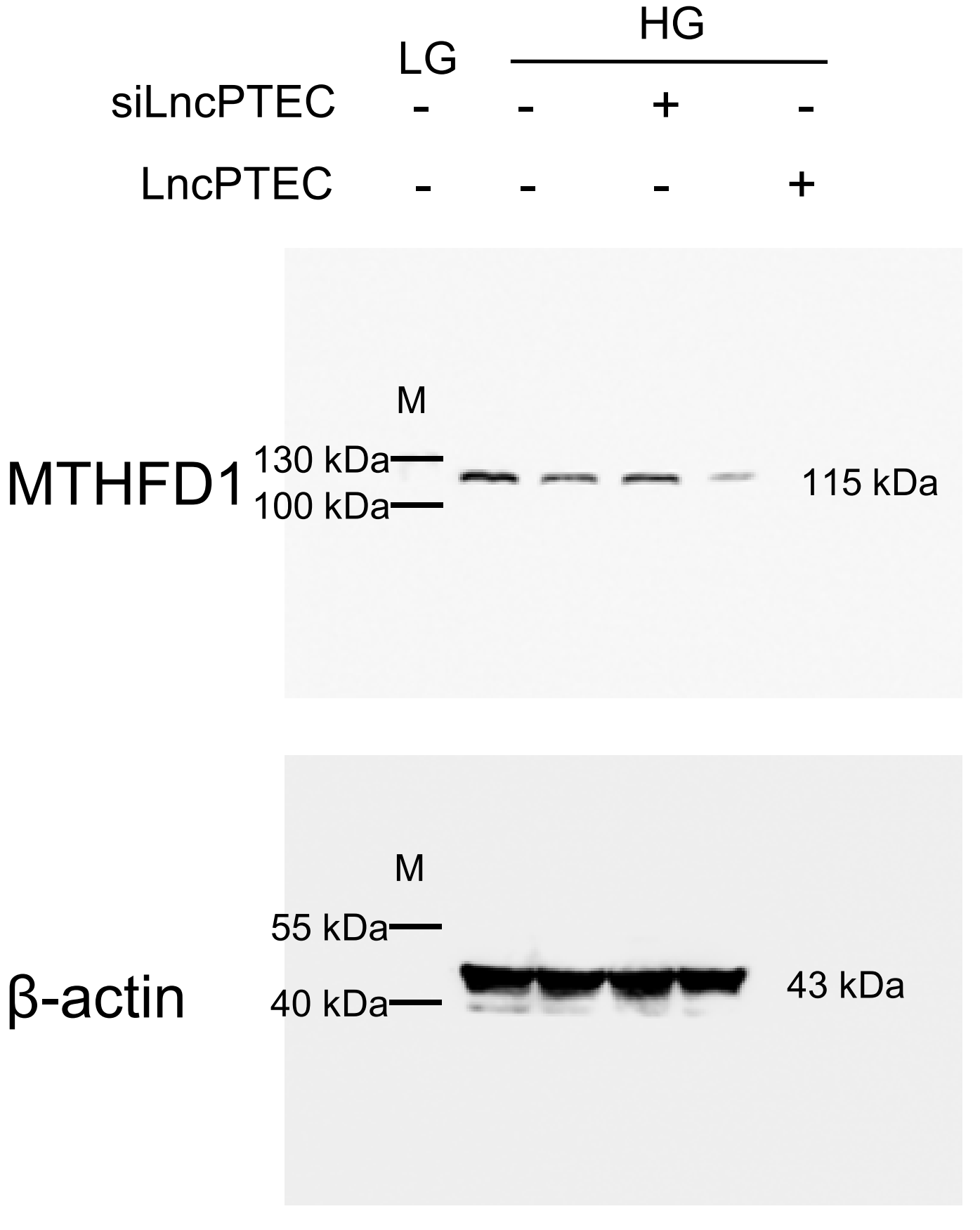

FigS8

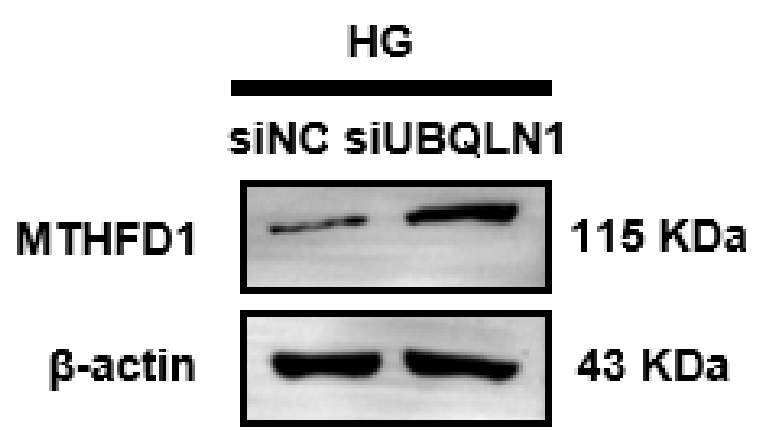

Original unprocessed gel

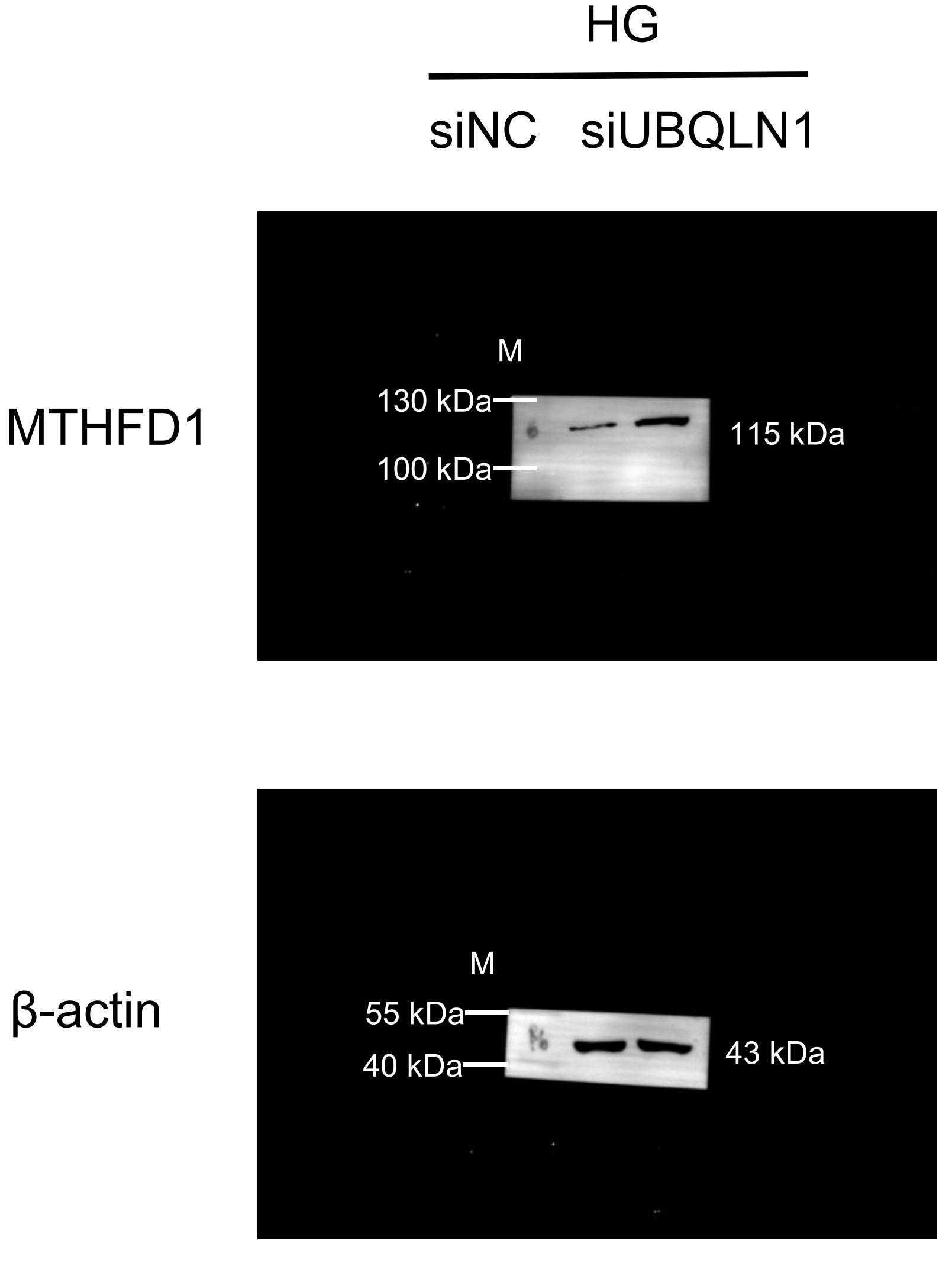

FigS9A

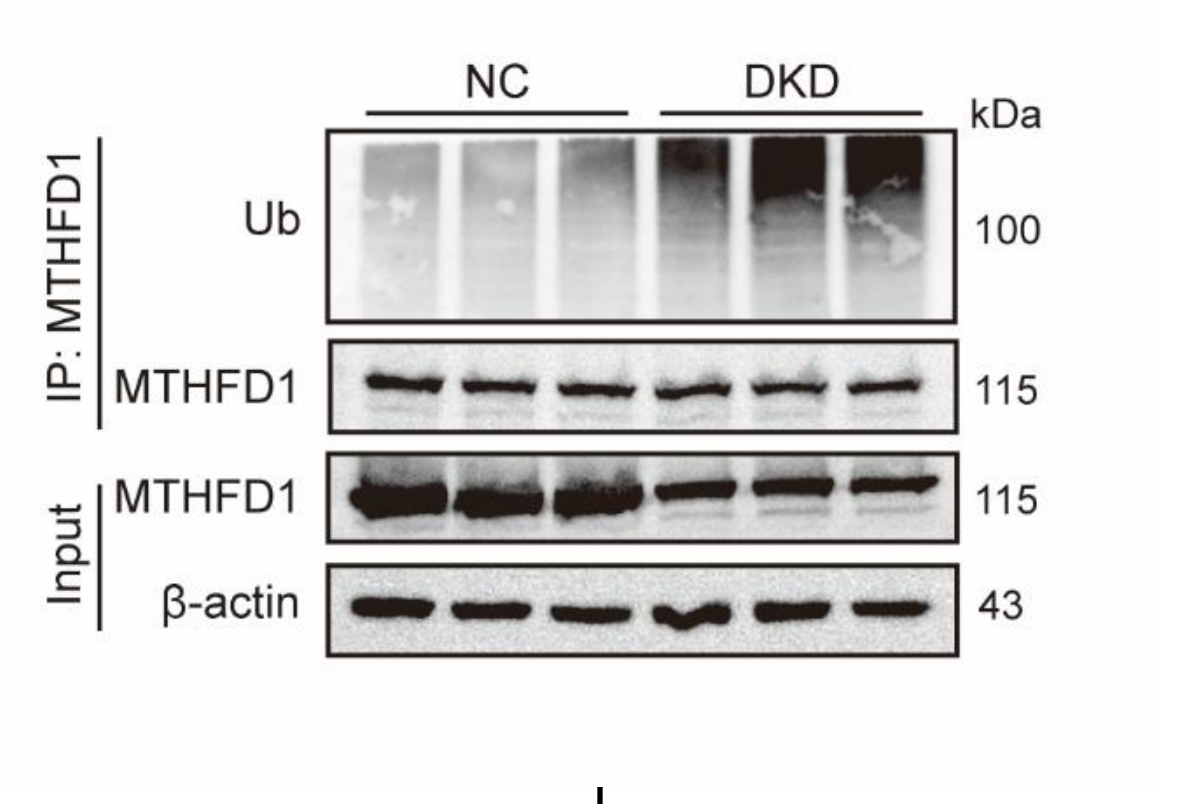

Original unprocessed gel

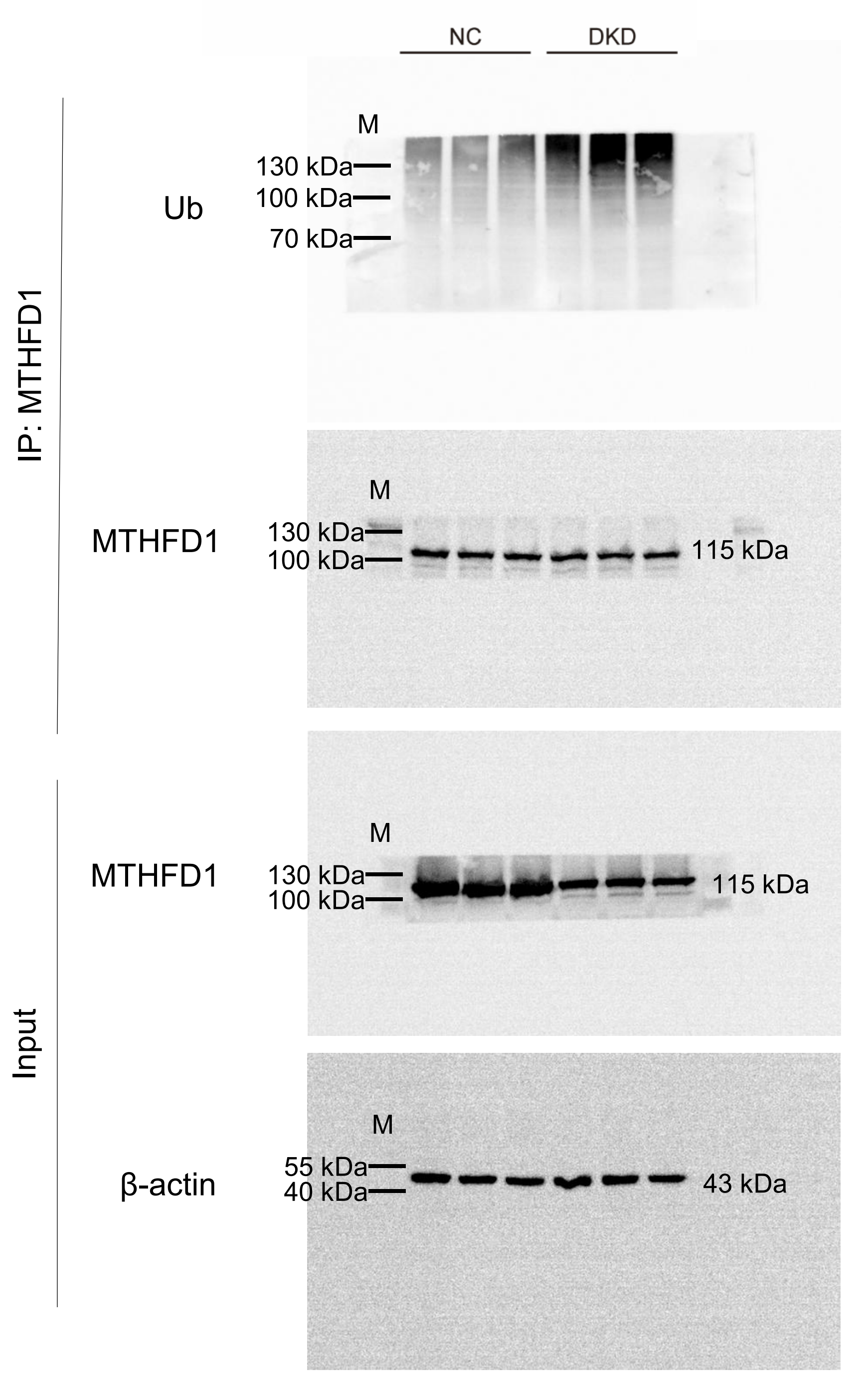

FigS9B

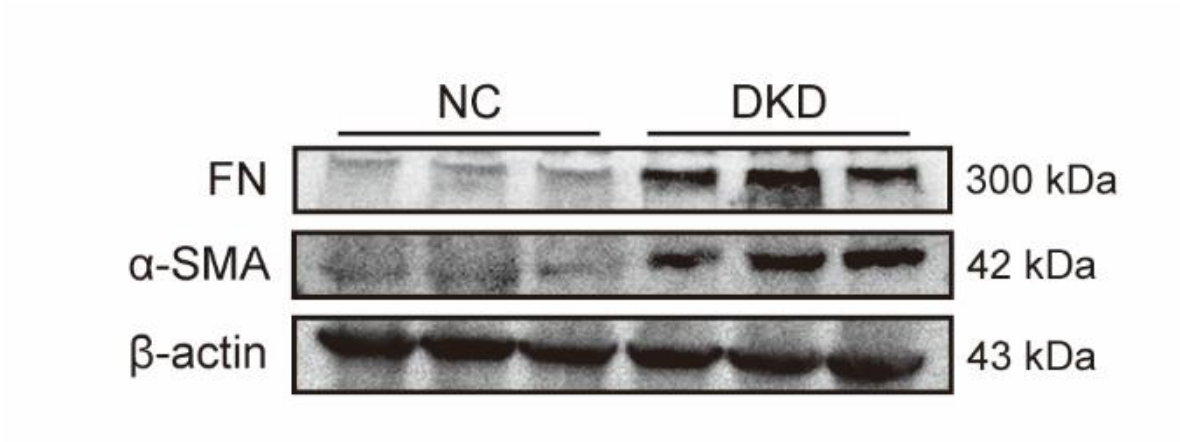

Original unprocessed gel

Fn

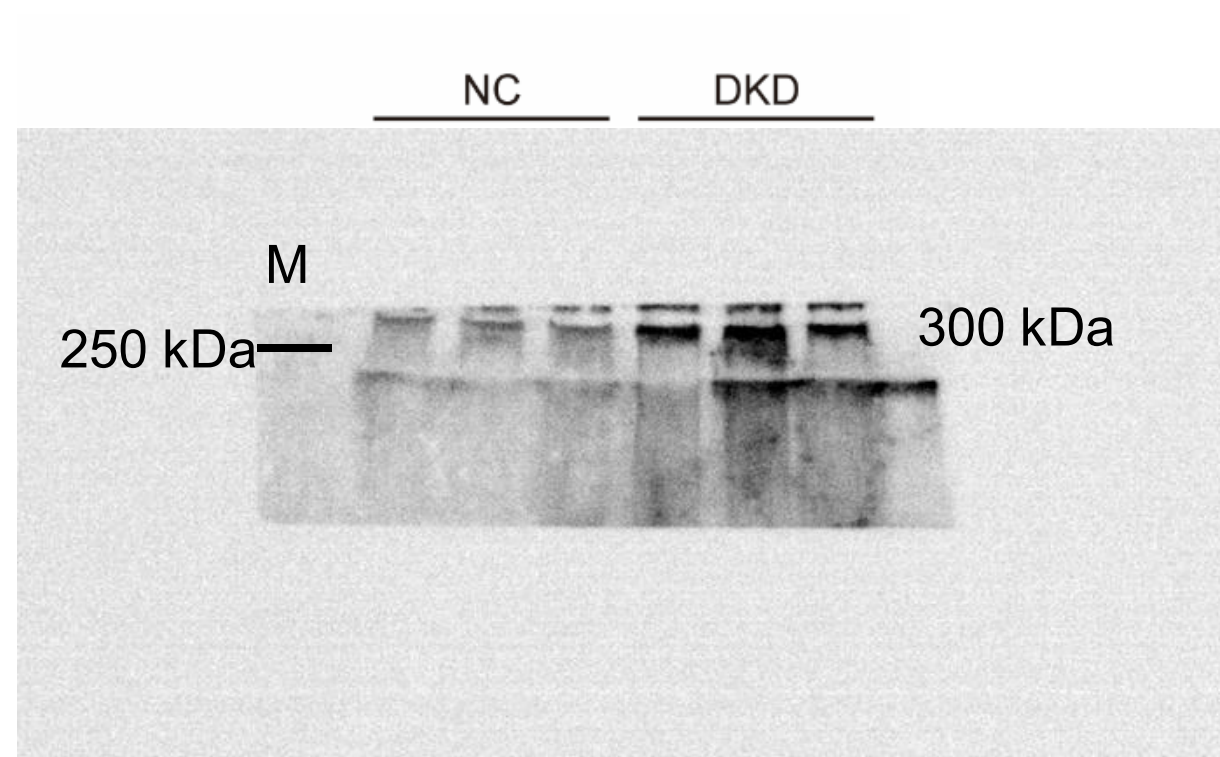

α-SMA

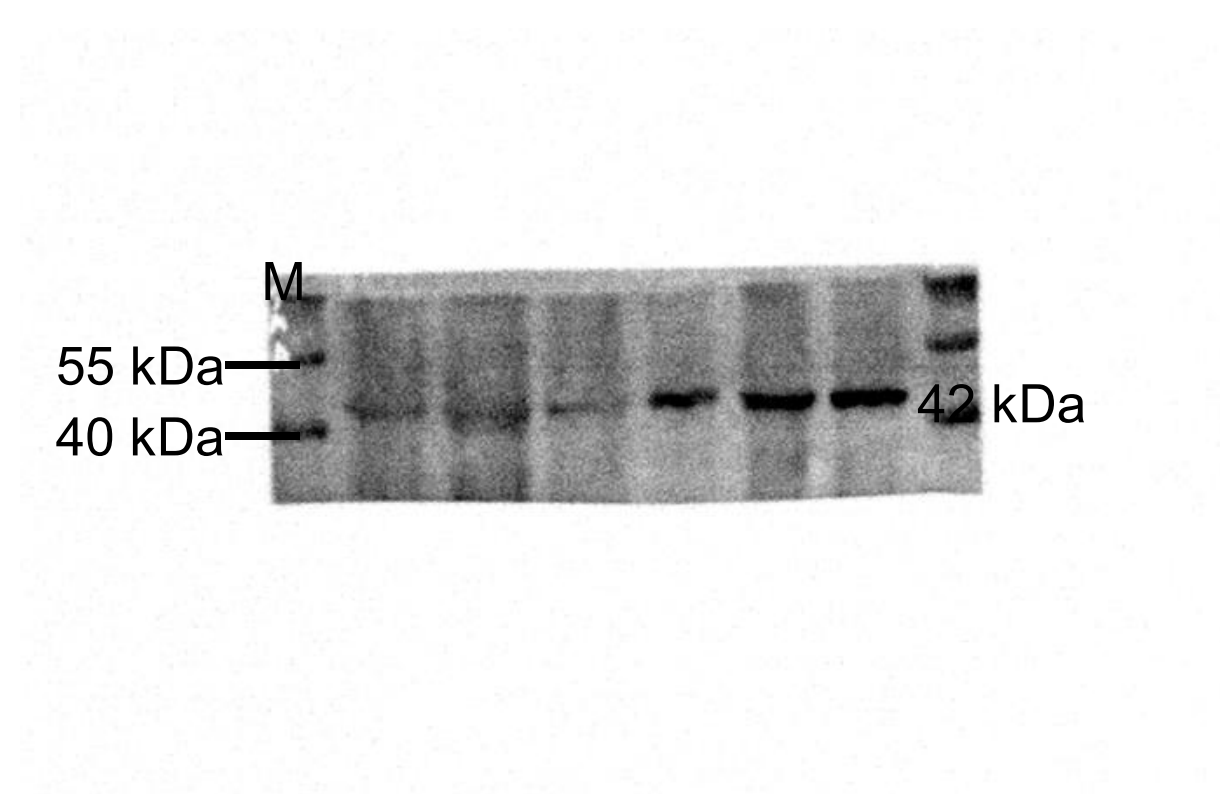

β-actin

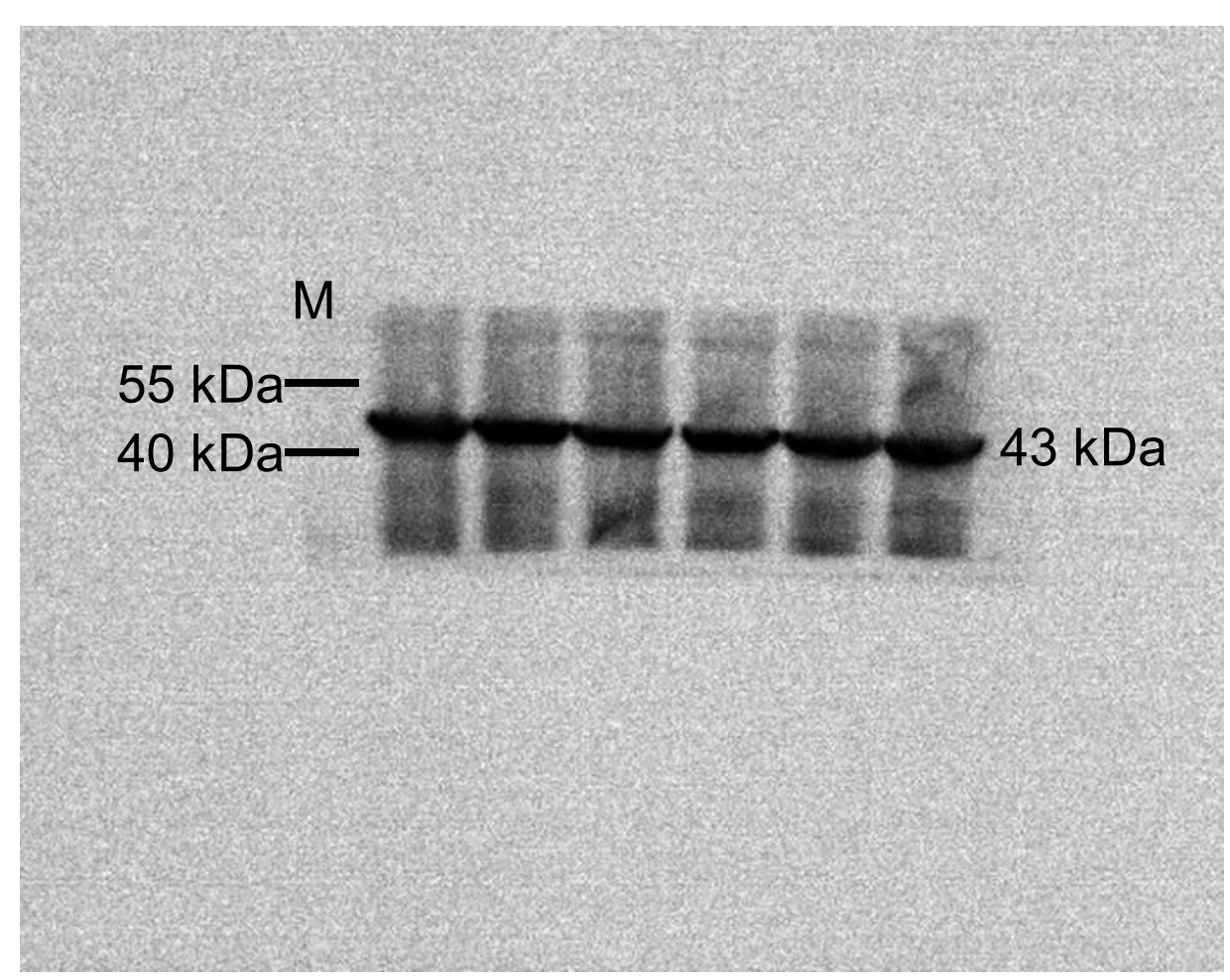

FigS10A

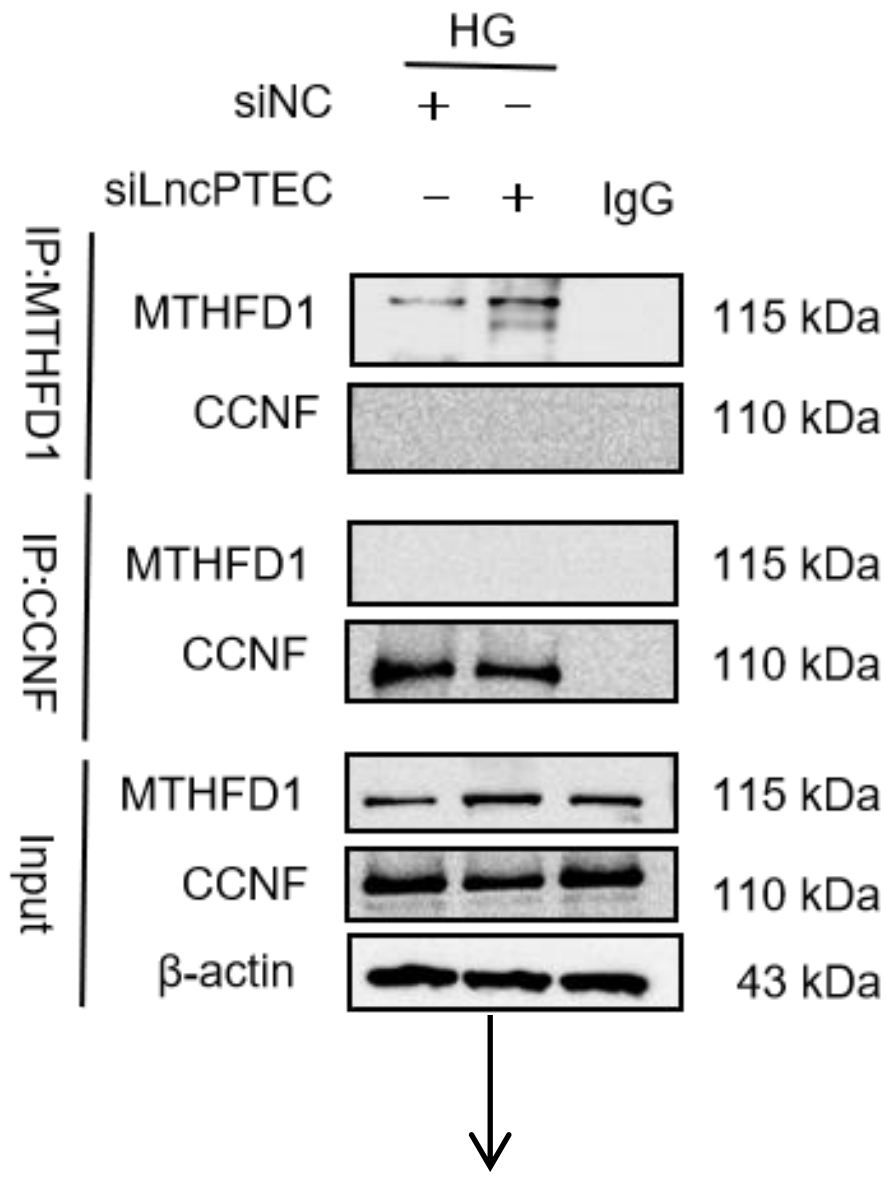

Original unprocessed gel

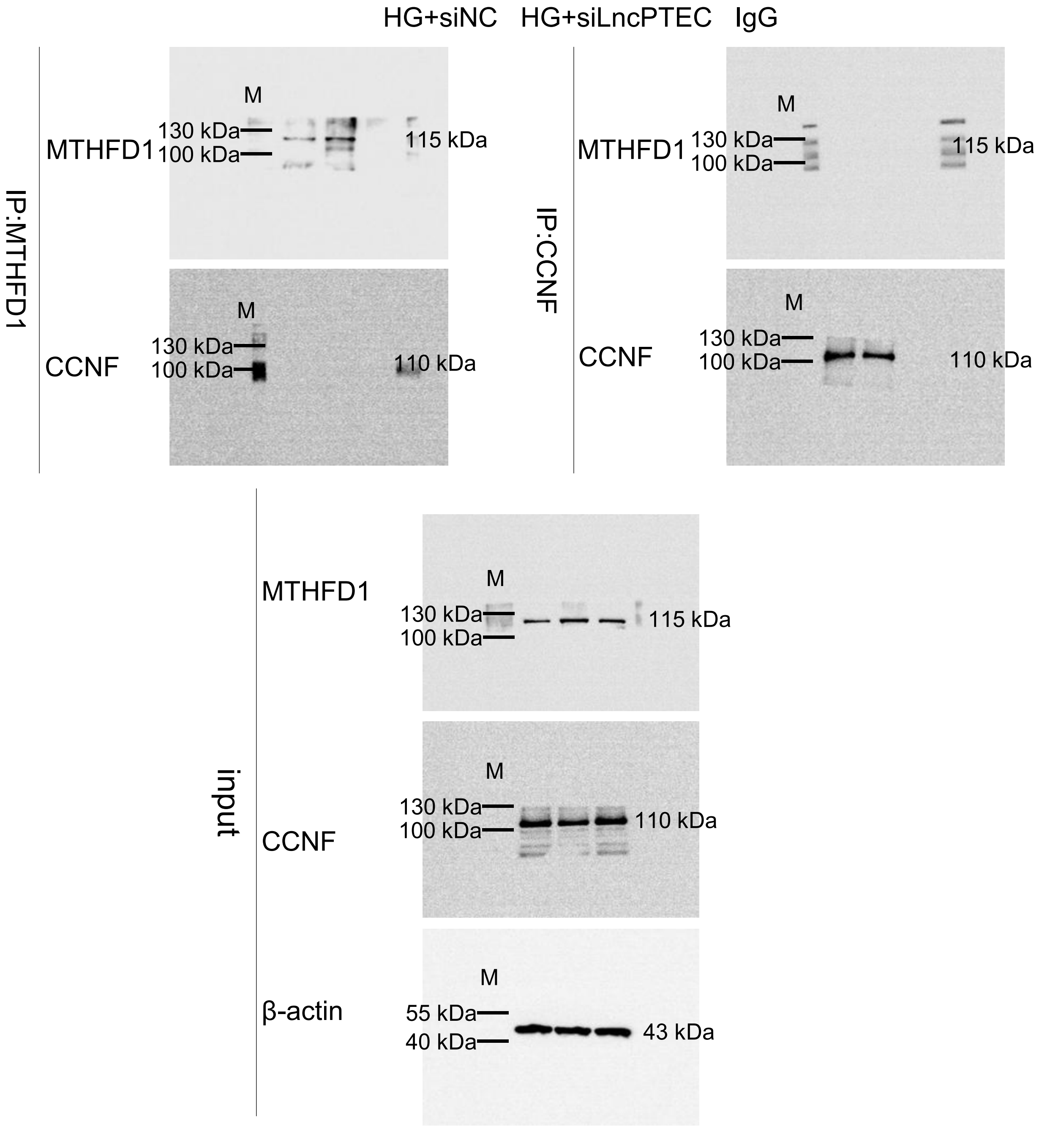

FigS10B

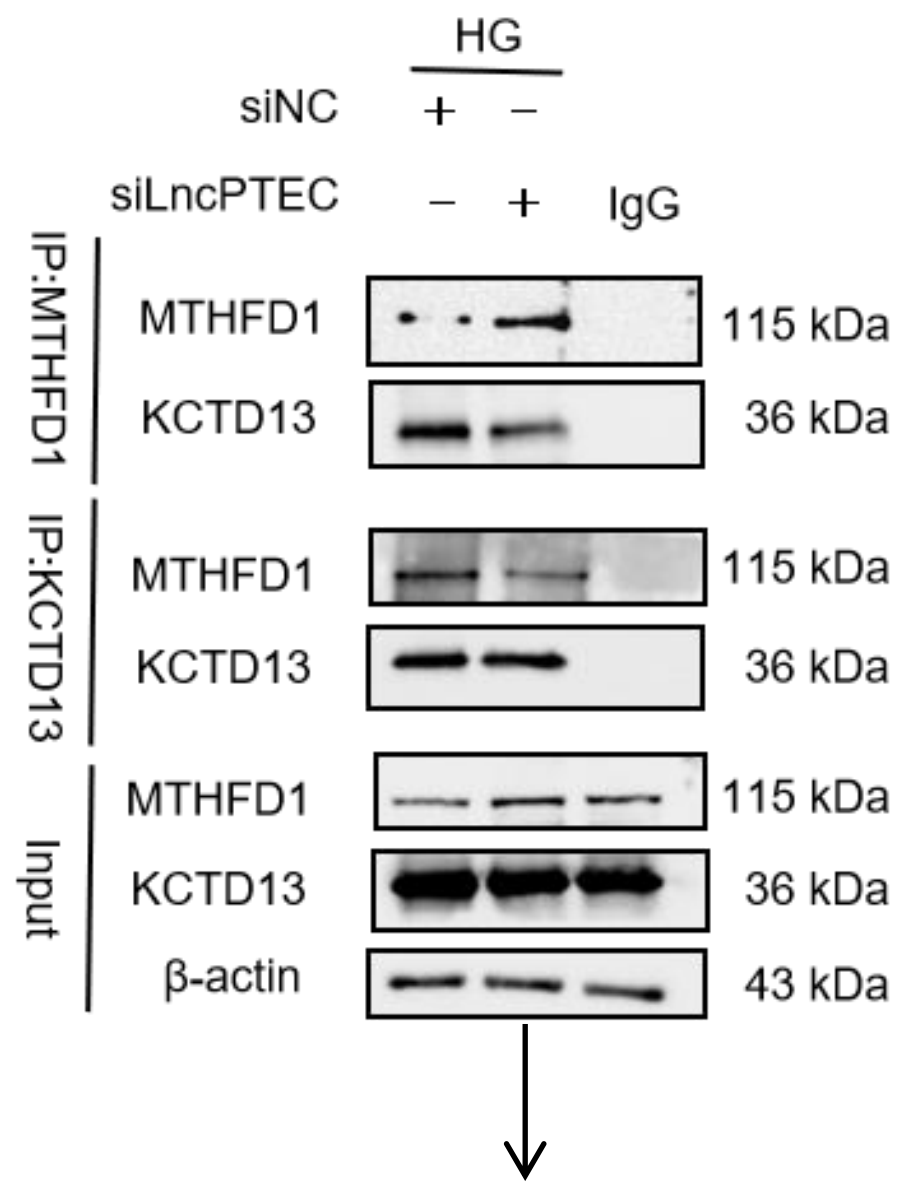

Original unprocessed gel

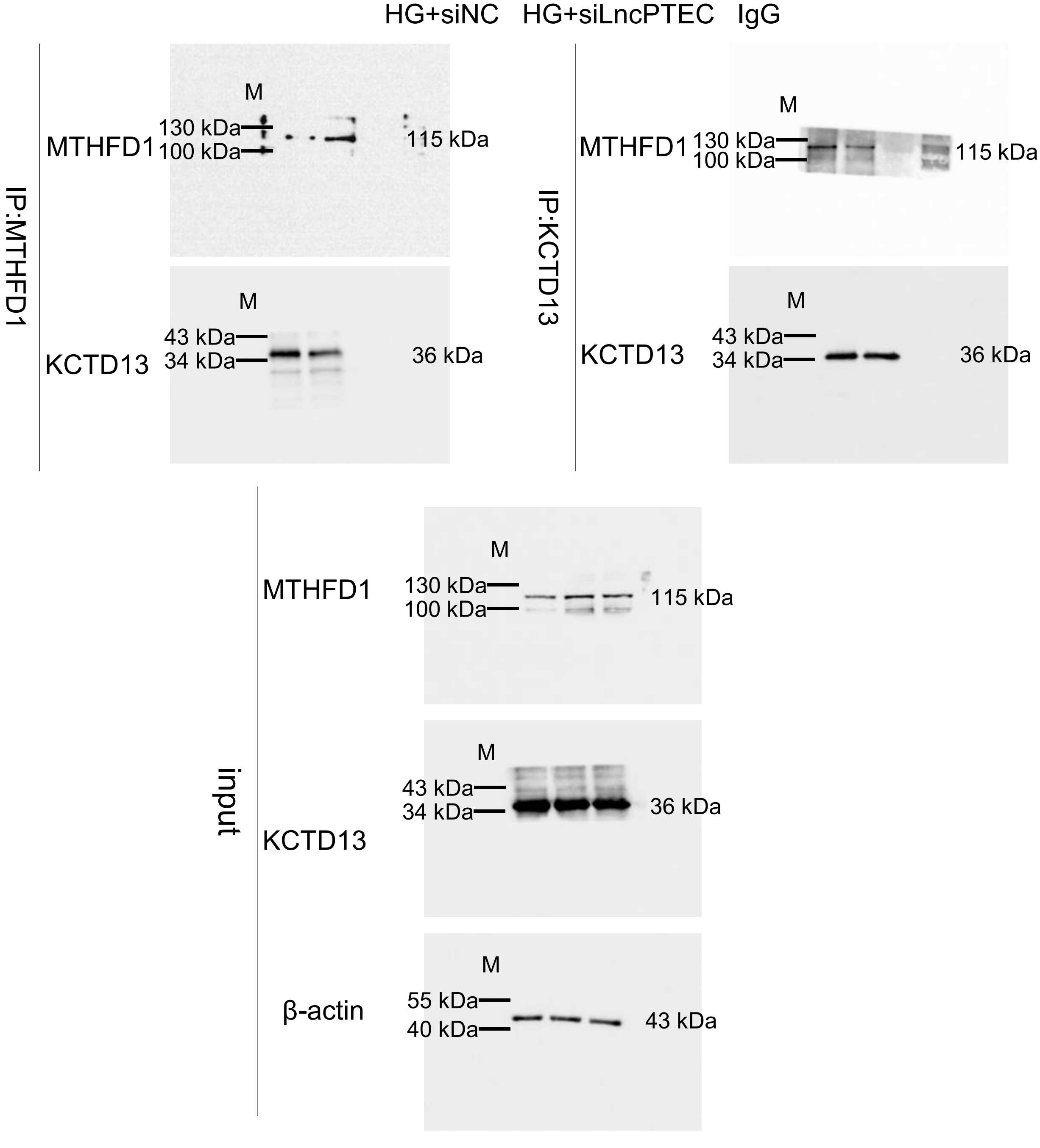

FigS11

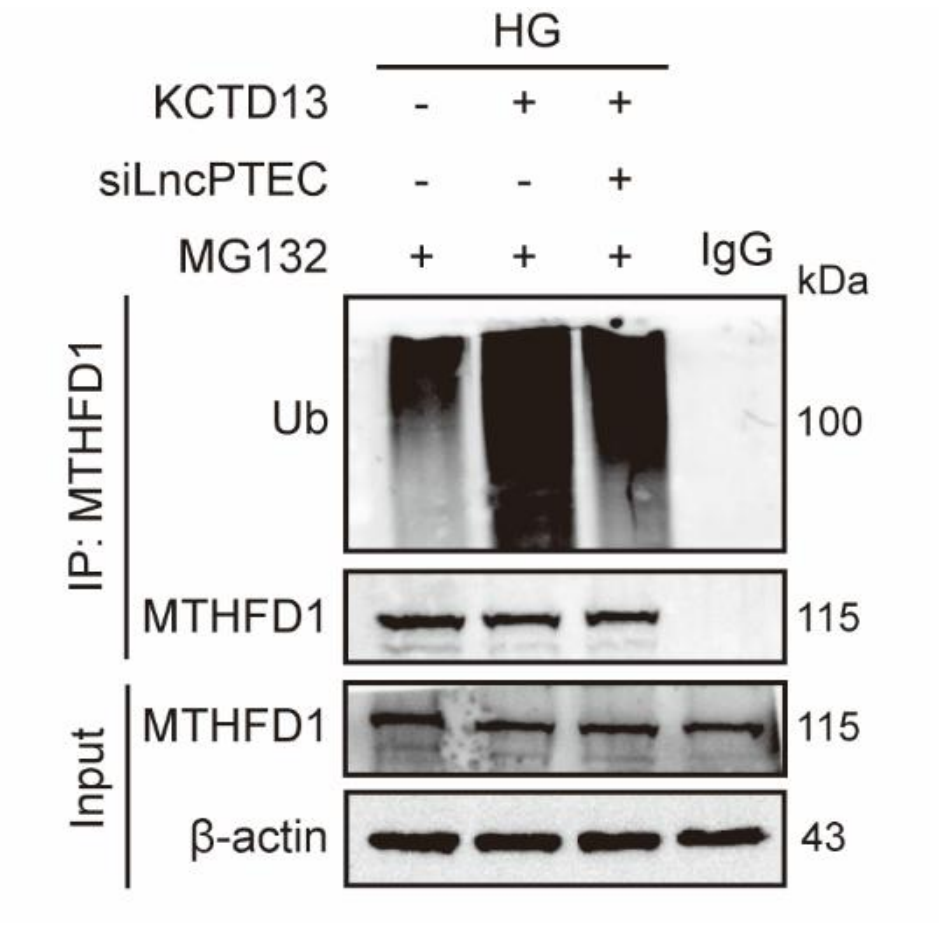

Original unprocessed gel

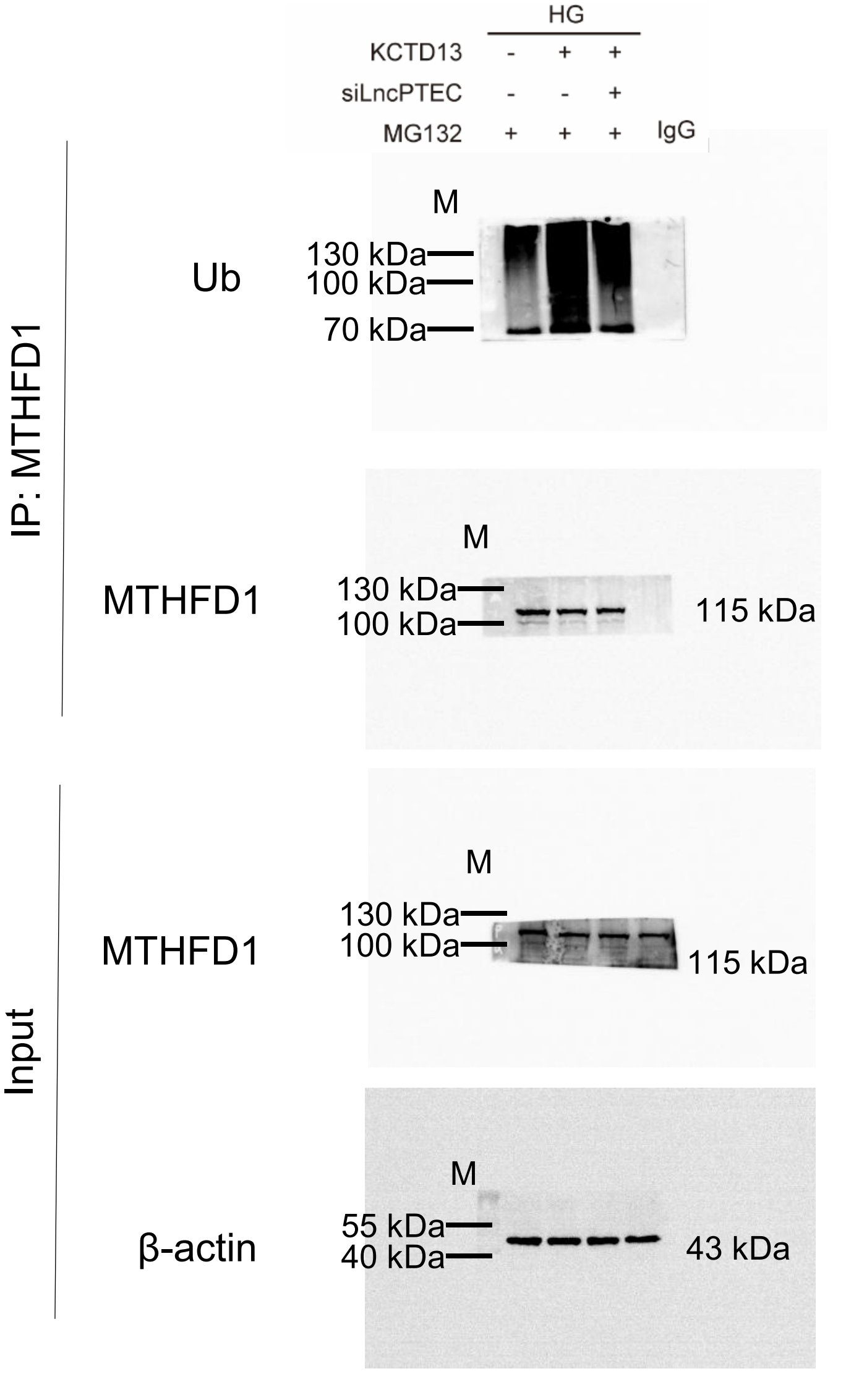

FigS13

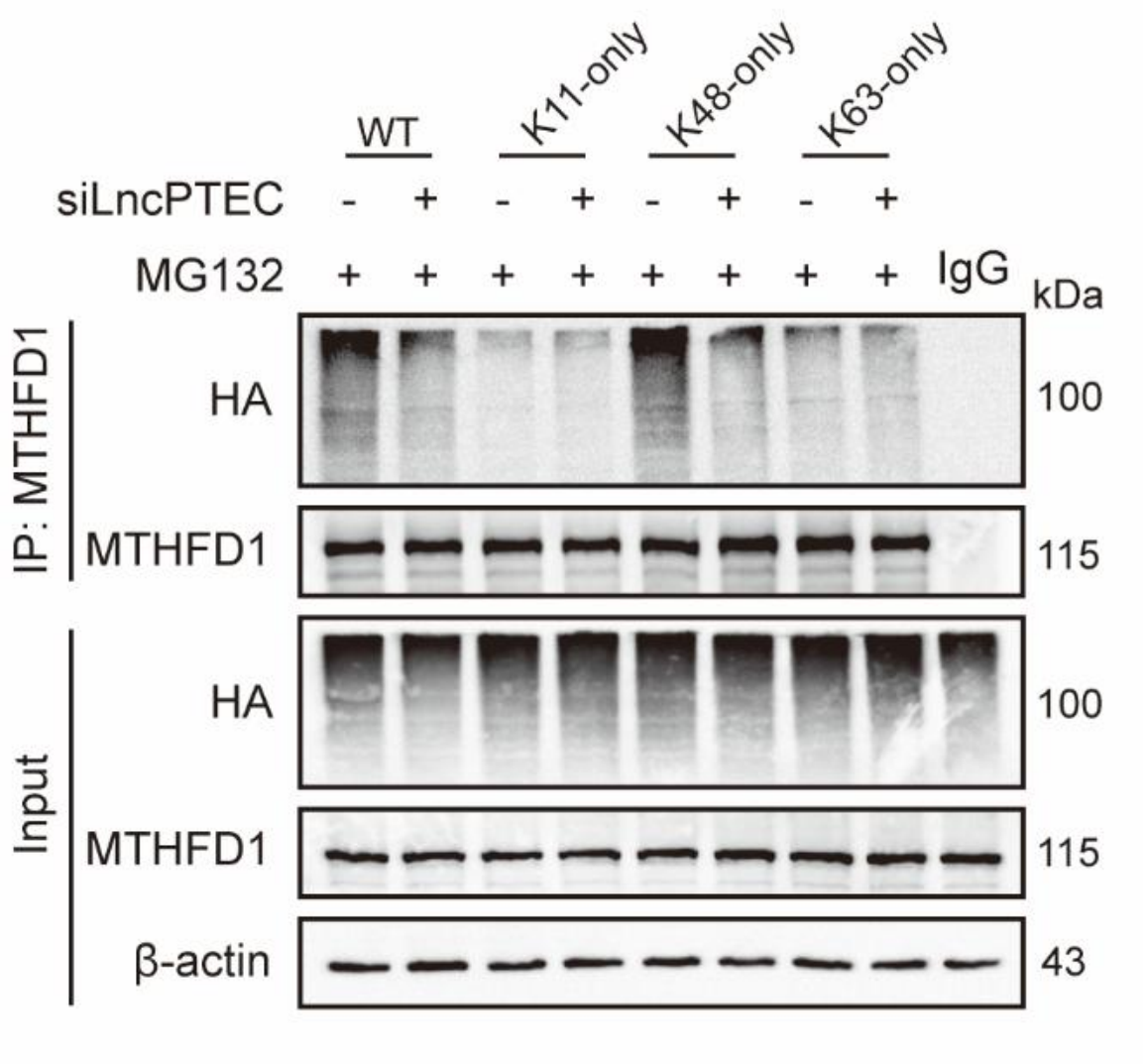

Original unprocessed gel

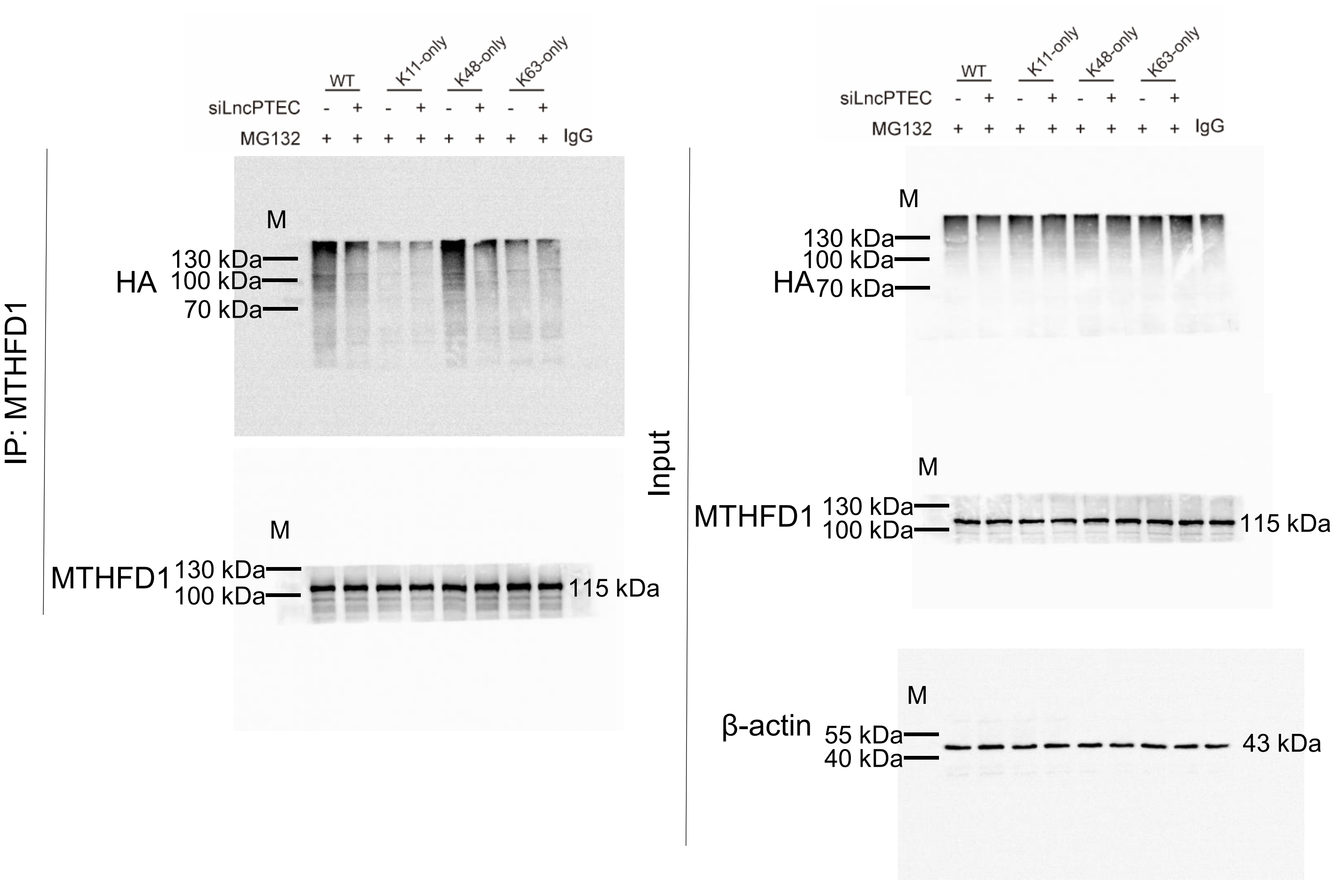

Supplement: Supplementary file 1 [file 41419_2026_9093_MOESM2_ESM.pdf]
