# Supplementary material for: LncPTEC mediated homocysteine accumulation elevates oxidative stress via UBQLN1-dependent MTHFD1 ubiquitination in DKD
Source: Cell Death Dis. 2026 Jul 7;17(1):842. doi: 10.1038/s41419-026-09093-2 (PMC13619569; doi:10.1038/s41419-026-09093-2)
Supplement: Supplementary file 2 [file 41419_2026_9093_MOESM1_ESM.docx]

**Supplementary information**

**LncPTEC mediated homocysteine accumulation elevates oxidative stress via UBQLN1-dependent MTHFD1 ubiquitination in DKD**

Qijia Wang^1,2^, Tianhui Wu^2,3^, Peiling Li^1,2^, He Zha^4^, Keqian Wu^2^, Handeng Liu^5^, Rui Peng^6^, Xuemei Peng^7^, Ziyue Lin^2^, Dan Lv^1^, Xiaohui Liao^1^, Yan Sun^1,2^, Zheng Zhang^1,2^

**Fig. S1.**

**A	B**

**50um**

**Fig. S1. Detection of db/db DKD mice and NC mice related indexes.** (A) Blood glucose, UmAlb and UACR were measured in db/db DKD mice (n=3) and normal control mice (n=3). The data are shown as the mean ± SD. * P<0.05, ** P<0.01 by unpaired t-test. (B) HE, PAS and Masson staining of kidney tissues from db/db DKD mice (n=3) and NC mice (n=3). The scale bar is 50 μm.

**Fig. S2.**

**Fig. S2. Analysis of the differential expression of lncRNAs of DKD and NC groups by RNA-seq.** In the DKD group, the expression of lncPTEC was significantly upregulated. The sample is mouse kidney tissues and n=3 each group, *P*<0.05, log2|FC|>2.

**Fig. S3.**

**Fig. S3. Quantitative analysis of the Western blot in Figure 4F.** Data are presented as mean ± SD. * *P*<0.05, ** *P*<0.01 by unpaired t-test.

**Fig. S4.**

**Fig. S4. The expression level of ROS.** ROS levels were measured by fluorescent dye DCFH-DA with Fluorescent enzyme labeling instrument assays following the transfection of lncPTEC siRNAs or overexpressed lncPTEC under HG conditions. The data are shown as the mean ± SD. * *P*<0.05, ** *P*<0.01, *** *P*<0.001 by unpaired t-test.

**Fig. S5.**

**A**

**B**

**Fig. S5. Construction of siRNAs and overexpression plasmid of SP1.** The expression level of SP1 was tested in si-SP1 and si-NC groups and overexpression plasmid and SP1-NC groups by Western blot (A) and qRT-PCR (B). The data are shown as the mean ± SD. * *P*<0.05, ** *P*<0.01, *** *P*<0.001 by unpaired t-test.

**Fig. S6.**

**Fig. S6. Immunoblotting experiment of MTHFD1.** Immunoblotting was conducted to evaluate and quantify the MTHFD1 protein expression following transfection with lncPTEC over-expression plasmid or lncPTEC siRNAs in HG. The data are shown as the mean ± SD. * *P*<0.05, ** *P*<0.01, *** *P*<0.001 by unpaired t-test.

**Fig. S7.**

**A	B**

**Fig. S7. The expression levels of UBQLN1 in the renal tissues of DKD patients and mice.** (A) The expression levels of UBQLN1 in kidney tissues of DKD patients and controls via the Nephroseq database. The increase in UBQLN1 expression levels is observed in DKD patients (n=7 each group). (B) The expression levels of UBQLN1 by qRT-PCR in the kidney tissues of DKD mice and controls. Compared with the control group, the UBQLN1 level in DKD mice increased (n=6 each group). The data are shown as the mean ± SD. * *P*<0.05, ** *P*<0.01, *** *P*<0.001 by unpaired t-test.

**Fig. S8.**

**A**

**B	C**

**Fig. S8. The detection of MTHFD1 expression, Hcy concentration and ROS level after UBQLN1 silencing in cells with high glucose.** (A) The protein expression level of MTHFD1 was tested in the siUBQLN1 and si-NC groups by Western blot and its quantification. (B) The expression level of Hcy. (C) The expression level of ROS. Data are shown as the mean ± SD. * *P*<0.05, ** *P*<0.01 by unpaired t-test.

**Fig. S9.**

**A	B**

 **C**

**Fig. S9.** **Association between MTHFD1 ubiquitination and renal fibrosis in DKD mice.**

(A) Western blot analysis showing increased ubiquitination levels of MTHFD1 in DKD mice compared with control mice. (B) Expression levels of fibrotic markers Fn and α-SMA in control and DKD mice. (C) Spearman correlation analysis between MTHFD1 ubiquitination levels and the expression of Fn (left) or α-SMA (right).

**Fig. S10.**

**A	B**

**Fig. S10. Co-IP analysis was used to examine the associations between MTHFD1 and CCNF, as well as between MTHFD1 and KCTD13.** (A) Co-IP and immunoblotting analyses revealed that in the presence of high glucose and in the case of knockdown of lncPTEC, MTHFD1 did not bind to CCNF. (B) Co-IP and immunoblotting analyses revealed that, compared to the high glucose group, knockdown of lncPTEC under high glucose conditions led to a reduction in the interaction between MTHFD1 and KCTD13.

**Fig. S11.**

**Fig. S11. Overexpression of KCTD13 promotes MTHFD1 ubiquitination.** Co-IP assay showing the ubiquitination of MTHFD1 in cells overexpressing KCTD13 and/or lncPTEC.

**Fig. S12.**

**A	B	C**

**Fig. S12. The expression levels of KCTD13 in the kidney tissues of patients with DKD, as well as in the kidney tissues and renal tubular epithelial cells of mice.** (A) The expression levels of KCTD13 in kidney tissues of DKD patients and controls via the Nephroseq database. The increase in KCTD13 expression levels is observed in DKD patients (n=10 each group). (B) The expression levels of KCTD13 by qRT-PCR in the kidney tissues of DKD mice and controls. Compared with the control group, the KCTD13 level in DKD mice increased (n=6 each group). (C) The expression level of KCTD13 in the renal tubular epithelial cells of mice in the high glucose group and the control group was detected by qRT-PCR. Compared with the control group, the level of KCTD13 in the cells of the high glucose group was increased (n=6 each group). The data are shown as the mean ± SD. * *P*<0.05, ** *P*<0.01, *** *P*<0.001 by unpaired t-test.

**Fig. S13.**

**Fig. S13. lncPTEC knockdown inhibits K48-linked ubiquitination of MTHFD1.** Co-IP assay showing K11-, K48-, and K63-linked polyubiquitination of MTHFD1 in cells transfected with control or lncPTEC-targeting siRNAs.

**Table S1 The information on patients with DKD and controls**

| Characteristics | Normal control (n=29) | DKD patients (n=41) |
| --- | --- | --- |
| Age (y) | 56.08±9.47 | 58.46±11.72 |
| Height (cm) | 165.28±6.66 | 163.77±7.40 |
| Weight (kg) | 63.70±12.69 | 68.7±10.75 |
| Sex (♀/♂) | 11 / 18 | 14/ 27 |
| HBALC (%) |  | 9.04±2.07 |
| Urinary albumin (mg/L) | - | 128.46±157.53 |
| Urinary creatinine (umol/L) | - | 10668.51±6232.79 |
| UACR (mg/g) | < 30 | 150.06±304.07 |

**Table S2 Primer sequence**

| **Genes** | **Primer sequence** |
| --- | --- |

F: 5’-GCTCAGTAACAGTCCGCCTAG-3 ’

β-actin

R: 5’-AGTGTGACGTTGACATCCG-3 ’

F: 5’-GGTTGTCTCCTGCGACTTCA-3 ’

GAPDH

R: 5’-TGGTCCAGGGTTTCTTACTCC-3 ’

F: 5’-CTCGCTTCGGCAGCACA-3 ’

U6

R: 5’-AACGCTTCACGAATTTGCGT-3 ’

F: 5’-TGAGCAGGAAGGGTGGACAGAC-3 ’

Gm16193

R: 5’-AAGAGCAGTTTGAAGCAGGTAGTAGC-3 ’

F: 5’-GGGTGTTGGGTAAGTTGGAGTTGAG-3 ’

Gm36011

R: 5’-TGTTGATGCTTAAGAGGACCACTGTTC-3 ’

F: 5’-TTGAGGCAGCAGAGGAGGAAGAG-3 ’

Gm20400

R: 5’-CCGGCCAATGCACCAAATGAAAG-3 ’

F: 5’-GAAGTCAGGACAGGAACTCAATCAGG-3 ’

Gm9968

R: 5’-AGAGCAAGCCAGTAAGCATCAACC-3 ’

F:5’-ATGGACTGGACCTCTGAACCTCTTC-3 ’

Gm32569

R: 5’-CACGGGAGCAAATGTTGTTGTCAC-3 ’

F:5’-TGCCAATGTTGCAAGAAGCG-3 ’

D630024D03Rik

R: 5’-GAGCACTTAAGTTGCCTGCC-3 ’

F:5’-TGACTGCTGTCTGCTCATTGCTC-3 ’

AI314278

R: 5’-AATCGCTGACTGACCTAACTTGGATG-3 ’

F:5’-GCTTGCCAACACCAACATCG-3 ’

Gm16311

R: 5’-ACAAACAGAAAAGCCCCCGT-3 ’

F:5’-TTTCAAGGCAGCTGGGAACA-3 ’

Gm15347

R: 5’-CTGTCCCCTCACAAAGCCAT-3 ’

F:5’-ATCCAGTGCCTACCAGACAAT-3 ’

Gm15962

R: 5’-TCACGCCTCTGTAAGTGTGAA-3 ’

F:5’-TGCTCAGGAAGAGTGACCAAATACAAG-3 ’

4930517O19Rik

R: 5’-CGATGCTGTCATGTCTCAGTGTCTAC-3 ’

F:5’-ACACCTGTGATGGACGGACTCTC-3 ’

4931403E22Rik

R: 5’-ACGGGTGACCTTTGTGGCTTTG-3 ’

F:5’-ACCTCCAGCTCAATGTTATCATCAGTG-3 ’

1700112D23Rik

R: 5’-AAGGGTTCTAAGAGCCGTTCATGTG-3 ’

F:5’-GCTGGTGACATGCCAAGATG-3 ’

Hnf4aos

R: 5’-CGTGCTCCACGTGAAAACAT-3 ’

F:5’-TGGACCTGGGCTTGGTACAT-3 ’

Gm37245

R: 5’-TTTTACCGGCACCACCTTGG-3 ’

F:5’-CAGACCACCTCGTTGTGCTA-3 ’

Gm16599

R: 5’-GTGCATGTGAAGTCTTGCCG-3 ’

F:5’-TCTTCCTGAATGGACCCAAGC-3 ’

Gm26854

R: 5’-AAGTATGTGGCGCTCCCTTT-3 ’

F:5’-CAGTGCCAGGTCCGTTGAG-3 ’

Gm26827

R: 5’-CAGCTGCAGAGGCGGC-3 ’

F:5’-GTATGGGATCCGCTGTGAGG-3 ’

E230001N04Rik

R: 5’-GAGAAGGTGCCGATCCTAGC-3 ’

F:5’-AAGTGGTCAGGCTTGCATGG-3 ’

Gm16833

R: 5’-AGCCACTTCTCAGTCTCTGGA-3 ’

F: 5’-TTCCACAACCACCTCAAGCACTTC-3 ’

E-Ca

R: 5’-ATTAAGGCATCACAGTCCGAGTCAC-3 ’

F: 5’-ACCATGCCTAAAGCTGTCC-3 ’

zo-1

R: 5’-GGAACTCAACACACCACCA-3 ’

F: 5’-CCGTGGACCAGGTTGATGATACTTC-3 ’ Fn

R: 5’-GCTCTGTGCTACTGCCTTCTACTG-3 ’

F: 5’-TCCAGGTTTCCCTACTCCA-3 ’

Col-Ⅳ

R: 5’-GGGCTCCATCTCTTCCAC-3 ’

H-AC061708. 1	F: 5’-ACTTGCGCCTTCCAACTCTTA-3 ’

|  | R: 5’-CATTGGGTGCTTTGCTTGGTT-3 ’ |
| --- | --- |
|  | F: 5’-TCTCCCAAGTCCACACAGG-3 ’ |
| HS-ACTB |  |
|  | R: 5’-GGCACGAAGGCTCATCA-3 ’ |
| Vim | F: 5’-CTCCTACGATTCACAGCCA-3 ’  R:5’-GAGCCACCGAACATCCT-3 ’ |
| α-SMA | F: 5’-GCCCAGAGCAAGAGAGG-3 ’  R:5’-TGTCAGCAGTGTCGGATG-3 ’ |
